# Total synthesis of the proposed structure of astakolactin

**DOI:** 10.3762/bjoc.10.252

**Published:** 2014-10-17

**Authors:** Takayuki Tonoi, Keisuke Mameda, Moe Fujishiro, Yutaka Yoshinaga, Isamu Shiina

**Affiliations:** 1 Department of Applied Chemistry, Tokyo University of Science,1-3 Kagurazaka, Shinjuku-ku, Tokyo 162-8601, Japan

**Keywords:** aldol reaction, astakolactin, lactonization, MNBA, terpenoids

## Abstract

The first total synthesis of the proposed structure of astakolactin, a sesterterpene metabolite isolated from the marine sponge *Cacospongia scalaris*, has been achieved, mainly featuring Johnson–Claisen rearrangement, asymmetric Mukaiyama aldol reaction and MNBA-mediated lactonization.

## Introduction

**Figure 1:**
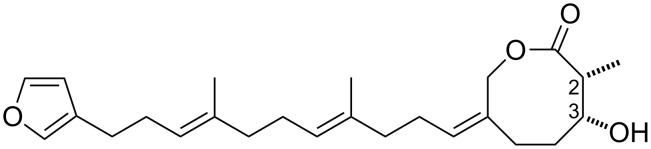
Proposed structure of astakolactin (**1**).

Astakolactin (**1**) is a novel sesterterpene metabolite [1–5] first reported in 2003 by Roussis et al [6]. It was isolated from the marine sponge *Cacospongia scalaris,* which was collected from the gulf of Astakos in the Ionian Sea near Greece. The structure proposed for compound **1** is a bicyclic linear sesterterpenoid bearing a furan unit and an eight-membered lactone tethered by a non-conjugated triene chain (Figure 1). Although the proposed structure of compound **1** is unusual, given that a number of compounds isolated from sponges of the same species have a steroid-like structure [7–9], the structure does resemble that of marine furanosesterterpene natural products, such as variabilin [10–11], which exhibit a large spectrum of intriguing biological activities [12–13]. Therefore, compound **1** is also expected to possess biological activities similar to those of other furanosesterterpenes.

In addition, medium-sized lactones (8–11 membered rings) are significant structural motifs because they are found in many natural products possessing useful biological activities [14–19]. Among them, the 8-membered (and 9-membered) lactone framework is rather unusual in nature [20–24]. We have already developed the synthetic method of lactones with various ring sizes using 2-methyl-6-nitrobenzoic anhydride (MNBA) under mild reaction conditions in the presence of a nucleophilic catalyst such as 4-(dimethylamino)pyridine (DMAP) [25–26]. By using this method, we have demonstrated that unusual saturated medium-sized lactones, which are generally difficult to construct because of the ring strain and transannular interactions [27–28], can be effectively prepared [29–30]. Therefore, in order to determine the chemical structure and the expected biological activity of compound **1**, we executed the total synthesis of compound **1** by exploitng the MNBA-mediated lactonization for the formation of its 8-membered lactone moiety.

## Results and Discussion

**Scheme 1:**
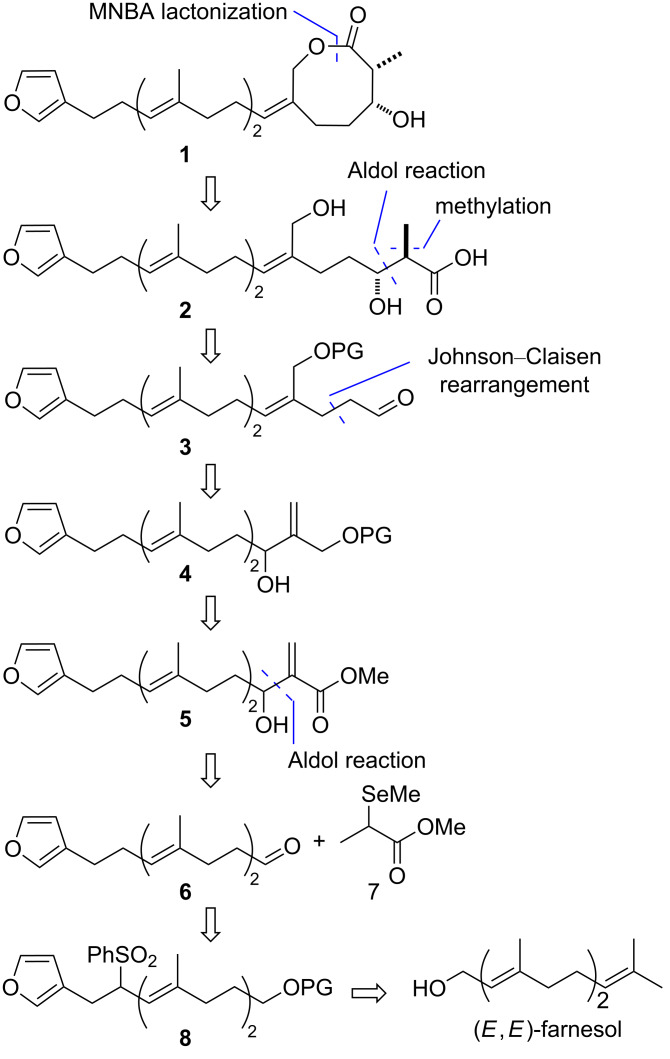
Retrosynthetic analysis.

The rationalized synthesis of **1** primarily involved the linear synthesis of the prenyl chain precursor from commercially available (*E*,*E*)-farnesol to form the corresponding seco-acid and the subsequent construction of the 8-membered lactone moiety. The retrosynthetic analysis of **1** is depicted in Scheme 1 [31].

First, the 8-membered ring in **1** could be efficiently constructed via lactonization using MNBA with DMAP. The chain precursor **2** would be constructed via an aldol reaction with ethyl acetate, followed by *anti*-selective methylation [32–33]. The trisubstituted alkene moiety of the prenyl chain of **3** could be stereoselectively constructed via a Johnson orthoester–Claisen rearrangement of **4** [34–36], which would be generated from compound **5**. The construction of the α-methylene-β-hydroxy ester moiety in **5**, which is commonly known as a Morita–Baylis–Hillman adduct, would be achieved via an aldol reaction between aldehyde **6** and ester **7**, followed by oxidative deselenization [37]. The linear precursor **8** would be prepared from (*E*,*E*)-farnesol using the previously reported method based on the Julia–Kocienski-type transformation [11].

**Scheme 2:**
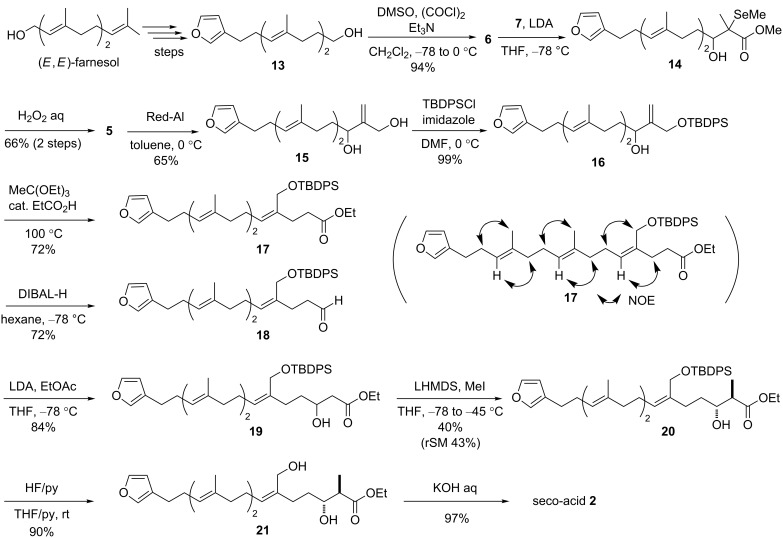
Synthesis of 2,3-*cis*-astakolactin.

The synthetic route to **1** is depicted in Scheme 2. It commenced from the readily accessible furanylated alcohol **13** [11], which was prepared from (*E*,*E*)-farnesol via a 10-step sequence. First, oxidation of **13** provided aldehyde **6**. The aldol reaction of **6** with ester **7** in the presence of base and the subsequent oxidative deselenization of the resulting adduct **14** yielded a Morita–Baylis–Hillman-type product **5** [37] in two steps. Reduction of **5** and the regioselective protection of the less-hindered primary hydroxy group in the resulting diol **15** gave the monoprotected alcohol **16**. This alcohol was then subjected to the Johnson orthoester–Claisen rearrangement, yielding only the (*Z*)-isomer **17** [36] in satisfactory yield. After the reduction of **17**, the resulting aldehyde **18** was subjected to the aldol reaction with ethyl acetate to afford the adduct **19**. Diastereoselective methylation of the ester enolate moiety of **19** with MeI afforded only the *anti*-product **20** [32–33], which was then subjected to the deprotection of the TBDPS group with HF·pyridine, followed by cleavage of the ethyl ester group in **21** to give the desired seco-acid **2** in high yield.

**Scheme 3:**
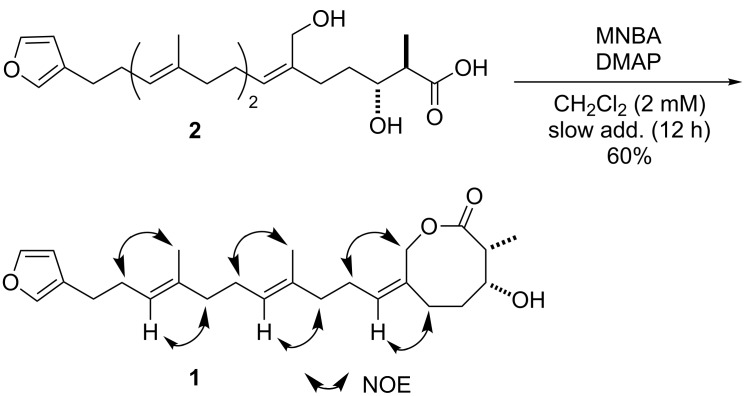
MNBA-mediated lactonization.

Eventually, the lactonization of **2** was performed under several sets of reaction conditions (Scheme 3 and Table 1). We preliminarily attempted the Yamaguchi mixed-anhydride method [38] with 2,4,6-trichlorobenzoyl chloride (TCBC) as an activating agent, which is one of the best-known lactonization methods so far. However, the desired lactone **1** was obtained in a lower yield (33%, Table 1, entry 1) accompanied by unidentified complex products. Moreover, the *S*-pyridyl ester lactonization method [39] afforded no desired product at all as shown in Table 1, entry 2. Next, the MNBA-mediated lactonization [25–26,29–30] was then carried out; in sharp contrast to the first two methods, only the targeted 8-membered lactone **1** was selectively obtained in moderate yield (60%, Table 1, entry 3), and formation of the undesired β-lactone was avoided. Furthermore, the lactonization yield was improved from 60 to 71% by decreasing the substrate concentration from 2.0 to 1.0 mM (Table 1, entry 4). During the MNBA-mediated lactonization, no epimerization occurred at the α position of lactone carbonyl group. Thus, the total synthesis of the proposed structure of **1** was achieved.

**Table 1:** Yields of astakolactin (**1**) using several lactonizations.

Entry	Reagents (equiv)	Conditions	Yield/%^a^

1	TCBC (1.0)/Et_3_N (1.1)/DMAP (6.0)	THF/CH_2_Cl_2_ (2 mM) slow add. (12 h)	33
2	(PyS)_2_ (1.5)/PPh_3_ (1.6)/Ag(OTf) (2.0)	CHCl_3_/MeCN (2 mM) slow add. (12 h)	N.D.
3	MNBA (1.3)/DMAP (6.0)	CH_2_Cl_2_ (2 mM) slow add. (12 h)	60
4	MNBA (1.3)/DMAP (6.0)	CH_2_Cl_2_ (1 mM) slow add. (12 h)	71

^a^Isolated yield.

**Scheme 4:**
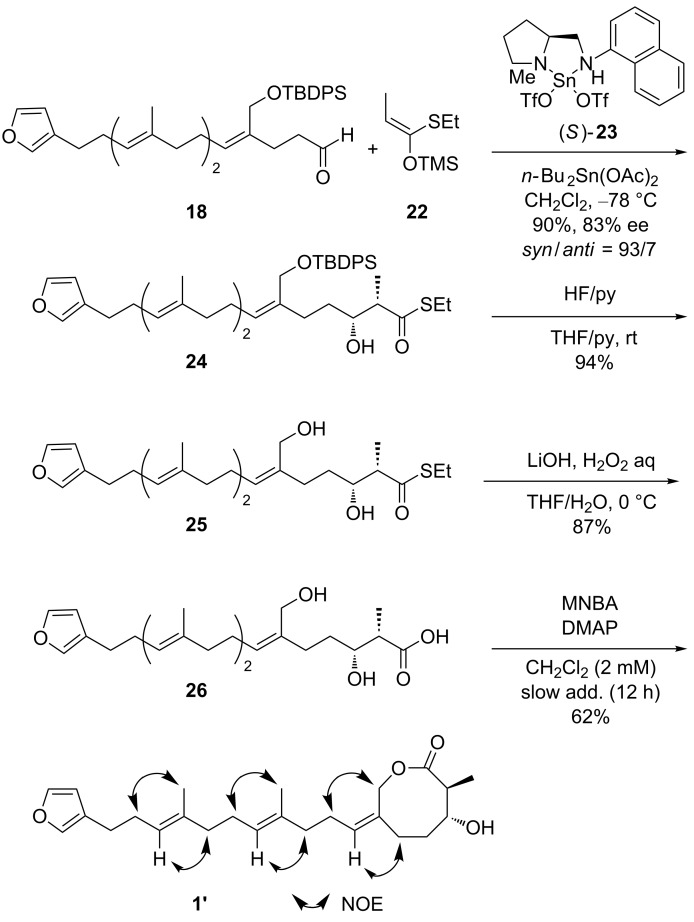
Synthesis of 2,3-*trans*-astakolactin.

We next focused on the synthesis of the diastereomer of **1**, 2,3-*trans*-astakolactin (**1’**), to thoroughly uncover its molecular structure and stereochemistry, starting from the aldehyde **18**, which was prepared according to the procedure [11] described in Scheme 2. It was postulated that the precursor **26** (Scheme 4) of the ring-closed product (**1’**) possessing a *syn*-α-methyl-β-hydroxy carboxylic acid moiety could be accessed via an asymmetric Mukaiyama aldol reaction [40–42] and our universal lactonization strategy. The synthetic route to **1’** is shown in Scheme 4.

The asymmetric Mukaiyama aldol reaction of aldehyde **18** with enol silyl ether **22** (derived from *S*-ethyl propanethioate) in the presence of (*S*)-diamine-Sn(II) complex (*S*)-**23** as the catalyst and *n*-Bu_2_Sn(OAc)_2_ proceeded smoothly to afford the corresponding aldol **24** as the desired (2,3)-*syn* adduct in good yield and excellent enantio- and diastereoselectivities (90% yield, 83% ee for *syn*, and 93/7 *syn*/*anti* ratio). Next, the transformation to the seco-acid **26** was achieved via successive deprotection of the TBDPS group and the thioester moiety in **25**. The lactonization of **26** was then performed in the presence of MNBA and DMAP to afford **1’** with the desired stereochemistry in 62% yield.

**Figure 2:**
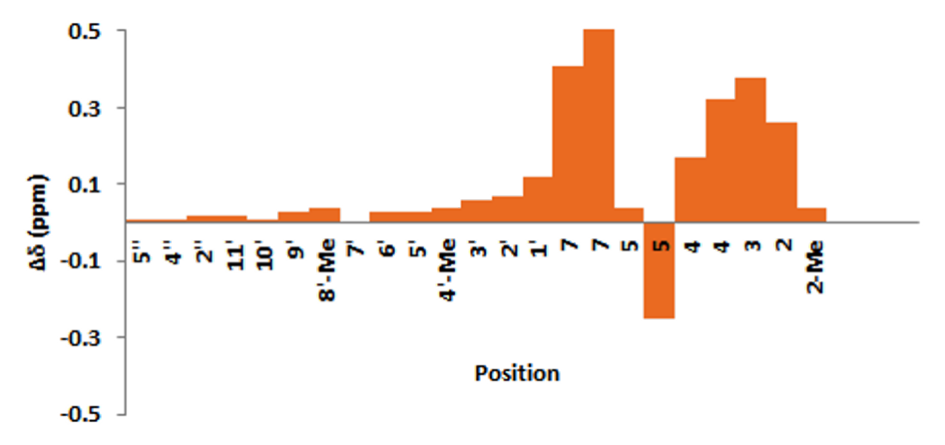
Δδ (ppm) of ^1^H NMR chemical shifts in **1**. Δδ corresponds to the difference in chemical shift for natural and synthetic products (Δδ = δ(synthetic) – δ(natural)).

**Figure 3:**
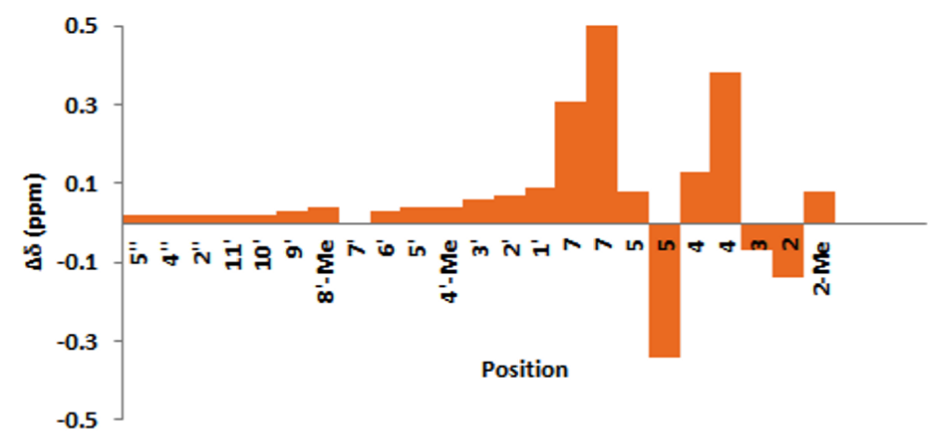
Δδ (ppm) of ^1^H NMR chemical shifts in **1’**. Δδ corresponds to the difference in chemical shift for natural and synthetic products (Δδ = δ(synthetic) – δ(natural)).

Finally, determination of the exact structure of astakolactin was attempted by comparing the ^1^H and ^13^C NMR spectra of the synthetic compounds **1** and **1’** with those reported for astakolactin (Figure 2 and Figure 3).

The chemical shifts for the terpene chain of the synthetic compound **1** in the ^1^H and ^13^C NMR spectra were quite similar to those in the spectra of the natural product (Figure 2; see also Supporting Information File 1). However, the chemical shifts corresponding to the lactone ring moiety (from C-1 to C-7) in the ^1^H and ^13^C NMR spectra were somewhat different from those in the corresponding spectra of the natural product. Similar discrepancies in the spectral data were also found for the diastereomer **1’** (Figure 3; see also Supporting Information File 1). These facts indicate that the proposed eight-membered lactone moiety is not comprised in the naturally occurring astakolactin. On the basis of these results, it was concluded that the structures of synthetic compounds **1** and **1’** and the natural product are very similar, but not completely identical.

## Conclusion

In conclusion, we have achieved the total synthesis of the proposed structures of astakolactin (**1**) and its stereoisomer (**1’**), with a Johnson–Claisen rearrangement, an asymmetric Mukaiyama aldol reaction, and our MNBA-mediated lactonization as key steps. It was found that the ^1^H and ^13^C NMR data of synthesized **1** and **1’** are not identical with those of the natural compound. Further studies to elucidate the complete structure of astakolactin are now in progress.

## Experimental

**General procedure for the synthesis of 1 using the MNBA-mediated lactonization.** To a solution of MNBA (14 mg, 0.04 mol) and DMAP (23 mg, 0.19 mmol) in dichloromethane (25 mL) at room temperature was slowly added a solution of the seco-acid **2** (13 mg, 0.03 mol) in dichloromethane (6.2 mL) with a mechanically driven syringe over a 12 h period. After cooling to 0 °C, saturated aqueous sodium hydrogen carbonate was added. The mixture was extracted with dichloromethane, and the organic layer was washed with brine and water and dried over sodium sulfate. After evaporation of the solvent, the crude product was purified by thin-layer chromatography on silica gel (hexane/ethyl acetate 2:1) to afford **1** (9 mg, 71%). IR (neat): 3455, 2931, 1735 cm^−1^; ^1^H NMR (CDCl_3_) δ 7.33 (s, 1H, 5’’-H), 7.21 (s, 1H, 2’’-H), 6.27 (s, 1H, 4’’-H), 5.33 (t, *J* = 7.5 Hz, 1H, 1’-H), 5.17 (t, *J* = 7.5 Hz, 1H, 9’-H), 5.10 (t, *J* = 7.5 Hz, 1H, 5’-H), 5.08 (d, *J* = 12.0 Hz, 1H, 7-H), 4.60 (d, *J* = 12.0 Hz, 1H, 7-H), 4.09–4.02 (m, 1H, 3-H), 2.96 (dq, *J* = 5.0, 7.0 Hz, 1H, 2-H), 2.45 (t, *J* = 7.0 Hz, 2H, 11’-H), 2.30 (ddd, *J* = 2.5, 8.5, 14.5 Hz, 1H, 5-H), 2.24 (dt, *J* = 7.0, 7.5 Hz, 2H, 10’-H), 2.12 (dt, *J* = 7.5, 7.5 Hz, 2H, 2’-H), 2.08 (dt, *J* = 7.5, 7.5 Hz, 2H, 6’-H), 2.01 (dd, *J* = 7.5, 14.5 Hz, 1H, 5-H), 2.01 (t, *J* = 7.5 Hz, 2H, 3’-H), 1.99 (t, *J* = 7.5 Hz, 2H, 7’-H), 1.94–1.85 (m, 1H, 4-H), 1.83 (d, *J* = 7.0 Hz, 1H, OH), 1.83–1.76 (m, 1H, 4-H), 1.59 (s, 6H, 4’-Me, 8’-Me), 1.22 (d, *J* = 7.0 Hz, 3H, 2-Me); ^13^C NMR (CDCl_3_) δ 176.6 (1), 142.5 (5’’), 138.8 (2’’), 136.2 (6), 135.7 (8’), 134.1 (4’), 130.5 (1’), 125.0 (3’’), 124.9 (5’), 123.8 (9’), 111.1 (4’’), 74.1 (3), 65.7 (7), 42.5 (2), 39.6 (7’), 39.4 (3’), 35.1 (4), 29.9 (5), 28.4 (10’), 26.6 (6’), 26.2 (2’), 25.0 (11’), 16.0 (4’-Me, 8’-Me), 11.6 (2-Me); HRMS: [M + Na]^+^ calcd for C_25_H_36_O_4_Na, 423.2506; found, 423.2488.

## Supporting Information

File 1 Experimental procedures, analyitical data, and copies of ^1^H and ^13^C NMR spectra of all new compounds.
