# Supplementary material for: Total synthesis of the proposed structure of astakolactin
Source: Beilstein J Org Chem. 2014 Oct 17;10:2421–7. doi: 10.3762/bjoc.10.252 (PMC4222296; doi:10.3762/bjoc.10.252)
Supplement: File 1 — Experimental procedures, analyitical data, and copies of 1H and 13C NMR spectra of all new compounds. [file Beilstein_J_Org_Chem-10-2421-s001.pdf]

Supporting Information
for
Total synthesis of the proposed structure of astakolactin

Takayuki Tonoi*, Keisuke Mameda, Moe Fujishiro, Yutaka Yoshinaga
and Isamu Shiina*

Address: Department of Applied Chemistry, Tokyo University of Science,
1-3 Kagurazaka, Shinjuku-ku, Tokyo 162-8601, Japan

Email: Takayuki Tonoi - tonoi@rs.tus.ac.jp; Isamu Shiina - shiina@rs.kagu.tus.ac.jp
*Corresponding author

Experimental procedures, analyitical data, and copies of ^{1}H and ^{13}C NMR spectra of all new compounds

Table of contents

1. General information: S1
2. Experimental procedures and analytical data: S2 to S11
3. Preparation of alcohol 13: S12 to S16
4. Supplementary Tables and Figures: S17 to S18
5. References: S18
6. Copies of ^{1}H and ^{13}C NMR spectra: S19 to S63

1. General information

Infrared (IR) spectra were obtained using a Horiba FT-300 Fourier transform infrared spectrometer. Proton and carbon nuclear magnetic resonance (^{1}H and ^{13}C NMR) spectra were recorded with chloroform (in $CDCl_3$) on the following instruments: JEOL JNM-AL500 (^{1}H at 500 MHz and ^{13}C at 125 MHz). Optical rotations were determined using a Jasco P-1020 polarimeter. Mass spectra were determined by a Bruker Daltonics micrOTOF focus (ESI-TOF) mass spectrometer. Thin layer chromatography was performed on Wakogel B5F. HPLC was performed with a Hitachi LaChrom Elite system composed of the Organizer, L-2400 UV Detector, and L-2130 Pump.
All reactions were carried out under argon atmosphere in dried glassware unless otherwise noted. Dichloromethane was distilled from diphosphorus pentoxide, then calcium hydride, and dried over MS 4 Å, benzene and toluene were distilled from diphosphorus pentoxide, and dried over MS 4 Å, and THF and diethyl ether were distilled from sodium/benzophenone immediately prior to use. All reagents were purchased from Tokyo Kasei Kogyo Co., Ltd., Kanto Chemical Co., Inc. or Aldrich Chemical Co., Inc., and used without further purification unless otherwise noted. MNBA was

purchased from Tokyo Kasei Kogyo Co. Ltd. (TCI M1439).[1]

2. Experimental procedures and analytical data

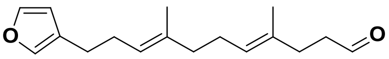

(4*E*,8*E*)-11-(Furan-3-yl)-4,8-dimethylundeca-4,8-dienal (6): To a cooled (-78 ºC) solution of oxalyl chloride (0.33 mL, 3.73 mmol) in 26 mL of dry dichloromethane was added DMSO (0.4 mL, 6.10 mmol) dropwise; the mixture was stirred at this temperature for 15 min. Then, a solution of alcohol **13** (889 mg, 3.39 mmol) in 8 mL of dry dichloromethane was added dropwise, and the resulting solution was stirred at -78 ºC for an additional 15 min. Finally, triethylamine (2.4 mL, 16.9 mmol) was added dropwise, and the mixture was stirred at 0 ºC for 40 min. The reaction was quenched by addition of water and, after a stirring vigorously, the layers were separated. The aqueous layer was extracted with dichloromethane. The combined organic layers were washed with water and brine, dried over anhydrous Na_2SO_4, and evaporated. The crude product was purified by column chromatography on silica gel (hexane/ethyl acetate = 5/1) to afford the aldehyde **6** (828 mg, 94%). IR (neat): 2916, 2715, 1728 cm^{-1}; ^{1}H NMR ($CDCl_3$): δ 9.74 (t, *J* = 2.5 Hz, 1H, 1-H), 7.34 (s, 1H, 5'-H), 7.21 (s, 1H, 2'-H), 6.27 (s, 1H, 4'-H), 5.15 (t, *J* = 7.5 Hz, 1H, 9-H), 5.12 (t, *J* = 7.5 Hz, 1H, 5-H), 2.50 (dt, *J* = 2.5, 7.5 Hz, 2H, 2-H), 2.45 (t, *J* = 7.5 Hz, 2H, 11-H), 2.31 (t, *J* = 7.5 Hz, 2H, 3-H), 2.24 (dt, *J* = 7.5, 7.5 Hz, 2H, 10-H), 2.08 (dt, *J* = 7.0, 7.5 Hz, 2H, 6-H), 1.98 (t, *J* = 7.0 Hz, 2H, 7-H), 1.61 (s, 3H), 1.58 (s, 3H, 4-Me); ^{13}C NMR ($CDCl_3$): δ 202.4 (1), 142.4 (5'), 138.7 (2'), 135.2 (8), 132.8 (4), 125.1 (5), 124.8 (3'), 123.9 (9), 110.9 (4'), 42.0 (2), 39.3 (6), 31.7 (3), 28.2 (10), 26.3 (6), 24.9 (11), 15.9 (8-Me), 15.8 (4-Me); HRMS: calcd for $C_{17}H_{24}O_2Na$ (M + Na^+) 283.1669, found 283.1655.

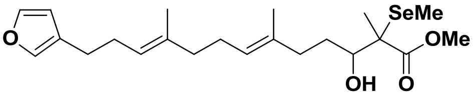

Methyl (6*E*,10*E*)-13-(furan-3-yl)-3-hydroxy-2,6,10-trimethyl-2-(methylseleno)trideca-6,10-dienoate (14): To a solution of diisopropylamine (0.09 mL, 0.61 mmol) in THF (3.0 mL) at 0 ºC was added butyllithium in hexane (1.54 M, 0.37 mL, 0.57 mmol), and then the reaction mixture was stirred for 15 min at that temperature. After the solution was cooled to -78 ºC, methyl 2-methylselenopropanoate (103 mg, 0.57 mmol) in THF (0.7 mL) was added. The mixture was stirred at -78 ºC for 30 min and the aldehyde **6** (123 mg, 0.47 mmol) in THF (1.0 mL) was added. After a stirring for 1 h, saturated aqueous ammonium chloride was added. The organic layer was separated and the aqueous layer was extracted with ethyl acetate. The combined organic layers were dried over anhydrous Na_2SO_4, and evaporated. The crude product was obtained and used in the next step without further purification.

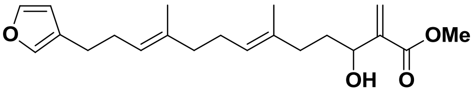

Methyl (6*E*,10*E*)-13-(furan-3-yl)-3-hydroxy-6,10-dimethyl-2-methylenetrideca-6,10-dienoate (5): To a solution of the seleno ester **14** in THF (2.4 mL) at 0 °C was added a 30% aqueous solution of hydrogen peroxide (0.1 mL). The mixture was stirred for 1 h at room temperature, and then quenched with saturated aqueous $Na_2S_2O_3$. The organic layer was separated and the aqueous layer was extracted with diethyl ether. The combined organic layer was washed with water and brine, and dried over Na_2SO_4. After evaporation of the solvent, the crude product was purified by preparative TLC on silica gel (hexane/ethyl acetate = 5/1) to afford the ester **5** (109 mg, 66%). IR (neat): 3448, 2924, 1720, 1628 cm^{-1}; ^1H NMR ($CDCl_3$): δ 7.34 (s, 1H, 5'-H), 7.21 (s, 1H, 2'-H), 6.28 (s, 1H, 4'-H), 6.24 (s, 1H, 2-CH_2), 5.81 (s, 1H, 2-CH_2), 5.18-5.14 (m, 2H, 7-H, 11-H), 4.40-4.36 (m, 1H, 3-H), 3.78 (s, 3H, OMe), 2.53 (d, J = 7.5 Hz, 1H, OH), 2.45 (t, J = 7.5 Hz, 2H, 13-H), 2.27-2.21 (m, 2H, 12-H), 2.18-2.05 (m, 4H, 5-H, 8-H), 2.00 (t, J = 7.5 Hz, 2H, 9-H), 1.82-1.75 (m, 1H, 4-H), 1.75-1.66 (m, 1H, 4-H), 1.62 (s, 3H, 10-Me), 1.59 (s, 3H, 6-Me); ^{13}C NMR ($CDCl_3$): δ 166.9 (1), 142.5 (5'), 142.4 (2), 138.8 (2'), 135.6 (10), 134.4 (6), 124.9 (3', 7, 2-CH_2), 123.8 (11), 111.0 (4), 71.2 (3), 51.8 (OMe), 39.5 (9), 35.8 (5), 34.4 (4), 28.4 (12), 26.4 (8), 25.0 (13), 16.0 (10-Me), 15.9 (6-Me); HR MS: calcd for $C_{21}H_{30}O_4Na$ (M + Na^+) 369.2036, found 369.2020.

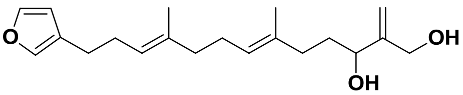

(6*E*,10*E*)-13-(Furan-3-yl)-6,10-dimethyl-2-methylenetrideca-6,10-diene-1,3-diol (15): To a solution of the ester **5** (637 mg, 1.84 mmol) in toluene (18.4 mL) at 0 °C was added 1.65 mL of Red-Al (65 wt% in toluene). The mixture was stirred for 30 min at that temperature, and then quenched with methanol and saturated potassium sodium tartrate (Rochelle salt) solution. The organic layer was separated and the aqueous layer was extracted with dichloromethane. The combined organic layer was washed with water and brine, and dried over Na_2SO_4. After evaporation of the solvent, the crude product was purified by preparative TLC on silica gel (hexane/ethyl acetate = 5/1) to afford the diol **15** (378 mg, 65%). IR (neat): 3348, 2924, 1658 cm^{-1}; ^1H NMR ($CDCl_3$): δ 7.34 (s, 1H, 5'-H), 7.21 (s, 1H, 2'-H), 6.28 (s, 1H, 4'-H), 5.23-5.15 (m, 2H, 11-H, 7-H), 5.14 (s, 1H, 2-CH_2), 5.10 (s, 1H, 2-CH_2), 4.31 (dd, J = 5.0, 13.5 Hz, 1H, 1-H), 4.24 (dt, J = 5.0, 6.0 Hz, 1H, 3-H), 4.17 (dd, J = 6.0, 13.5 Hz, 1H, 1-H), 2.45 (t, J = 7.0 Hz, 2H, 13-H), 2.24 (dt, J = 7.5, 7.0 Hz, 2H, 12-H), 2.09 (dt, J = 7.5, 7.5 Hz, 2H, 8-H), 2.06 (t, J = 7.5 Hz, 2H, 5-H), 2.00 (t, J = 7.5 Hz, 2H, 9-H), 1.93 (dd, J = 5.0, 6.0 Hz, 1H, 1-OH), 1.77-1.69 (m, 2H, 4-H), 1.62 (s, 3H, 10-Me), 1.59 (s, 3H, 6-Me); ^{13}C NMR ($CDCl_3$): δ 149.7 (2), 142.5 (5'), 138.8 (2'), 135.6 (10), 134.5 (6), 125.0 (3'), 124.8 (7), 123.8 (11), 112.4 (2-CH_2), 111.0 (4'), 74.4 (3), 63.9 (1), 39.5 (9), 35.8 (5), 33.8 (4), 28.4 (12), 26.4 (8), 25.0 (13), 16.0 (10-Me), 15.9 (6-Me); HR MS: calcd for $C_{20}H_{30}O_3Na$ (M + Na^+) 341.2087, found 341.2094.

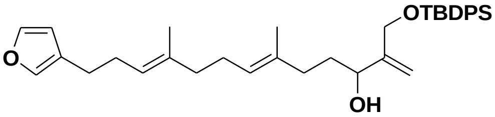

(6*E*,10*E*)-2-((*tert*-Butyldiphenylsiloxy)methyl)-13-(furan-3-yl)-6,10-dimethyl-trideca-1,6,10-trien-3-ol (16): To a solution of the diol **15** and imidazole (194 mg, 2.85 mmol) in DMF (11.9 mL) at 0 °C was added *tert*-butyldiphenylsilyl chloride (TBDPSCl) (0.37 mL, 1.43 mmol). After stirring for 40 min, the reaction mixture was quenched by addition of saturated aqueous NH_4Cl and extracted with diethyl ether. The organic layer was separated and dried over Na_2SO_4. After evaporation of the solvent, the crude product was purified by column chromatography on silica gel (hexane/ethyl acetate = 3/1) to afford the silyl ether **16** (656 mg, 99%). IR (neat): 3440, 2924, 1658 cm^{-1}; ^{1}H NMR ($CDCl_3$): δ 7.68 (d, *J* = 7.5 Hz, 4H, TBDPS), 7.43 (t, *J* = 7.5 Hz, 2H, TBDPS), 7.39 (dd, *J* = 7.5, 7.5 Hz, 4H, TBDPS), 7.33 (s, 1H, 5'-H), 7.20 (s, 1H, 2'-H), 6.27 (s, 1H, 4'-H), 5.17 (s, 1H, 1-H), 5.16 (t, *J* = 7.5 Hz, 11-H), 5.10 (s, 1H, 1-H), 5.10 (t, *J* = 7.5 Hz, 1H, 7-H), 4.30 (d, *J* = 13.5 Hz, 1H, 2-CH_2), 4.17 (d, *J* = 13.5 Hz, 1H, 2-CH_2), 4.15 (dt, *J* = 5.0, 6.0 Hz, 1H, 3-H), 2.44 (t, *J* = 7.5 Hz, 2H, 13-H), 2.23 (dt, *J* = 7.5, 7.5 Hz, 2H, 12-H), 2.21 (d, *J* = 5.0 Hz, 1H, 3-OH), 2.10-2.01 (m, 3H, 5-H, 8-H), 2.01-1.92 (m, 3H, 5-H, 9-H), 1.66 (ddd, *J* = 6.0, 7.5, 8.5 Hz, 2H, 4-H), 1.58 (s, 3H, 6-Me), 1.57 (s, 3H, 10-Me), 1.06 (s, 9H, TBDPS); ^{13}C NMR ($CDCl_3$): δ 149.4 (2), 142.5 (5'), 138.8 (2'), 135.7 (10), 135.5 (TBDPS), 134.6 (6), 133.1 (TBDPS), 129.8 (TBDPS), 127.7 (TBDPS), 125.0 (3'), 124.6 (7), 123.8 (11), 111.2 (1), 111.1 (4'), 73.7 (3), 64.9 (2-CH_2), 39.6 (9), 35.8 (5), 34.0 (4), 28.4 (12), 26.8 (TBDPS), 26.6 (8), 25.0 (13), 19.2 (TBDPS), 16.0 (10, 6); HR MS: calcd for $C_{36}H_{48}O_3SiNa$ (M + Na^+) 579.3265, found 579.3293.

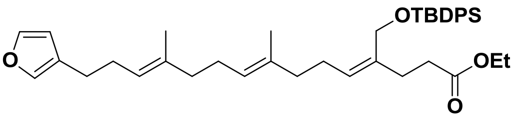

Ethyl (4*Z*,8*E*,12*E*)-4-((*tert*-butyldiphenylsiloxy)methyl)-15-(furan-3-yl)-8,12-dimethylpentadeca-4,8,12-trienoate (17): A mixture of the silyl ether **16** (22 mg, 40 μmol), triethyl orthoacetate (1.45 mL, 7.98 mmol), and propanoic acid (4.0 μg, 0.4 μmol) was stirred at 100 °C in a flask equipped with Dean–Stark trap for 12 h. After cooling to room temperature, the solvent was evaporated and the crude product was purified by preparative TLC on silica gel (hexane/ethyl acetate = 8/1) to afford the ester **17** (18 mg, 72%). IR (neat): 2931, 1736, 1666 cm^{-1}; ^{1}H NMR ($CDCl_3$): δ 7.68 (d, *J* = 7.5 Hz, 4H, TBDPS), 7.42 (t, *J* = 7.5 Hz, 2H, TBDPS), 7.38 (dd, *J* = 7.5, 7.5 Hz, 4H, TBDPS), 7.32 (s, 1H, 5'-H), 7.19 (s, 1H, 2'-H), 6.26 (s, 1H, 4'-H), 5.24-5.18 (m, 1H, 5-H), 5.15 (t, *J* = 7.5 Hz, 1H, 13-H), 5.00 (t, *J* = 7.5 Hz, 1H, 9-H), 4.19 (s, 2H, 4-CH_2), 4.12 (q, *J* = 7.5 Hz, 2H, OEt), 2.52 (t, *J* = 8.5 Hz, 2H, 3-H), 2.46 (t, *J* = 8.5 Hz, 2H, 2-H), 2.43 (t, *J* = 7.0 Hz, 2H, 15-H), 2.23 (dt, *J* = 7.0, 7.5 Hz, 2H, 14-H), 2.02 (dt, *J* = 7.5, 7.5 Hz, 2H, 10-H), 1.95 (t, *J* = 7.5 Hz, 2H, 11-H), 1.89-1.85 (m, 4H, 6-H, 7-H), 1.57 (s, 3H, 12-Me), 1.47 (s, 3H, 8-Me), 1.24 (t, *J* = 7.5 Hz, 3H, OEt), 1.04 (s, 9H, TBDPS); ^{13}C NMR ($CDCl_3$): δ 173.5 (1), 142.5 (5'), 138.8 (2'), 136.6 (4), 135.7 (12), 135.6 (TBDPS), 134.4 (8), 133.7 (TBDPS), 129.6 (TBDPS), 127.6 (TBDPS), 127.1 (5), 125.0 (3'), 124.5 (9), 123.7 (13), 111.1 (4'), 61.2 (4-CH_2), 60.1 (OEt), 39.6 (7, 11), 33.6 (2), 30.1 (3), 28.4 (14), 26.8 (TBDPS), 26.6 (10), 26.0 (6), 25.0 (15), 19.2 (TBDPS), 16.0 (12-Me), 15.9 (8-Me), 14.3 (OEt); HR MS: calcd for $C_{40}H_{54}O_4SiNa$ (M + Na^+) 649.3684, found 649.3678.

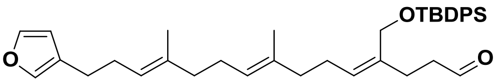

(4*Z*,8*E*,12*E*)-4-((*tert*-Butyldiphenylsiloxy)methyl)-15-(furan-3-yl)-8,12-dimethylpentadeca-4,8,12-trienal (18): To a solution of the ester **17** (22 mg, 35 μmol) in hexane (0.35 mL) at -78 °C was added 34 μL of DIBAL-H (1.03 M in hexane). The mixture was stirred for 25 min at that temperature, and then quenched with methanol and saturated potassium sodium tartrate (Rochelle salt) solution. The organic layer was separated and the aqueous layer was extracted with hexane. The combined organic layer was dried over Na_2SO_4. After evaporation of the solvent, the crude product was purified by preparative TLC on silica gel (hexane/ethyl acetate = 10/1, twice) to afford the aldehyde **18** (15 mg, 72%). IR (neat): 2931, 2715, 1728 cm^{-1}; ^1H NMR ($CDCl_3$): δ 9.75 (s, 1H, 1-H), 7.67 (d, *J* = 6.0 Hz, 4H, TBDPS), 7.43 (t, *J* = 7.5 Hz, 2H, TBDPS), 7.38 (dd, *J* = 6.0, 7.5 Hz, 4H, TBDPS), 7.33 (s, 1H, 5'-H), 7.20 (s, 1H, 2'-H), 6.27 (s, 1H, 4'-H), 5.24-5.17 (m, 1H, 5-H), 5.15 (t, *J* = 7.5 Hz, 1H, 13-H), 5.00 (t, *J* = 7.5 Hz, 1H, 9-H), 4.20 (s, 2H, 4-CH_2), 2.58-2.50 (m, 4H, 2-H, 3-H), 2.44 (t, *J* = 7.5 Hz, 2H, 15-H), 2.23 (dt, *J* = 7.5, 7.5 Hz, 2H, 14-H), 2.03 (dt, *J* = 7.5, 7.5 Hz, 2H, 10-H), 1.95 (t, *J* = 7.5 Hz, 2H, 11-H), 1.90-1.86 (m, 4H, 6-H, 7-H), 1.58 (s, 3H, 12-H), 1.47 (s, 3H, 8-H), 1.04 (s, 9H, TBDPS); ^{13}C NMR ($CDCl_3$): δ 202.8 (1), 142.5 (5'), 138.8 (2'), 136.2 (4), 135.7 (12), 135.6 (TBDPS), 134.2 (8), 133.5 (TBDPS), 129.7 (TBDPS), 127.7 (TBDPS), 127.4 (5), 124.9 (3'), 124.6 (9), 123.7 (13), 111.1 (4'), 61.2 (4-CH_2), 42.6 (2), 39.6 (11), 39.5 (7), 28.4 (14), 27.3 (3), 26.8 (TBDPS), 26.6 (14), 25.9 (6), 25.0 (15), 19.2 (TBDPS), 16.0 (12), 15.8 (8); HR MS: calcd for $C_{38}H_{50}O_3SiNa$ ($M + Na^+$) 605.3421, found 605.3450.

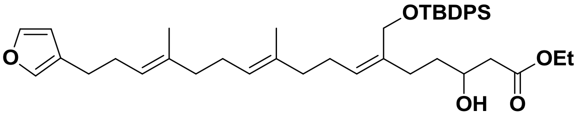

Ethyl (6*Z*,10*E*,14*E*)-6-((*tert*-butyldiphenylsiloxy)methyl)-17-(furan-3-yl)-3-hydroxy-10,14-dimethylheptadeca-6,10,14-trienoate (19): To a solution of diisopropylamine (43 μL, 0.30 mmol) in THF (1.8 mL) at 0 ºC was added butyllithium in hexane (1.57 M, 0.19 mL, 0.29 mmol), and then the reaction mixture was stirred for 20 min at that temperature. After the solution was cooled to -78 ^oC, ethyl acetate (27 μL, 0.28 mmol) was added. The mixture was stirred at -78 ºC for 30 min and the aldehyde **18** (81 mg, 0.14 mmol) in THF (1.0 mL) was added. After a stirring for 80 min, saturated aqueous ammonium chloride was added. The organic layer was separated and the aqueous layer was extracted with ethyl acetate. The combined organic layers were dried over anhydrous Na_2SO_4. After evaporation of the solvent, the crude product was purified by preparative TLC on silica gel (hexane/ethyl acetate = 7/1, twice) to afford the aldol product **19** (77 mg, 84%). IR (neat): 3579, 2931, 1728 cm^{-1}; ^1H NMR ($CDCl_3$): δ 7.68 (d, *J* = 7.0 Hz, 4H, TBDPS), 7.42 (t, *J* = 7.5 Hz, 2H, TBDPS), 7.38 (dd, *J* = 7.0, 7.5 Hz, 4H, TBDPS), 7.33 (s, 1H, 5'-H), 7.20 (s, 1H, 2'-H), 6.26 (s, 1H, 4'-H), 5.25-5.20 (m, 1H, 7-H), 5.15 (t, *J* = 7.5 Hz, 1H, 15-H), 5.01 (t, *J* = 7.5 Hz, 1H, 11-H), 4.18 (s, 2H,

6-CH_2), 4.17 (q, *J* = 7.5 Hz, 2H, OEt), 4.04-3.96 (m, 1H, 3-H), 2.90 (d, *J* = 5.0 Hz, 1H, OH), 2.47-2.38 (m, 2H, 2-H), 2.44 (t, *J* = 7.5 Hz, 2H, 17-H), 2.36-2.25 (m, 2H, 5-H), 2.23 (dt, *J* = 7.5, 7.5 Hz, 2H, 16-H), 2.03 (dt, *J* = 7.5, 7.5 Hz, 2H, 12-H), 1.95 (t, *J* = 7.5 Hz, 2H, 13-H), 1.92-1.86 (m, 4H, 8-H, 9-H), 1.69-1.58 (m, 2H, 4-H), 1.58 (s, 3H, 14-Me), 1.48 (s, 3H, 10-Me), 1.27 (t, *J* = 7.5 Hz, 3H, OEt), 1.04 (s, 9H, TBDPS); ^{13}C NMR ($CDCl_3$): δ 172.9 (1), 142.5 (5'), 138.8 (2'), 137.4 (6), 135.7 (14), 135.6 (TBDPS), 134.4 (10), 133.7 (TBDPS), 129.6 (TBDPS), 127.6 (TBDPS), 127.1 (7), 124.9 (3'), 124.5 (11), 123.7 (15), 111.1 (4'), 67.9 (3), 61.2 (6-CH_2), 60.6 (OEt), 41.4 (2), 39.7 (13), 39.6 (9), 35.1 (4), 30.7 (5), 28.4 (16), 26.8 (TBDPS), 26.6 (12), 26.0 (8), 25.0 (17), 19.2 (TBDPS), 16.0 (14-Me), 15.9 (10-Me), 14.2 (OEt); HR MS: calcd for $C_{42}H_{58}O_5SiNa$ (M + Na^+) 693.3946, found 693.3921.

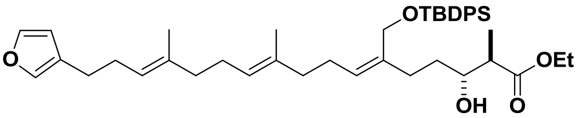

Ethyl (2*R,3*R**,6*Z*,10*E*,14*E*)-6-((*tert*-butyldiphenylsiloxy)methyl)-17-(furan-3-yl)-3-hydroxy-2,10,14-trimethylheptadeca-6,10,14-trienoate (20):** To a solution of the aldol product **19** (77 mg, 0.12 mmol) in THF (2.6 mL) at -78 °C was added 0.46 mL of LHMDS (1.0 M in THF). After the mixture was stirred for 1 h at -45 °C, methyl iodide (81 μL, 1.3 mmol) was added. After stirring for 4 h, saturated aqueous ammonium chloride was added. The organic layer was separated and the aqueous layer was extracted with ethyl acetate. The combined organic layers were dried over anhydrous Na_2SO_4. After evaporation of the solvent, the crude product was purified by preparative TLC on silica gel (hexane/ethyl acetate = 5/1, twice) to afford the ester **20** (31 mg, 40%) and substrate recovered (33 mg, 43%). IR (neat): 3510, 2962, 1728 cm^{-1}; ^{1}H NMR ($CDCl_3$): δ 7.68 (d, *J* = 6.0 Hz, 4H, TBDPS), 7.42 (t, *J* = 7.5 Hz, 2H, TBDPS), 7.38 (dd, *J* = 6.0, 7.5 Hz, 4H, TBDPS), 7.33 (s, 1H, 5'-H), 7.19 (s, 1H, 2'-H), 6.26 (s, 1H, 4'-H), 5.23 (t, *J* = 6.0 Hz, 1H, 7-H), 5.15 (t, *J* = 7.5 Hz, 1H, 15-H), 5.01 (t, *J* = 7.5 Hz, 1H, 11-H), 4.21 (d, *J* = 12.0 Hz, 1H, 6-CH_2), 4.16 (q, *J* = 7.5 Hz, 2H, OEt), 4.15 (d, *J* = 12.0 Hz, 1H, 6-CH_2), 3.69-3.62 (m, 1H, 3-H), 2.60 (d, *J* = 7.5 Hz, 1H, OH), 2.50 (dq, *J* = 7.0, 7.5 Hz, 1H, 2-H), 2.43 (t, *J* = 7.0 Hz, 2H, 17-H), 2.40-2.25 (m, 2H, 5-H), 2.23 (dt, *J* = 7.0, 7.5 Hz, 2H, 16-H), 2.03 (dt, *J* = 7.5, 7.5 Hz, 2H, 12-H), 1.95 (t, *J* = 7.5 Hz, 2H, 13-H), 1.92-1.87 (m, 4H, 8-H, 9-H), 1.73-1.64 (m, 1H, 4-H), 1.60-1.51 (m, 1H, 4-H), 1.58 (s, 3H, 14-Me), 1.48 (s, 3H, 9-Me), 1.26 (t, *J* = 7.5 Hz, 3H, OEt), 1.19 (d, *J* = 7.5 Hz, 3H, 2-Me), 1.04 (s, 9H, TBDPS); ^{13}C NMR ($CDCl_3$): δ 176.0 (1), 142.5 (5'), 138.8 (2'), 137.6 (6), 135.7 (14), 135.6 (TBDPS), 134.5 (10), 133.7 (TBDPS), 129.6 (TBDPS), 127.6 (TBDPS), 127.1 (7), 124.9 (3'), 124.4 (11), 123.7 (15), 111.1 (4'), 73.2 (3), 61.3 (6-CH_2), 60.5 (OEt), 45.3 (2), 39.7 (13), 39.6 (9), 33.4 (4), 30.8 (5), 28.4 (16), 26.8 (TBDPS), 26.6 (12), 26.0 (8), 25.0 (17), 19.2 (TBDPS), 16.0 (14-Me), 15.9 (10-Me), 14.3 (2-Me), 14.2 (OEt); HR MS: calcd for $C_{43}H_{60}O_5SiNa$ (M + Na^+) 707.4102, found 707.4126.

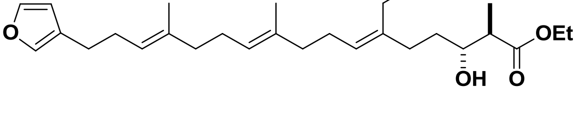

Ethyl (2*R,3*R**,6*Z*,10*E*,14*E*)-17-(furan-3-yl)-3-hydroxy-6-(hydroxymethyl)-2,10,14-trimethylheptadeca-6,10,14-trienoate (21):** To a solution of the ester **20** in THF/pyridine (= 3/2 v/v ratio, 1.9 mL) at 0 °C was added HF/pyridine solution (0.38 mL). The mixture was stirred for 13 h at room temperature, and then quenched with saturated aqueous $NaHCO_3$ at 0 °C. The organic layer was separated and the aqueous layer was extracted with ethyl acetate. The combined organic layer was washed with water and brine, and dried over Na_2SO_4. After evaporation of the solvent, the crude product was purified by preparative TLC on silica gel (hexane/ethyl acetate = 1/1) to afford the ester **21** (28 mg, 90%). IR (neat): 3432, 2931, 1712 cm^{-1}; ^{1}H NMR ($CDCl_3$): δ 7.33 (s, 1H, 5'-H), 7.21 (s, 1H, 2'-H), 6.27 (s, 1H, 4'-H), 5.33 (t, *J* = 7.5 Hz, 1H, 7-H), 5.16 (t, *J* = 7.5 Hz, 1H, 15-H), 5.10 (t, *J* = 7.5 Hz, 1H, 11-H), 4.17 (q, *J* = 7.0 Hz, 2H, OEt), 4.17 (d, *J* = 12.0 Hz, 1H, 6-CH_2), 4.10 (d, *J* = 12.0 Hz, 1H, 6-CH_2), 3.68 (ddd, *J* = 3.5, 6.0, 10.0 Hz, 1H, 3-H), 2.52 (dq, *J* = 6.0, 7.5 Hz, 1H, 2-H), 2.45 (t, *J* = 7.5 Hz, 2H, 17-H), 2.35-2.20 (m, 2H, 5-H), 2.24 (dt, *J* = 7.5, 7.5 Hz, 2H, 16-H), 2.17 (dt, *J* = 7.5, 7.5 Hz, 2H), 2.07 (dt, *J* = 7.5, 7.5 Hz, 2H), 2.00 (t, *J* = 7.5 Hz, 2H, 8-H), 1.99 (t, *J* = 7.5 Hz, 2H, 13-H), 1.73-1.65 (m, 2H, 4-H), 1.60 (s, 3H, 14-Me), 1.59 (s, 3H, 10-Me), 1.27 (t, *J* = 7.0 Hz, 3H, OEt), 1.20 (d, *J* = 7.5 Hz, 3H, 2-Me); ^{13}C NMR ($CDCl_3$): δ 175.9 (1), 142.5 (5'), 138.8 (2'), 138.0 (6), 135.6 (14), 134.5 (10), 128.8 (7), 124.9 (3'), 124.8 (11), 123.8 (15), 111.1 (4'), 72.9 (3), 60.6 (OEt), 60.3 (6-CH_2), 45.3 (2), 39.8 (13), 39.6 (9), 33.3 (4), 31.1 (5), 28.4 (16), 26.5 (12), 26.2 (8), 25.0 (17), 16.0 (8), 14.2 (OEt); HR MS: calcd for $C_{27}H_{42}O_5Na$ ($M + Na^+$) 469.2924, found 469.2922.

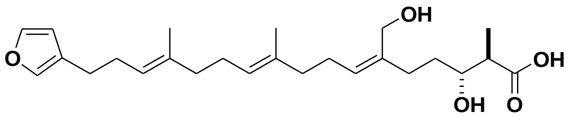

(2*R,3*R**,6*Z*,10*E*,14*E*)-17-(Furan-3-yl)-3-hydroxy-6-(hydroxymethyl)-2,10,14-trimethylheptadeca-6,10,14-trienoic acid (2):** To a solution of the ester **21** (28 mg, 62 μmol) in ethanol (0.83 mL) at 0 °C was added a 17 M aqueous solution of KOH (0.42 mL). The mixture was stirred for 1 h at room temperature, and then quenched with a 1 M aqueous solution of HCl (pH = 5-6). The mixture was extracted with diethyl ether and dried over Na_2SO_4. After evaporation of the solvent, the crude product was purified by preparative TLC on silica gel (chloroform/methanol/formic acid = 9/1/1) to afford the seco-acid **2** (25 mg, 97%). IR (neat): 3464, 2924, 1712 cm^{-1}; ^{1}H NMR ($CDCl_3$): δ 7.33 (s, 1H, 5'-H), 7.21 (s, 1H, 2'-H), 6.27 (s, 1H, 4'-H), 5.36 (t, *J* = 7.5 Hz, 1H, 7-H), 5.16 (t, *J* = 7.5 Hz, 1H, 15-H), 5.10 (t, *J* = 7.5 Hz, 1H, 11-H), 4.23 (d, *J* = 11.0 Hz, 1H, 6-CH_2), 4.12 (d, *J* = 11.0 Hz, 1H, 6-CH_2), 3.73 (ddd, *J* = 2.5, 6.0, 9.5 Hz, 1H, 3-H), 2.55 (dq, *J* = 6.0, 7.0 Hz, 2-H), 2.45 (t, *J* = 7.5 Hz, 2H, 17-H), 2.34-2.21 (m, 2H, 5-H), 2.24 (dt, *J* = 7.5, 7.5 Hz, 2H, 16-H), 2.16 (dt, *J* = 7.5, 7.5 Hz, 2H, 8-H), 2.07 (dt, *J* = 7.5, 7.5 Hz, 2H, 12-H), 2.00 (t, *J* = 7.5 Hz, 2H, 9-H), 1.99 (t, *J* = 7.5 Hz, 2H, 13-H), 1.80-1.72 (m, 1H, 4-H), 1.70-1.61 (m, 1H, 4-H), 1.59 (s, 6H, 10-Me, 14-Me), 1.24 (d, *J* = 7.0 Hz, 3H, 2-Me); ^{13}C NMR ($CDCl_3$): δ 179.2 (1), 142.5 (5'), 138.8 (2'), 137.4 (6), 135.7 (14), 134.4 (10), 129.1 (7), 125.0 (3'), 124.8 (11), 123.8 (15), 111.1 (4'), 72.9 (3), 60.2 (6-CH_2), 45.4 (2), 39.7 (13), 39.6 (9), 33.3 (4), 30.8 (5), 28.4 (16), 26.6 (12), 26.2 (8), 25.0 (17), 16.0 (10-Me, 14-Me), 14.0 (2-Me);

HR MS: calcd for $C_{25}H_{38}O_5Na$ (M + Na^+) 441.2611, found 441.2632.

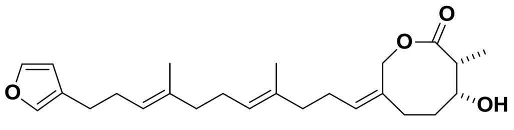

(2*R,3*R**,6*Z*)-6-((4*E*,8*E*)-11-(Furan-3-yl)-4,8-dimethylundeca-4,8-dien-1-ylidene)-3-hydroxy-2-methylheptan-7-olide (proposed structure of astakolaktin) (1): General procedure for the synthesis of 1 using MNBA-mediated lactonization.** To a solution of MNBA (14 mg, 0.04 mol) and DMAP (23 mg, 0.19 mmol) in dichloromethane (25 mL) at room temperature was slowly added a solution of the seco-acid **2** (13 mg, 0.03 mol) in dichloromethane (6.2 mL) with a mechanically driven syringe over a 12 h period. After cooling to 0 °C, saturated aqueous sodium hydrogencarbonate was added. The mixture was extracted with dichloromethane, and the organic layer was washed with brine and water, dried over sodium sulfate. After evaporation of the solvent, the crude product was purified by thin layer chromatography on silica gel (hexane/ethyl acetate = 2/1) to afford **1** (9 mg, 71%).

General procedure for the synthesis of 1 using Yamaguchi lactonization. To a solution of **2** (5.5 mg, 13 μmol) and Et_3N (2 μL, 14 μmol) in THF (0.5 mL) was added 2,4,6-trichlorobenzoyl chloride (3.2 mg, 13 μmol) in THF (0.5 mL) at room temperature. After stirring for 2 h, the mixture was added to a solution of DMAP (9.5 mg, 78 μmol) in dichloromethane (4.0 mL) with a mechanically driven syringe over a 12 h period. After cooling to 0 °C, saturated aqueous sodium hydrogencarbonate was added. The mixture was extracted with dichloromethane, and the organic layer was washed with brine and water, dried over sodium sulfate. After evaporation of the solvent, the crude product was purified by thin layer chromatography on silica gel (hexane/ethyl acetate = 2/1) to afford **1** (1.7 mg, 33%). IR (neat): 3455, 2931, 1735 cm^{-1}; ^1H NMR ($CDCl_3$): δ 7.33 (s, 1H, 5”-H), 7.21 (s, 1H, 2”-H), 6.27 (s, 1H, 4”-H), 5.33 (t, *J* = 7.5 Hz, 1H, 1’-H), 5.17 (t, *J* = 7.5 Hz, 1H, 9’-H), 5.10 (t, *J* = 7.5 Hz, 1H, 5’-H), 5.08 (d, *J* = 12.0 Hz, 1H, 7-H), 4.60 (d, *J* = 12.0 Hz, 1H, 7-H), 4.09-4.02 (m, 1H, 3-H), 2.96 (dq, *J* = 5.0, 7.0 Hz, 1H, 2-H), 2.45 (t, *J* = 7.0 Hz, 2H, 11’-H), 2.30 (ddd, *J* = 2.5, 8.5, 14.5 Hz, 1H, 5-H), 2.24 (dt, *J* = 7.0, 7.5 Hz, 2H, 10’-H), 2.12 (dt, *J* = 7.5, 7.5 Hz, 2H, 2’-H), 2.08 (dt, *J* = 7.5, 7.5 Hz, 2H, 6’-H), 2.01 (dd, *J* = 7.5, 14.5 Hz, 1H, 5-H), 2.01 (t, *J* = 7.5 Hz, 2H, 3’-H), 1.99 (t, *J* = 7.5 Hz, 2H, 7’-H), 1.94-1.85 (m, 1H, 4-H), 1.83 (d, *J* = 7.0 Hz, 1H, OH), 1.83-1.76 (m, 1H, 4-H), 1.59 (s, 6H, 4’-Me, 8’-Me), 1.22 (d, *J* = 7.0 Hz, 3H, 2-Me); ^{13}C NMR ($CDCl_3$): δ 176.6 (1), 142.5 (5”), 138.8 (2”), 136.2 (6), 135.7 (8’), 134.1 (4’), 130.5 (1’), 125.0 (3”), 124.9 (5’), 123.8 (9’), 111.1 (4”), 74.1 (3), 65.7 (7), 42.5 (2), 39.6 (7’), 39.4 (3’), 35.1 (4), 29.9 (5), 28.4 (10’), 26.6 (6’), 26.2 (2’), 25.0 (11’), 16.0 (4’-Me, 8’-Me), 11.6 (2-Me); HR MS: calcd for $C_{25}H_{36}O_4Na$ (M + Na^+) 423.2506, found 423.2488.

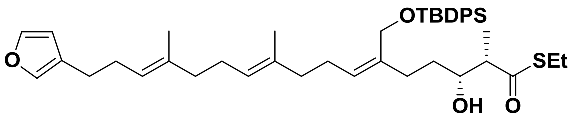

***S*-Ethyl (2*S*,3*R*,6*Z*,10*E*,14*E*)-6-((*tert*-butyldiphenylsiloxy)methyl)-17-(furan-3-yl)-3-hydroxy-2,10,14-trimethylheptadeca-6,10,14-trienethioate (24):** To a solution of $Sn(OTf)_2$ (70 mg, 0.17 mmol) in dichloromethane (0.6 mL) were added solutions of (*S*)-1-methyl-2-(1-naphthylaminomethyl)pyrrolidine (49 mg, 0.20 mmol) in dichloromethane (0.2 mL) and $^{n}Bu_2Sn(OAc)_2$ (65 mg, 0.19 mmol) in dichloromethane (0.1 mL), respectively. The mixture was cooled to −78 °C. To the reaction mixture were added solutions of KSA (26 mg, 0.14 mmol) in dichloromethane (0.2 mL) and the aldehyde **18** (47 mg, 81 μmol) in dichloromethane (0.52 mL) at −78 °C, successively. The mixture was stirred for 2 h at that temperature, and then quenched with saturated aqueous sodium hydrogencarbonate. The organic layer was separated and the aqueous layer was extracted with dichloromethane. The combined organic layer was washed with water and brine, and dried over Na_2SO_4. After evaporation of the solvent, the crude product was purified by preparative TLC on silica gel (hexane/ethyl acetate = 5/1) to afford the aldol product **24** (51 mg, 90%, 83% ee, dr = 93/7). HPLC analysis: DAICEL CHIRALPAK AD-H, UV 254 nm, temperature 25 °C, hexane/^{i}PrOH = 99/1, flow rate 0.3 mL/min, t_R (*syn*) = 58.0 min, 61.1 min; t_R (*anti*) = 74.3 min, 79.4 min; *syn*/*anti* = 93/7, 90% ee (*syn*). $[\alpha]_D^{27}$ +12.3 (*c* 1.27, $CHCl_3$); IR (neat): 3525, 2931, 1674 cm^{-1}; ^{1}H NMR ($CDCl_3$): δ 7.68 (d, *J* = 7.5 Hz, 4H, TBDPS), 7.42 (t, *J* = 7.5 Hz, 2H), 7.38 (dd, *J* = 7.5, 7.5 Hz, 4H, TBDPS), 7.33 (s, 1H, 5'-H), 7.20 (s, 1H, 2'-H), 6.27 (s, 1H, 4'-H), 5.23 (t, *J* = 7.0 Hz, 1H, 7-H), 5.15 (t, *J* = 7.5 Hz, 1H, 15-H), 5.01 (t, *J* = 7.0 Hz, 1H, 11-H), 4.20 (d, *J* = 12.0 Hz, 6-CH_2), 4.16 (d, *J* = 12.0 Hz, 1H, 6-CH_2), 3.90 (ddt, *J* = 3.5, 4.0, 8.5 Hz, 1H, 3-H), 2.88 (q, *J* = 7.0 Hz, 2H, SEt), 2.66 (dq, *J* = 3.5, 7.5 Hz, 1H, 2-H), 2.44 (t, *J* = 7.5 Hz, 2H, 17-H), 2.41 (d, *J* = 4.0 Hz, 1H, OH), 2.37-2.30 (m, 2H, 5-H), 2.23 (dt, *J* = 7.5, 7.5 Hz, 2H, 16-H), 2.03 (dt, *J* = 7.0, 7.5 Hz, 2H, 12-H), 1.95 (t, *J* = 7.5 Hz, 2H, 13-H), 1.93-1.87 (m, 4H, 15-H, 8-H), 1.64-1.52 (m, 2H, 4-H), 1.58 (s, 3H, 4-Me), 1.48 (s, 3H, 10-Me), 1.25 (t, *J* = 7.0 Hz, 3H, SEt), 1.20 (d, *J* = 7.5 Hz, 3H, 2-Me), 1.04 (s, 9H, TBDPS); ^{13}C NMR ($CDCl_3$): δ 204.0 (1), 142.5 (5'), 138.8 (2'), 137.4 (6), 135.7 (14), 135.6 (TBDPS), 134.4 (10), 133.6 (TBDPS), 129.6 (TBDPS), 127.6 (TBDPS), 127.2 (11), 124.9 (3'), 124.5 (11), 123.7 (15), 111.1 (4'), 71.8 (3), 61.2 (6-CH_2), 53.1 (2), 39.7 (13), 39.6 (9), 32.7 (4), 31.1 (5), 28.4 (16), 26.8 (TBDPS), 26.6 (12), 26.0 (8), 25.0 (17), 23.2 (SEt), 19.2 (TBDPS), 16.0 (14-Me), 15.9 (10-Me), 14.6 (SEt), 11.5 (2-Me); HR MS: calcd for $C_{43}H_{60}O_4SSiNa$ ($M + Na^+$) 723.3874, found 723.3843.

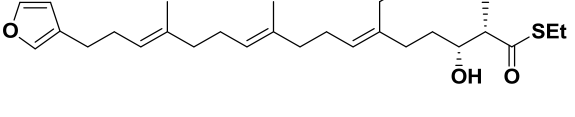

***S*-Ethyl (2*S*,3*R*,6*Z*,10*E*,14*E*)-17-(furan-3-yl)-3-hydroxy-6-(hydroxymethyl)-2,10,14-trimethylheptadeca-6,10,14-trienethioate (25):** To a solution of the aldol product **24** in THF/pyridine (= 3/2 v/v ratio, 2.1 mL) at 0 °C was added HF/pyridine solution (0.41 mL). The mixture was stirred for 12 h at room temperature, and then quenched with saturated aqueous sodium hydrogencarbonate at 0 °C. The organic layer was separated and the aqueous layer was extracted with ethyl acetate. The combined organic layer was washed with water and brine, and dried over Na_2SO_4. After evaporation of the solvent, the crude product was purified by preparative TLC on silica gel

(hexane/ethyl acetate = 1/1) to afford the diol **25** (32 mg, 94%). $[\alpha]_D^{27}$ +13.7 (*c* 0.69, $CHCl_3$); IR (neat): 3425, 2924, 1674 cm^{-1}; ^1H NMR ($CDCl_3$): δ 7.34 (s, 1H, 5'-H), 7.21 (s, 1H, 2'-H), 6.28 (s, 1H, 4'-H), 5.34 (t, *J* = 7.0 Hz, 1H, 7-H), 5.17 (t, *J* = 7.5 Hz, 1H, 15-H), 5.10 (t, *J* = 7.5 Hz, 1H, 11-H), 4.17 (d, *J* = 12.0 Hz, 1H, 6-CH_2), 4.09 (d, *J* = 12.0 Hz, 1H, 6-CH_2), 3.91 (ddd, *J* = 3.5, 4.0, 8.5 Hz, 1H, 3-H), 2.88 (q, *J* = 7.5 Hz, 2H, SEt), 2.68 (dq, *J* = 3.5, 7.5 Hz, 1H, 2-H), 2.57 (br s, 1H, 3-OH), 2.45 (t, *J* = 7.0 Hz, 2H, 17-H), 2.32-2.19 (m, 2H, 5-H), 2.24 (dt, *J* = 7.0, 7.5 Hz, 2H, 16-H), 2.17 (dt, *J* = 7.0, 7.5 Hz, 2H, 8-H), 2.07 (dt, *J* = 7.5, 7.5 Hz, 2H, 12-H), 2.00 (t, *J* = 7.5 Hz, 2H, 9-H), 1.99 (t, *J* = 7.5 Hz, 2H, 13-H), 1.70-1.57 (m, 2H, 4-H), 1.59 (s, 6H, 10-Me, 14-Me), 1.26 (t, *J* = 7.5 Hz, 3H, SEt), 1.22 (d, *J* = 7.5 Hz, 3H, 2-Me); ^{13}C NMR ($CDCl_3$): δ 204.1 (1), 142.5 (5'), 138.8 (2'), 137.8 (6), 135.6 (14), 134.4 (10), 129.0 (7), 124.9 (3'), 124.8 (11), 123.8 (15), 111.1 (4'), 71.7 (3), 60.2 (6-CH_2), 53.3 (2), 39.7 (13), 39.6 (9), 32.9 (4), 31.6 (5), 28.4 (16), 26.5 (12), 26.2 (8), 25.0 (17), 23.2 (SEt), 16.0 (10-Me, 14-Me), 14.6 (SEt), 11.8 (2-Me); HR MS: calcd for $C_{27}H_{42}O_4SNa$ (M + Na^+) 485.2696, found 485.2712.

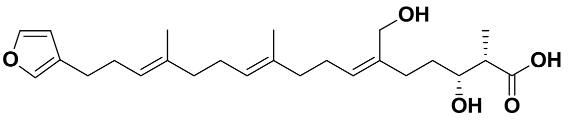

(2*S*,3*R*,6*Z*,10*E*,14*E*)-17-(Furan-3-yl)-3-hydroxy-6-(hydroxymethyl)-2,10,14-trimethylheptadeca-6,10,14-trienoic acid (26): To a solution of the diol **25** (28 mg, 61 mmol) in THF (0.3 mL) and water (1.2 mL) at room temperature was added lithium hydroxide (6 mg, 0.25 mmol) and 30% hydrogen peroxide in water (48 μL). After the reaction mixture was stirred for 45 min at room temperature, saturated aqueous ammonium chloride was added. The acidified mixture (pH = 5-6) was extracted with diethyl ether and the organic layer was washed with brine, dried over sodium sulfate. The crude product was purified by column chromatography on silica gel (chloroform/methanol/formic acid = 90/10/1) to afford the seco-acid **26** (22 mg, 87%). $[\alpha]_D^{27}$ +4.26 (*c* 0.74, $CHCl_3$); IR (neat): 3433, 2924, 1697 cm^{-1}; ^1H NMR ($CDCl_3$): δ 7.33 (s, 1H, 5'-H), 7.21 (s, 1H, 2'-H), 6.27 (s, 1H, 4'-H), 5.37 (t, *J* = 7.5 Hz, 1H, 7-H), 5.16 (t, *J* = 7.5 Hz, 1H, 15-H), 5.10 (t, *J* = 7.5 Hz, 1H, 11-H), 4.22 (d, *J* = 12.0 Hz, 1H, 6-CH_2), 4.10 (d, *J* = 12.0 Hz, 1H, 6-CH_2), 3.94 (ddd, *J* = 3.5, 4.0, 8.5 Hz, 1H, 3-H), 2.61 (dq, *J* = 3.5, 7.5 Hz, 1H, 2-H), 2.45 (t, *J* = 7.5 Hz, 2H, 17-H), 2.35-2.20 (m, 2H, 5-H), 2.24 (dt, *J* = 7.5, 7.5 Hz, 2H, 16-H), 2.17 (dt, *J* = 7.5, 7.5 Hz, 2H, 8-H), 2.07 (dt, *J* = 7.5, 7.5 Hz, 2H, 12-H), 2.01 (t, *J* = 7.5 Hz, 2H, 9-H), 1.99 (t, *J* = 7.5 Hz, 2H, 13-H), 1.70-1.60 (m, 2H, 4-H), 1.59 (s, 6H, 10-Me, 14-Me), 1.20 (d, *J* = 7.5 Hz, 3H, 2-Me); ^{13}C NMR ($CDCl_3$): δ 179.4 (1), 142.5 (5'), 138.8 (2'), 137.3 (6), 135.7 (14), 134.4 (10), 129.4 (7), 125.0 (3'), 124.8 (11), 123.8 (15), 111.1 (4'), 71.6 (3), 60.2 (6-CH_2), 44.4 (2), 39.7 (13), 39.6 (9), 32.4 (4), 31.6 (5), 28.4 (16), 26.6 (12), 26.2 (8), 25.0 (17), 16.0 (10-Me, 14-Me), 10.8 (2-Me); HR MS: calcd for $C_{25}H_{38}O_5Na$ (M + Na^+) 441.2611, found 441.2597.

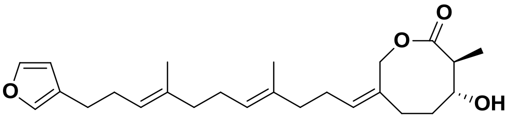

(2*S*,3*R*,6*Z*)-6-((4*E*,8*E*)-11-(furan-3-yl)-4,8-dimethylundeca-4,8-dien-1-ylidene)-3-hydroxy-2-methylheptan-7-olide (1'): To a solution of MNBA (11 mg, 32 μmol) and DMAP (9 mg, 0.16 mmol) in dichloromethane (10.0 mL) at room temperature was slowly added a solution of the seco-acid **26** (10 mg, 25 μmol) in dichloromethane (2.5 mL) with a mechanically driven syringe over a 12 h period. After cooling to 0 °C, saturated aqueous sodium hydrogencarbonate was added. The mixture was extracted with dichloromethane, and the organic layer was washed with brine and water, dried over sodium sulfate. After evaporation of the solvent, the crude product was purified by preparative TLC on silica gel (hexane/ethyl acetate = 2/1) to afford the compound **1'** (6 mg, 62%). $[\alpha]_D^{29}$ +5.64 (*c* 0.78, dichloromethane); IR (neat): 3440, 2931, 1735 cm^{-1}; ^{1}H NMR ($CDCl_3$): δ 7.34 (s, 1H, 5''-H), 7.21 (s, 1H, 2''-H), 6.28 (s, 1H, 4''-H), 5.30 (t, *J* = 7.5 Hz, 1H, 1'-H), 5.17 (t, *J* = 7.5 Hz, 1H, 9'-H), 5.11 (t, *J* = 7.5 Hz, 1H, 5'-H), 4.98 (d, *J* = 12.0 Hz, 1H, 7-H),4.70 (d, *J* = 12.0 Hz, 1H, 7-H), 3.61 (dddd, *J* = 2.5, 7.0, 7.5, 7.5 Hz, 1H, 3-H), 2.56 (dq, *J* = 7.5, 7.5 Hz, 1H, 2-H), 2.45 (t, *J* = 7.5 Hz, 2H, 11'-H), 2.36-2.28 (m, 1H, 5-H), 2.25 (dt, *J* = 7.5, 7.5 Hz, 2H, 10'-H), 2.12 (dt, *J* = 7.5, 7.5 Hz, 2H, 2'-H), 2.08 (dt, *J* = 7.5, 7.5 Hz, 2H, 6'-H), 2.01 (t, *J* = 7.5 Hz, 2H, 3'-H), 1.99 (t, *J* = 7.5 Hz, 2H, 7'-H), 1.96 (d, *J* = 7.0 Hz, 1H, OH), 1.92 (ddd, *J* = 2.5, 7.5, 15.0 Hz, 1H, 5-H), 1.86 (ddd, *J* = 2.5, 2.5, 8.5 Hz, 1H, 4-H), 1.86 (ddd, *J* = 2.5, 7.5, 7.5 Hz, 1H, 4-H), 1.59 (s, 6H, 4'-Me, 8'-Me), 1.26 (d, *J* = 7.5 Hz, 3H, 2-Me); ^{13}C NMR ($CDCl_3$): δ 176.4 (1), 142.5 (5''), 138.8 (2''), 136.0 (6), 135.7 (8'), 134.1 (4'), 129.9 (1'), 125.0 (3''), 124.9 (5'), 123.8 (9'), 111.1 (4''), 77.8 (3), 65.3 (7), 46.9 (2), 39.6 (7'), 39.3 (3'), 36.1 (4), 30.9 (5), 28.4 (10'), 26.6 (6'), 26.2 (2'), 25.0 (11'), 16.0 (4'-Me, 8'-Me), 13.9 (2-Me); HR MS: calcd for $C_{25}H_{36}O_4Na$ (M + Na^+) 423.2506, found 423.2492.

3. Preparation of alcohol 13

Alcohol **13** was prepared from (*E*,*E*)-farnesol according to the literature[2] with modification as shown below.

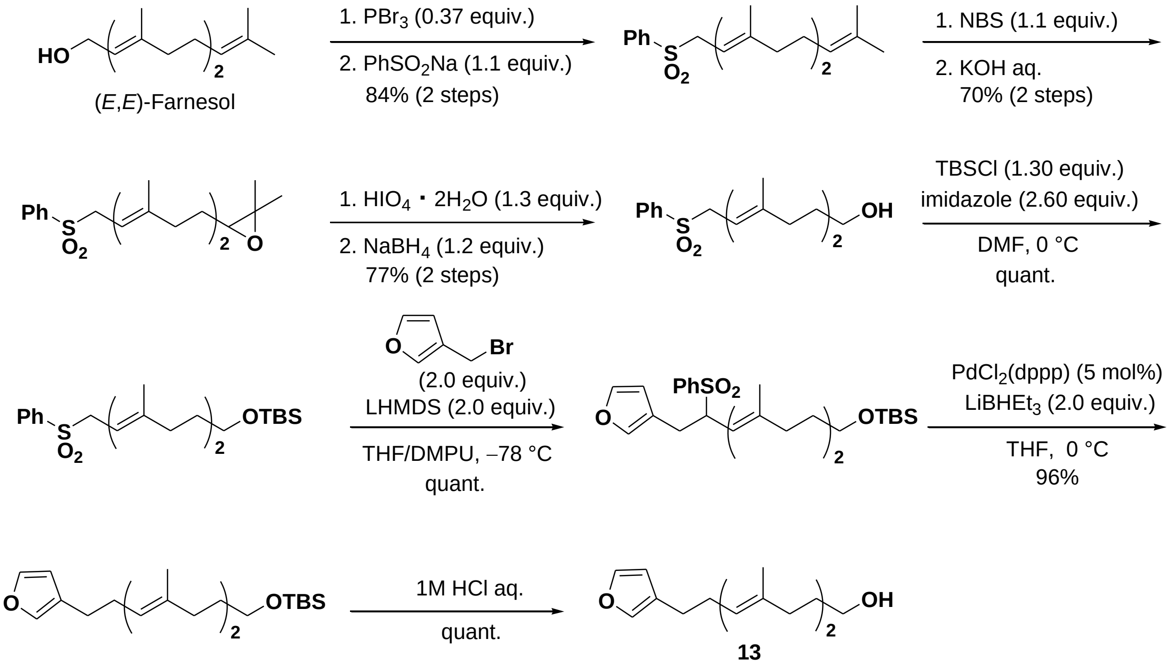

(Experimental procedures and analytical data)

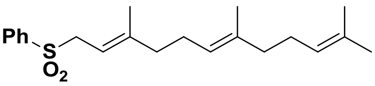

((2*E*,6*E*)-3,7,11-Trimethyldodeca-2,6,10-trien-1-yl)benzenesulfonate: To a solution of (*E*,*E*)-farnesol (19.9 g, 89 mmol) in hexane (85.0 mL) at 0 °C was added phosphorus tribromide (8.9 g,

32.8 mmol) in hexane (4.40 mL). After the reaction mixture was stirred for 40 min at 0 °C, saturated aqueous sodium hydrogencarbonate was added. The organic layer was separated and the aqueous layer was extracted with diethyl ether. The combined organic layer was washed with water and brine, and dried over Na_2SO_4. After evaporation of the solvent, the crude product was dissolved in DMF (89.4 mL) and sodium benzenesulfinate (16.1 g, 98 mmol) was added to the solution. After a stirring for 2 h, water was added and the mixture was extracted with diethyl ether, and the organic layer was washed with brine and water, dried over sodium sulfate. After evaporation of the solvent, the crude product was purified by column chromatography on silica gel (hexane/ethyl acetate = 10/1) to afford the desired sulfone (26.0 g, 84%). IR (neat): 2915, 1088 cm^{-1};^{1}H NMR ($CDCl_3$): δ 7.87 (d, *J* = 7.5 Hz, 2H, Ph), 7.63 (t, *J* = 7.5 Hz, 1H, Ph), 7.53 (dd, *J* = 7.5, 7.5 Hz, 2H, Ph), 5.20 (t, *J* = 7.5 Hz, 1H, 2-H), 5.08 (t, *J* = 7.5 Hz, 1H, 10-H), 5.05 (t, *J* = 6.0 Hz, 1H, 6-H), 3.81 (d, *J* = 7.5 Hz, 2H, 1-H), 2.09-1.95 (m, 4H, 4-H, 5-H), 2.06 (dt, *J* = 7.5, 7.5 Hz, 2H, 9-H), 1.98 (t, *J* = 7.5 Hz, 2H, 8-H), 1.68 (s, 3H, 12-Me), 1.60 (s, 3H, 11-Me), 1.58 (s, 3H, 7-Me), 1.32 (s, 3H, 4-Me); ^{13}C NMR ($CDCl_3$): δ 146.3 (Ph), 138.6 (3), 135.6 (7), 133.4 (Ph), 131.2 (11), 128.8 (Ph), 128.6 (Ph), 124.2 (10), 123.2 (8), 110.2 (2), 56.0 (1), 39.6 (4, 8), 26.6 (7-Me), 26.1 (5), 25.6 (12-Me), 17.6 (11-Me), 16.1 (3-Me), 15.9 (7-Me); HR MS: calcd for $C_{21}H_{30}O_2SNa$ (M + Na^+) 369.1859, found 369.1853.

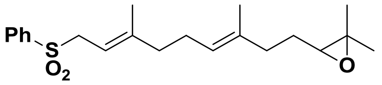

((2*E*,6*E*)-10,11-Epoxy-3,7,11-trimethyldodeca-2,6-dien-1-yl)benzenesulfonate: To a solution of ((2*E*,6*E*)-3,7,11-trimethyldodeca-2,6,10-trien-1-yl)benzenesulfonate (7.7 g, 22.2 mmol) in ^{t}BuOH / H_2O (= 7/2 v/v solution; 74 mL) at 0 °C was added *N*-bromosuccinimide (4.3 g, 24.4 mmol). After the reaction mixture was stirred for 2 h at 0 °C, the organic layer was separated and the aqueous layer was extracted with chloroform. The combined organic layer was washed with brine, and dried over Na_2SO_4. After evaporation of the solvent, the crude product was obtained, which was instantly used without further purification.

To a solution of the crude product obtained in methanol (45 mL) at 0 °C was added a 6 M aqueous solution of KOH (3.7 mL, 22.2 mmol). The mixture was stirred for 1.5 h at room temperature, and then quenched with water. The mixture was extracted with dichloromethane and dried over Na_2SO_4. After evaporation of the solvent, the crude product was purified by column chromatography on silica gel (hexane/ethyl acetate = 10/1) to afford the desired epoxide (5.6 g, 70%). IR (neat): 2938, 1234, 1088 cm^{-1}; ^{1}H NMR ($CDCl_3$): δ 7.87 (d, *J* = 7.5 Hz, 2H, Ph), 7.64 (t, *J* = 7.5 Hz, 1H, Ph), 7.53 (dd, *J* = 7.5, 7.5 Hz, 2H, Ph), 5.19 (t, *J* = 7.5 Hz, 1H, 2-H), 5.11 (t, *J* = 7.5 Hz, 1H, 5-H), 3.80 (d, *J* = 7.5 Hz, 2H, 1-H), 2.69 (t, *J* = 6.0 Hz, 1H, 10-H), 2.16 (dt, *J* = 7.5, 15.0 Hz, 1H, 8-H), 2.08 (dt, *J* = 7.5, 15.0 Hz, 1H, 8-H), 2.05-1.97 (m, 4H, 1-H, 5-H), 1.62 (dt, *J* = 6.0, 7.5 Hz, 2H, 9-H), 1.60 (s, 3H, 7-Me), 1.32 (s, 3H, 3-Me), 1.30 (s, 3H, 11-Me or 12-Me), 1.26 (s, 3H, 11-Me or 12- Me); ^{13}C NMR ($CDCl_3$): δ 146.1 (Ph), 138.6 (3), 134.6 (7), 133.4 (Ph), 128.8 (Ph), 128.3 (Ph), 123.8 (8), 110.2 (2), 63.9 (10), 58.1 (11), 55.9 (1), 39.4 (4), 36.1 (8), 27.3 (9), 26.0 (5), 24.7 (11-Me or 12-Me), 18.6 (11-Me or 12-Me), 16.0

(3-Me), 15.8 (7-Me); HR MS: calcd for $C_{21}H_{30}O_3SNa$ (M + Na^+) 385.1808, found 385.1809.

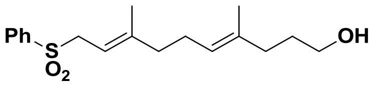

((2*E*,6*E*)-10-Hydroxy-3,7-dimethyldeca-2,6-dien-1-yl)benzenesulfonate: To a solution of ((2*E*,6*E*)-10,11-epoxy-3,7,11-trimethyldodeca-2,6-dien-1-yl)benzenesulfonate (7.6 g, 20.9 mmol) in diethyl ether (42 mL) at 0 °C was added periodic acid dihydrate (6.2 g, 27.1 mmol) in THF (21 mL). After a stirring for 2 h, diethyl ether was added. The organic layer was separated and washed with saturated aqueous sodium hydrogencarbonate and water, and dried over anhydrous Na_2SO_4. After evaporation of the solvent, the crude product was obtained, which was used in the next step without further purification.

To a solution of the crude product obtained in ethanol (42 mL) at 0 °C was added $NaBH_4$ (1.2 g, 31.3 mmol). After a stirring for 1.5 h, the reaction mixture was quenched by addition of saturated aqueous NH_4Cl and extracted with diethyl ether. The organic layer was separated and dried over Na_2SO_4. After evaporation of the solvent, the crude product was purified by column chromatography on silica gel (hexane/ethyl acetate = 5/1) to afford the title compound (5.2 g, 77%). IR (neat): 3541, 2931, 1080 cm^{-1}; ^1H NMR ($CDCl_3$): δ 7.88 (d, *J* = 7.5 Hz, 2H, Ph), 7.64 (t, *J* = 7.5 Hz, 1H, Ph), 7.54 (dd, *J* = 7.5, 7.5 Hz, 2H, Ph), 5.20 (t, *J* = 7.0 Hz, 1H, 2-H), 5.11 (t, *J* = 7.5 Hz, 1H, 6-H), 3.80 (d, *J* = 7.0 Hz, 2H, 1-H), 3.62 (t, *J* = 6.0 Hz, 2H, 10-H), 2.06 (t, *J* = 7.5 Hz, 2H, 8-H), 2.04 (dt, *J* = 6.0, 7.5 Hz, 2H, 5-H), 2.03 (m, 2H, 4-H), 1.67 (tt, *J* = 6.0, 7.5 Hz, 2H, 9-H), 1.60 (s, 3H, 7-Me), 1.33 (s, 3H, 3-Me); ^{13}C NMR ($CDCl_3$): δ 146.1 (Ph), 138.5 (3), 135.2 (7), 133.4 (Ph), 128.8 (Ph), 128.3 (Ph), 123.4 (6), 110.1 (2), 62.2 (10), 55.9 (1), 39.4 (4), 35.6 (8), 30.5 (9), 25.8 (5), 15.9 (3-Me), 15.7 (7-Me); HR MS: calcd for $C_{18}H_{26}O_3SNa$ (M + Na^+) 345.1495, found 345.1508.

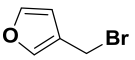

3-(Bromomethyl)furan: In a similar mannar as described before,[3] to a solution of furan-3-ylmethanol (0.43 mL, 5.0 mmol) in 5 mL of THF at 0 °C was added 0.25 mL of phosphorous tribromide (PBr_3) (7.46 M in hexane). After a stirring for 1 h at 0 °C, the reaction mixture was purified by flash column chromatography on silica gel (hexane/ethyl acetate = 10/1) to afford the desired bromide (0.75 g, 93%), which was instantly used in the next reaction.

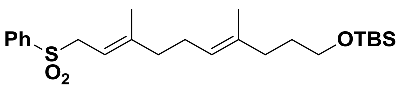

((2*E*,6*E*)-10-(*tert*-Butyldimethylsiloxy)-3,7-dimethyldeca-2,6-dien-1-yl)benzenesulfonate: To a solution of ((2*E*,6*E*)-10-hydroxy-3,7-dimethyldeca-2,6-dien-1-yl)benzenesulfonate (10.2 g, 31.6 mmol) and imidazole (5.60 g, 82.2 mmol) in DMF (63.2 mL) was added *tert*-butyldimethylsilylchloride (TBSCl) (6.19 g, 41.1 mmol) at 0 °C. After a stirring for 1.5 h, the

reaction mixture was quenched by addition of saturated aqueous NH_4Cl and extracted with ethyl acetate. The organic layer was separated and dried over Na_2SO_4. After evaporation of the solvent, the crude product was purified by column chromatography on silica gel (hexane/ethyl acetate = 10/1) to afford the title compound (13.8 g, quant.). IR (neat): 2931, 1095 cm^{-1}; ^{1}H NMR ($CDCl_3$): δ 7.87 (d, *J* = 7.5 Hz, 2H, Ph), 7.64 (t, *J* = 7.5 Hz, 1H, Ph), 7.54 (dd, *J* = 7.5, 7.5 Hz, 2H, Ph), 5.19 (t, *J* = 8.5 Hz, 1H, 2-H), 5.06 (m, 1H, 6-H), 3.81 (d, *J* = 8.5 Hz, 2H, 1-H), 3.58 (t, *J* = 6.0 Hz, 2H, 10-H), 2.05-1.97 (m, 6H, 4-H, 5-H, 8-H), 1.60 (tt, *J* = 6.0, 7.5 Hz, 2H, 9-H),1.58 (s, 3H, 7-Me), 1.32 (s, 3H, 3-Me), 0.90 (s, 9H, TBS), 0.04 (s, 6H, TBS); ^{13}C NMR ($CDCl_3$): δ 146.3 (Ph), 138.7 (3), 135.4 (7), 133.5 (Ph), 128.9 (Ph), 128.5 (Ph), 123.3 (6), 110.3 (2), 62.8 (10), 56.1 (1), 39.6 (4), 35.7 (8), 31.1 (9), 26.1 (5), 25.9 (TBS), 18.3 (TBS), 16.1 (3-Me), 15.9 (7-Me), -5.3 (TBS); HR MS: calcd for $C_{24}H_{40}O_3SSiNa$ (M + Na^+) 459.2360, found 459.2363.

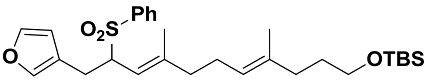

((2*E*,6*E*)-10-(*tert*-Butyldimethylsiloxy)-1-(furan-3-yl)methyl-3,7-dimethyldeca-2,6-dien-1-yl) benzenesulfonate: To a solution of ((2*E*,6*E*)-10-(*tert*-butyldimethylsiloxy)-3,7-dimethyldeca-2,6-dien-1-yl)benzenesulfonate (0.71 g, 1.63 mmol) in THF (9.0 mL) was added *N*,*N*'-dimethylpropyleneurea (DMPU) (0.74 mL, 6.20 mmol), a solution of 3-(bromomethyl)furan (0.52 g, 3.25 mmol) in THF (2.0 mL), and lithium bis(trimethylsilyl)amide (LHMDS) (3.3 mL) (1.0 M in THF) at -78 °C. After a stirring for 1.0 h, the reaction mixture was quenched by addition of saturated aqueous NH_4Cl and extracted with ethyl acetate. The organic layer was separated and dried over Na_2SO_4. After evaporation of the solvent, the crude product was purified by thin layer chromatography on silica (hexane/ethyl acetate = 5/1) to afford the title compound (0.90 g, quant.). IR (neat): 2931, 1095 cm^{-1}; ^{1}H NMR ($CDCl_3$): δ 7.86 (d, *J* = 7.5 Hz, 2H, Ph), 7.63 (t, *J* = 7.5 Hz, 1H, Ph), 7.52 (dd, *J* = 7.5, 7.5 Hz, 2H, Ph), 7.29 (s, 1H, 1-H), 7.18 (s, 1H, 2'-H), 6.20 (s, 1H, 4'-H), 5.05-4.95 (m, 2H, 2-H, 6-H), 3.90 (ddd, *J* = 3.5, 11.0, 11.0 Hz, 1H, 1-H), 3.58 (t, *J* = 6.5 Hz, 2H, 10-H), 3.36 (dd, *J* = 3.5, 15.0 Hz, 1H, 1-CH_2), 2.75 (dd, *J* = 11.0, 15.0 Hz, 1H, 1-CH_2), 1.98 (t, *J* = 7.0 Hz, 2H, 8-H), 1.95-1.89 (m, 4H, 4-H, 5-H), 1.58 (tt, *J* = 6.5, 7.0 Hz, 2H, 9-H), 1.57 (s, 3H, 7-Me), 1.07 (s, 3H, 3-Me), 0.89 (s, 9H, TBS), 0.04 (s, 6H, TBS); ^{13}C NMR ($CDCl_3$): δ 146.0 (Ph), 142.8 (5'), 140.0 (3-Me), 137.8 (3), 135.5 (7), 133.5 (Ph), 129.2 (Ph), 128.7 (Ph), 123.3 (6), 120.2 (8), 117.0 (4'), 110.9 (2), 65.1 (1), 62.9 (10), 39.6 (8), 35.8 (8), 31.2 (9), 26.1 (5), 25.9 (TBS), 23.5 (1-CH_2), 18.3 (TBS), 16.3 (3-Me), 15.9 (7-Me), -5.3 (TBS); HR MS: calcd for $C_{29}H_{44}O_4SSiNa$ (M + Na^+) 539.2622, found 539.2644.

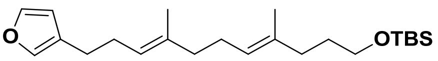

(4*E*,8*E*)-3-(1-(*tert*-Butyldimethylsiloxy)-4,8-dimethylundeca-4,8-dien-11-yl)furan: To a solution of ((2*E*,6*E*)-10-(*tert*-butyldimethylsiloxy)-1-(furan-3-yl)methyl-3,7-dimethyldeca-2,6-dien-1-yl) benzenesulfonate (3.2 mg, 6.13 mmol) and $PdCl_2$(dppp) (181 mg, 0.31 mmol) in THF (61 mL) was added $LiBHEt_3$ (12.3 mL) (1.0 M in THF) at 0 °C. After a stirring for 1.0 h, the reaction mixture was quenched by 3 M aqueous solution of sodium hydroxide and aqueous solution of potassium cyanide. After an additonal stirring for 0.5 h, phosphate buffer solution and the mixture was extracted with ethyl acetate and dried over Na_2SO_4. After evaporation of the solvent, the crude product was purified by thin layer chromatography on silica (hexane/ethyl acetate = 5/1) to afford the title compound (2.2 g, 96%). IR (neat): 2931 cm^{-1}; ^{1}H NMR ($CDCl_3$): δ 7.34 (s, 1H, 5'-H), 7.21 (s, 1H, 2'-H), 6.28 (s, 1H, 4'-H), 5.17 (t, *J* = 7.5 Hz, 1H, 3-H), 5.11 (t, *J* = 7.5 Hz, 1H, 7-H), 3.58 (t, *J* = 6.5 Hz, 2H, 11-H), 2.45 (t, *J* = 7.5 Hz, 2H, 1-H), 2.24 (dt, *J* = 7.5, 7.5 Hz, 2H, 2-H), 2.08 (dt, *J* = 7.5, 7.5 Hz, 2H, 6-H), 2.03-1.96 (m, 4H, 5-H, 9-H), 1.61 (tt, *J* = 6.5, 7.0 Hz, 2H, 10-H), 1.60 (s, 6H, 4-Me, 8-Me), 0.90 (s, 9H, TBS), 0.05 (s, 6H, TBS); ^{13}C NMR ($CDCl_3$): δ 142.5 (5'), 138.8 (2'), 135.7 (4), 134.7 (8), 125.0 (3'), 124.2 (7), 123.7 (3), 111.1 (4'), 62.9 (11), 39.7 (5), 35.8 (9), 31.2 (10), 28.4 (2), 26.6 (6), 26.0 (TBS), 25.0 (1), 18.3 (TBS), 16.0 (4-Me, 8-Me), -5.3 (TBS); HR MS: calcd for $C_{23}H_{40}O_2SiNa$ (M + Na^+) 399.2690, found 399.2698.

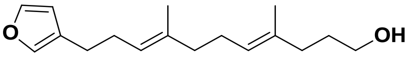

(4*E*,8*E*)-11-(Furan-3-yl)-4,8-dimethyl-4,8-undecadien-1-ol (13): To a solution of (4*E*,8*E*)-3-(1-(*tert*-butyldimethylsiloxy)-4,8-dimethylundeca-4,8-dien-4-yl)furan in methanol (6.6 mL) was added a 1 M aqueous solution of HCl (0.66 mL). After a stirring for 0.5 h, the reaction mixture was quenched by phosphate buffer solution and the mixture was extracted with ethyl acetate and dried over Na_2SO_4. After evaporation of the solvent, the crude product was purified by preparative TLC on silica gel (hexane/ethyl acetate = 2/1) to afford the title compound (192 mg, quant.). IR (neat): 3410, 2931 cm^{-1}; ^{1}H NMR ($CDCl_3$): δ 7.34 (s, 1H, 5'-H), 7.21 (s, 1H, 2'-H), 6.28 (s, 1H, 4'-H), 5.16 (t, *J* = 7.5 Hz, 1H, 9-H), 5.14 (t, *J* = 7.5 Hz, 1H, 5-H), 3.63 (dt, *J* = 6.0, 6.5 Hz, 2H, 1-H), 2.45 (t, *J* = 7.5 Hz, 2H, 12-H), 2.24 (dt, *J* = 7.5, 7.5 Hz, 2H, 10-H), 2.09 (dt, *J* = 7.5, 7.5 Hz, 2H, 6-H), 2.06 (t, *J* = 7.0 Hz, 2H, 3-H), 2.00 (t, *J* = 7.5 Hz, 2H, 7-H), 1.67 (tt, *J* = 6.5, 7.0 Hz, 2H, 2-H), 1.61 (s, 3H, 8-Me), 1.59 (s, 3H, 4-Me), 1.28 (t, *J* = 6.0 Hz, 1H, OH); ^{13}C NMR ($CDCl_3$): δ 142.5 (5'), 138.8 (2'), 135.6 (8), 134.7 (4), 125.0 (3'), 124.7 (5), 123.9 (9), 111.1 (4'), 62.8 (1), 39.6 (7), 36.0 (3), 30.7 (2), 28.4 (10), 26.4 (6), 25.0 (11), 16.0 (8-Me), 15.9 (4-Me); HR MS: calcd for $C_{17}H_{26}O_2Na$ (M + Na^+) 285.1825, found 285.1814.

4. Supplementary Tables and Figures

Supplementary Table 1. ^{1}H and ^{13}C NMR data for the natural and synthetic **1** (chemical shifts in ppm; $CDCl_3$)

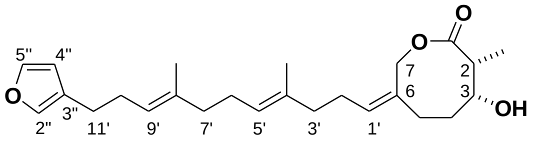

2,3-*cis*-Astakolactin (**1**)

position	natural δH	natural δC	synthetic (**1**) δH	synthetic (**1**) δC	position	natural δH	natural δC	synthetic (**1**) δH	synthetic (**1**) δC
5"	7.32	142.5	7.33	142.5	4'-Me	1.55	15.9	1.59	16.0
4"	6.26	111.1	6.27	111.1	3'	1.95	39.6	2.01	39.4
3"		124.8		125.0	2'	2.05	25.7	2.12	26.2
2"	7.19	138.8	7.21	138.8	1'	5.21	125.6	5.33	130.5
11'	2.43	25.0	2.45	25.0	7	4.67; 3.83	67.3	5.08; 4.60	65.7
10'	2.23	32.4	2.24	28.4	6		131.9		136.2
9'	5.14	123.8	5.17	123.8	5	2.26	28.4	2.30; 2.01	29.9
8'		135.7		135.7	4	1.73; 1.48	29.1	1.90; 1.80	35.1
8'-Me	1.55	16.0	1.59	16.0	3	3.68	78.0	4.06	74.1
7'	1.99	39.6	1.99	39.6	2	2.70	43.8	2.96	42.5
6'	2.05	26.5	2.08	26.6	2-Me	1.18	12.1	1.22	11.6
5'	5.07	124.8	5.10	124.9	1		175.0		176.6
4'		134.2		134.1					

Supplementary Figure 1 (as Figure 2 in the main text). Δδ (ppm) of ^{1}H NMR chemical shifts in **1**. Δδ corresponds to the difference in chemical shift for natural and synthetic products (Δδ = δ(synthetic)–δ(natural)).

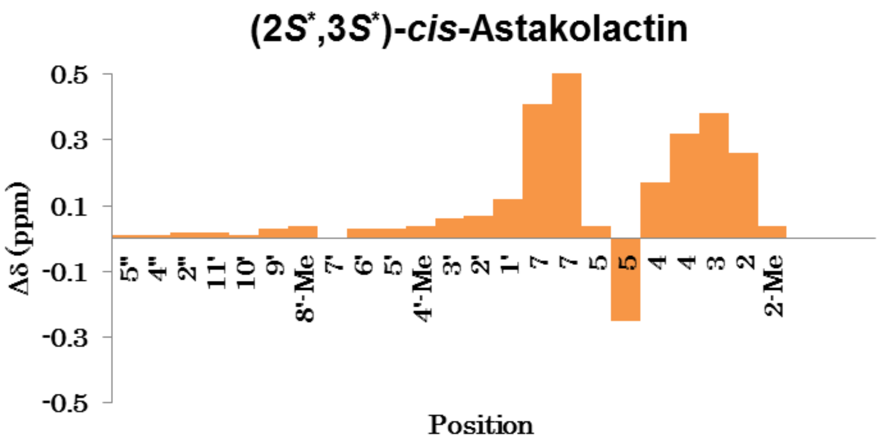

Supplementary Table 2. ^{1}H and ^{13}C NMR data for the natural and synthetic **1'** (chemical shifts in ppm; $CDCl_3$)

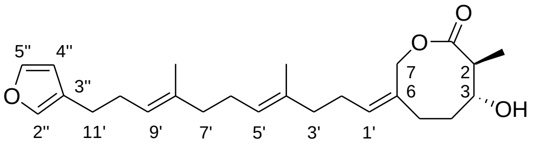

2,3-*trans*-astakolactin (**1'**)

	natural		synthetic (**1'**)			natural		synthetic (**1'**)	
position	δ_H	δ_C	δ_H	δ_C	position	δ_H	δ_C	δ_H	δ_C
5"	7.32	142.5	7.34	142.5	4'-Me	1.55	15.9	1.59	16.0
4"	6.26	111.1	6.28	111.1	3'	1.95	39.6	2.01	39.3
3"		124.8		125.0	2'	2.05	25.7	2.12	26.2
2"	7.19	138.8	7.21	138.8	1'	5.21	125.6	5.30	129.9
11'	2.43	25.0	2.45	25.0	7	4.67; 3.83	67.3	4.98; 4.70	65.3
10'	2.23	32.4	2.25	28.4	6		131.9		136.0
9'	5.14	123.8	5.17	123.8	5	2.26	28.4	2.34; 1.92	30.9
8'		135.7		135.7	4	1.73; 1.48	29.1	1.86	36.1
8'-Me	1.55	16.0	1.59	16.0	3	3.68	78.0	3.61	77.8
7'	1.99	39.6	1.99	39.6	2	2.70	43.8	2.56	46.9
6'	2.05	26.5	2.08	26.6	2-Me	1.18	12.1	1.26	13.9
5'	5.07	124.8	5.11	124.9	1		175.0		176.4
4'		134.2		134.1					

Supplementary Figure 2 (as Figure 3 in the main text). Δδ (ppm) of ^{1}H NMR chemical shifts in **1'**. Δδ corresponds to the difference in chemical shift for natural and synthetic products (Δδ = δ(synthetic)–δ(natural)).

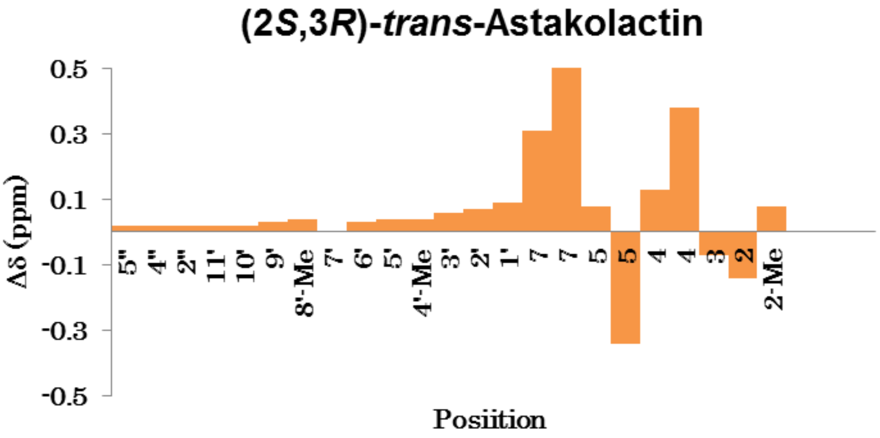

5. References

(1) (a) Shiina, I.; Kubota, M.; Oshiumi, H.; Hashizume, M. *J. Org. Chem.* **2004**, *69*, 1822–1830. (b) Shiina, I.; Umezaki, Y.; Kuroda, N.; Iizumi, T.; Nagai, S.; Katoh, T. *J. Org. Chem.* **2012**, *77*, 4885–4901. (c) Shiina, I.; Kawakita, Y. *Tetrahedron* **2004**, *60*, 4729–4733. (d) Shiina, I.; Ushiyama, H.; Yamada, Y.; Kawakita, Y.; Nakata, K. *Chem. Asian J.* **2008**, *3*, 454–461.

(2) Takabe, K.; Hashimoto, H.; Sugimoto, H.; Nomoto, M.; Yoda, H. *Tetrahedron*: *Asymmetry* **2004**, *15*, 909–912.

(3) Aggarwal, V. K.; Vasse, J.-L. *Org. Lett.* **2003**, *5*, 3987–3990.

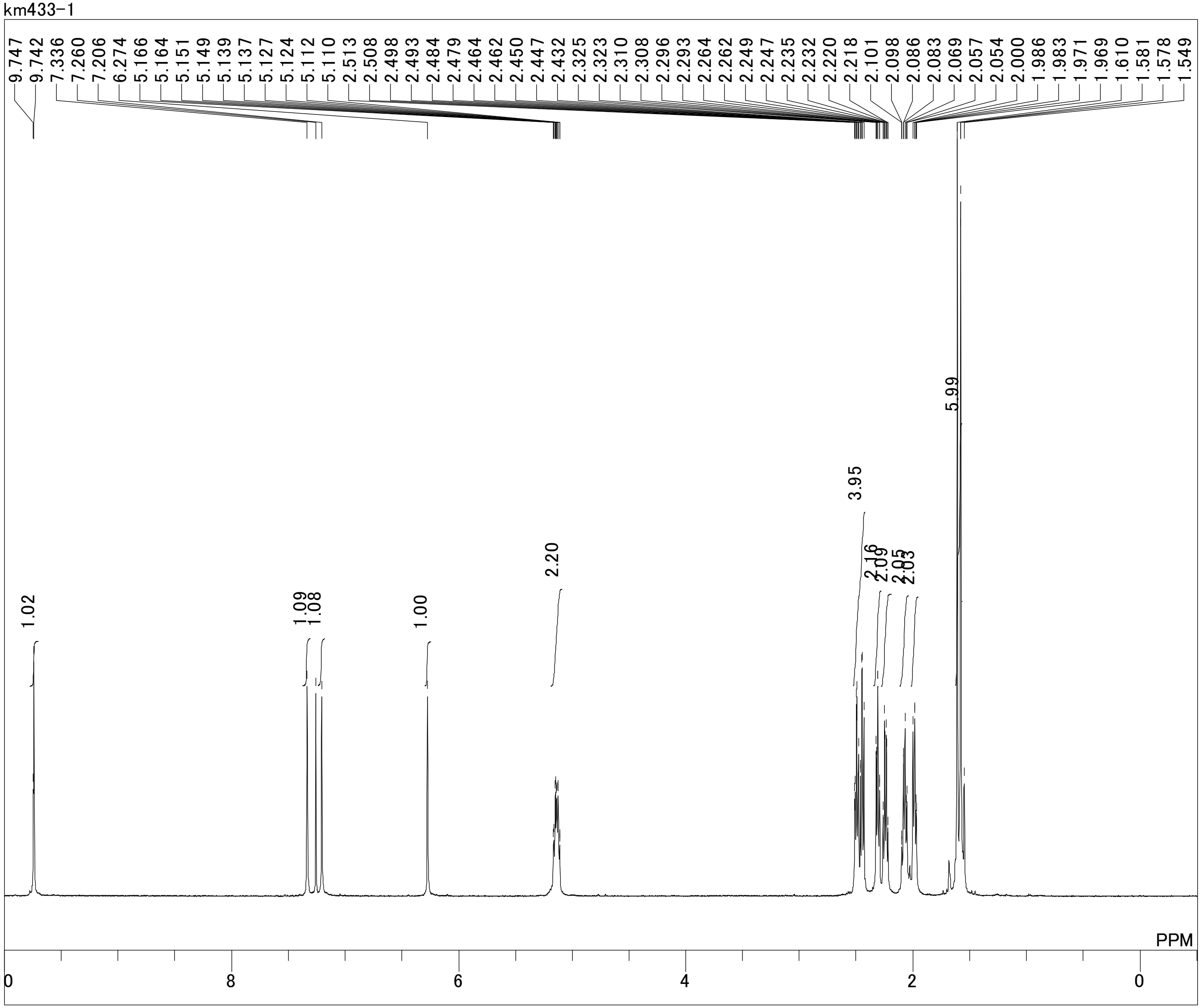

DFILE F:¥mameda¥NMR¥km433-1.als
COMNT km433-1
DATIM Mon Dec 12 15:08:41 2011
OBNUC 1H
EXMOD non
OBFRQ 500.00 MHz
OBSET 0.00 KHz
OBFIN 162160.00 Hz
POINT 8192
FREQU 10000.00 Hz
SCANS 8
ACQTM 0.8192 sec
PD 6.1808 sec
PW1 6.20 usec
IRNUC 1H
CTEMP 24.0 c
SLVNT CDCL3
EXREF 7.26 ppm
BF 0.12 Hz
RGAIN 20

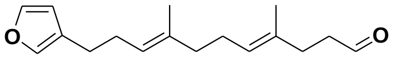

(4*E*,8*E*)-11-(Furan-3-yl)-4,8-dimethylundeca-4,8-dienal (**6**)

km433-1

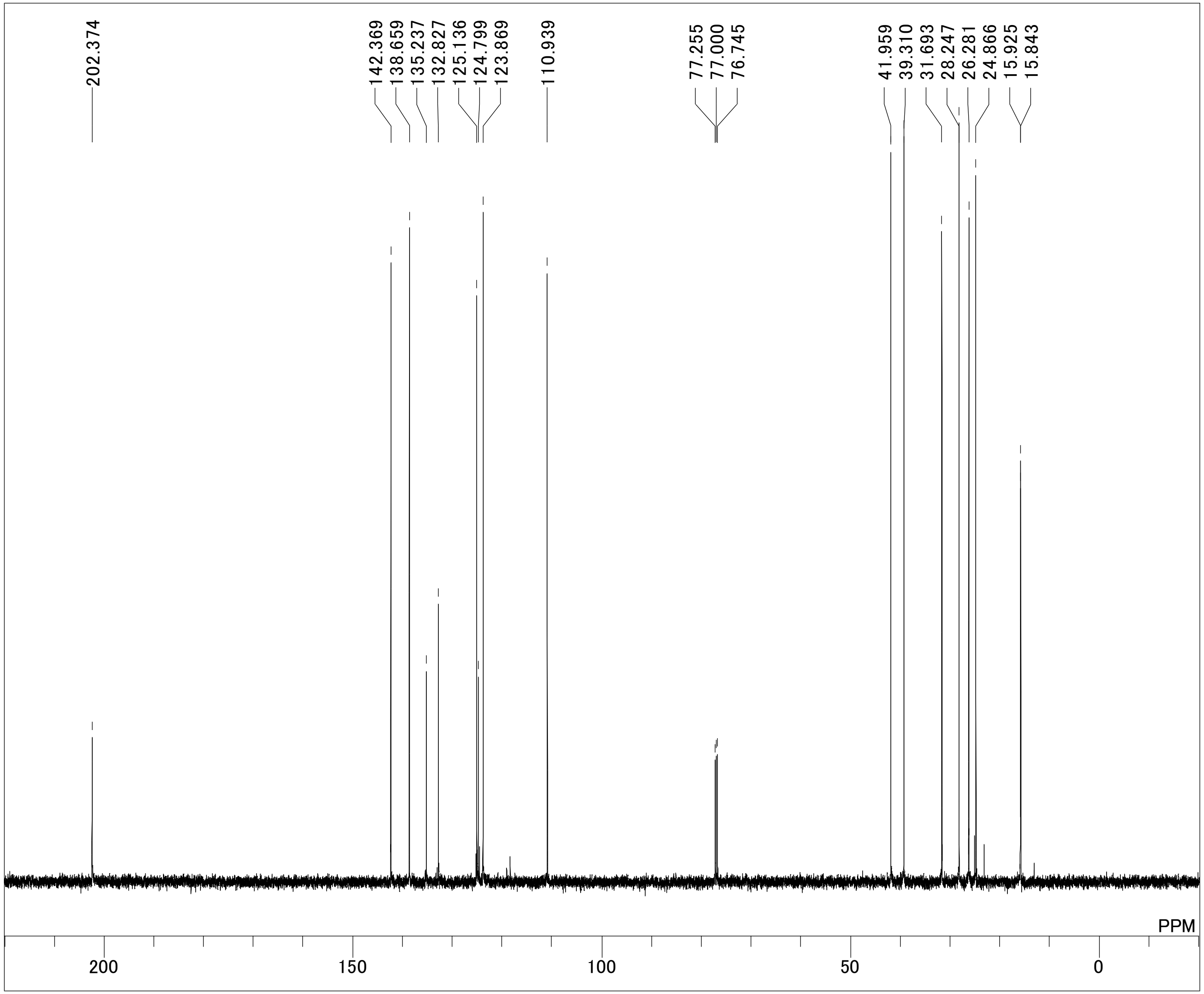

DFILE	F:¥mameda¥NMR¥km433-1(13C).als
COMNT	km433-1
DATIM	Mon Dec 12 15:41:33 2011
OBNUC	13C
EXMOD	bcm
OBFRQ	125.65 MHz
OBSET	0.00 KHz
OBFIN	127958.00 Hz
POINT	32768
FREQU	33898.30 Hz
SCANS	64
ACQTM	0.9667 sec
PD	2.0333 sec
PW1	4.90 usec
IRNUC	1H
CTEMP	25.4 c
SLVNT	CDCL3
EXREF	77.00 ppm
BF	1.20 Hz
RGAIN	30

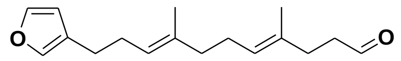

(4*E*,8*E*)-11-(Furan-3-yl)-4,8-dimethylundeca-4,8-dienal (**6**)

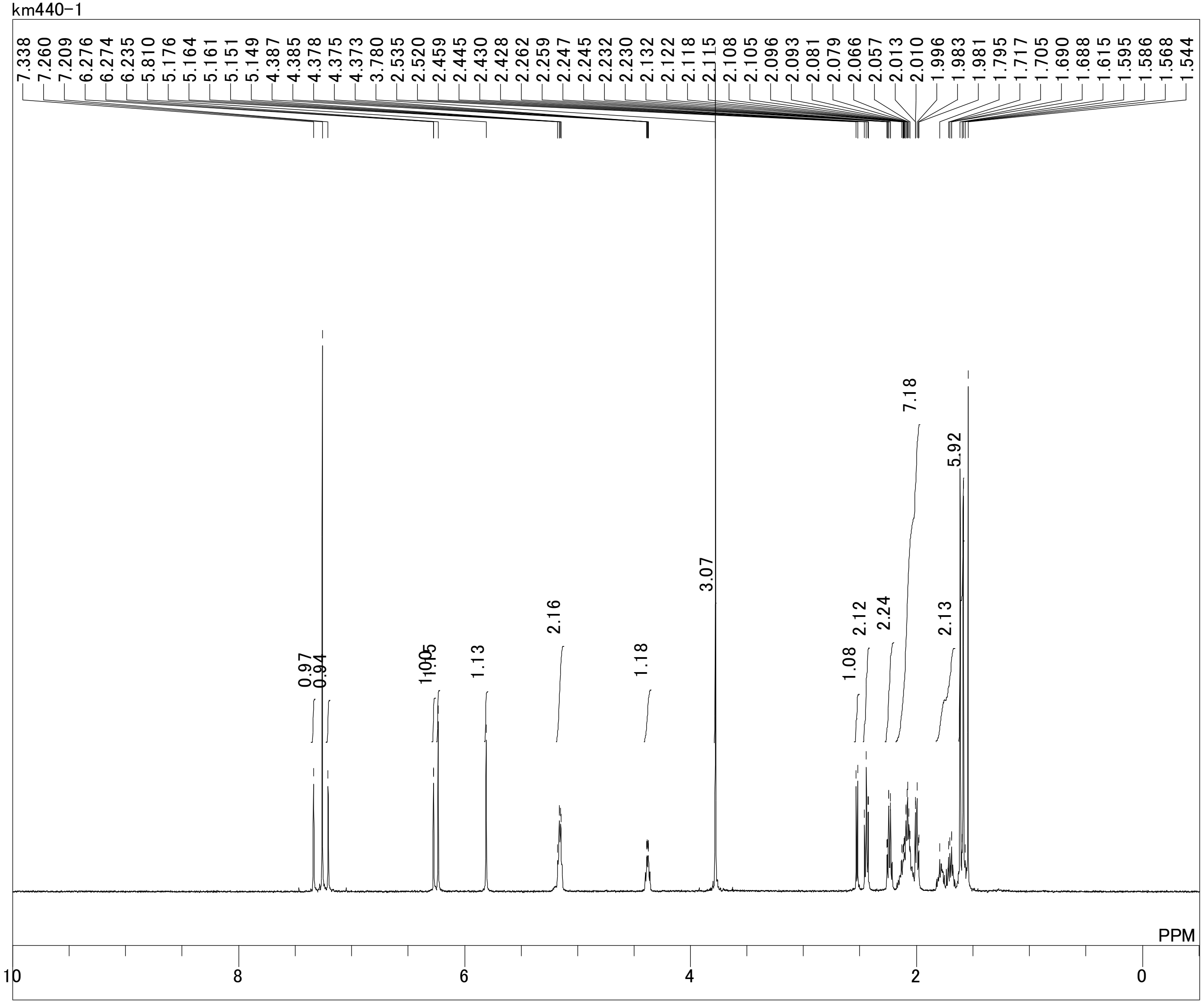

DFILE F:¥mameda¥NMR¥km440-1.als
COMNT km440-1
DATIM Mon Dec 19 19:08:05 2011
OBNUC 1H
EXMOD non
OBFRQ 500.00 MHz
OBSET 0.00 KHz
OBFIN 162160.00 Hz
POINT 8192
FREQU 10000.00 Hz
SCANS 8
ACQTM 0.8192 sec
PD 6.1808 sec
PW1 6.20 usec
IRNUC 1H
CTEMP 23.0 c
SLVNT CDCL3
EXREF 7.26 ppm
BF 0.12 Hz
RGAIN 25

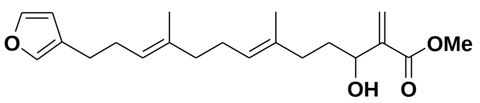

Methyl (6*E*,10*E*)-13-(furan-3-yl)-3-hydroxy-6,10-dimethyl-2-methylenetrideca-6,10-dienoate (**5**)

km440-1

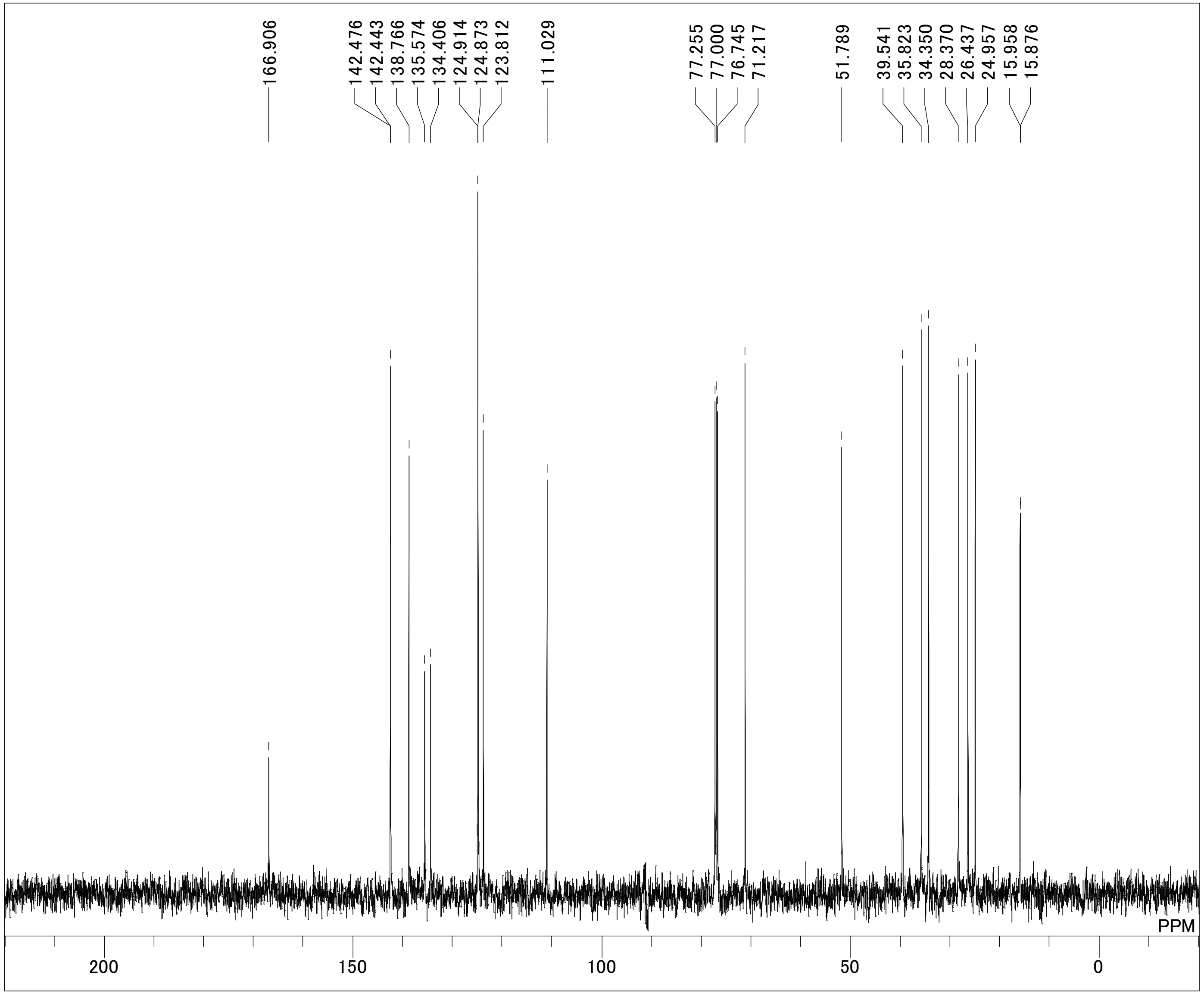

DFILE	F:¥mameda¥NMR¥km440-1(13C).als
COMNT	km440-1
DATIM	Mon Dec 19 19:33:20 2011
OBNUC	13C
EXMOD	bcm
OBFRQ	125.65 MHz
OBSET	0.00 KHz
OBFIN	127958.00 Hz
POINT	32768
FREQU	33898.30 Hz
SCANS	64
ACQTM	0.9667 sec
PD	2.0333 sec
PW1	4.90 usec
IRNUC	1H
CTEMP	25.2 c
SLVNT	CDCL3
EXREF	77.00 ppm
BF	4.20 Hz
RGAIN	30

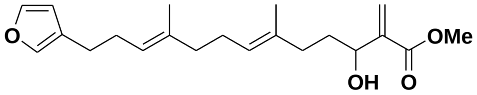

Methyl (6*E*,10*E*)-13-(furan-3-yl)-3-hydroxy-6,10-dimethyl-2-methylenetrideca-6,10-dienoate (**5**)

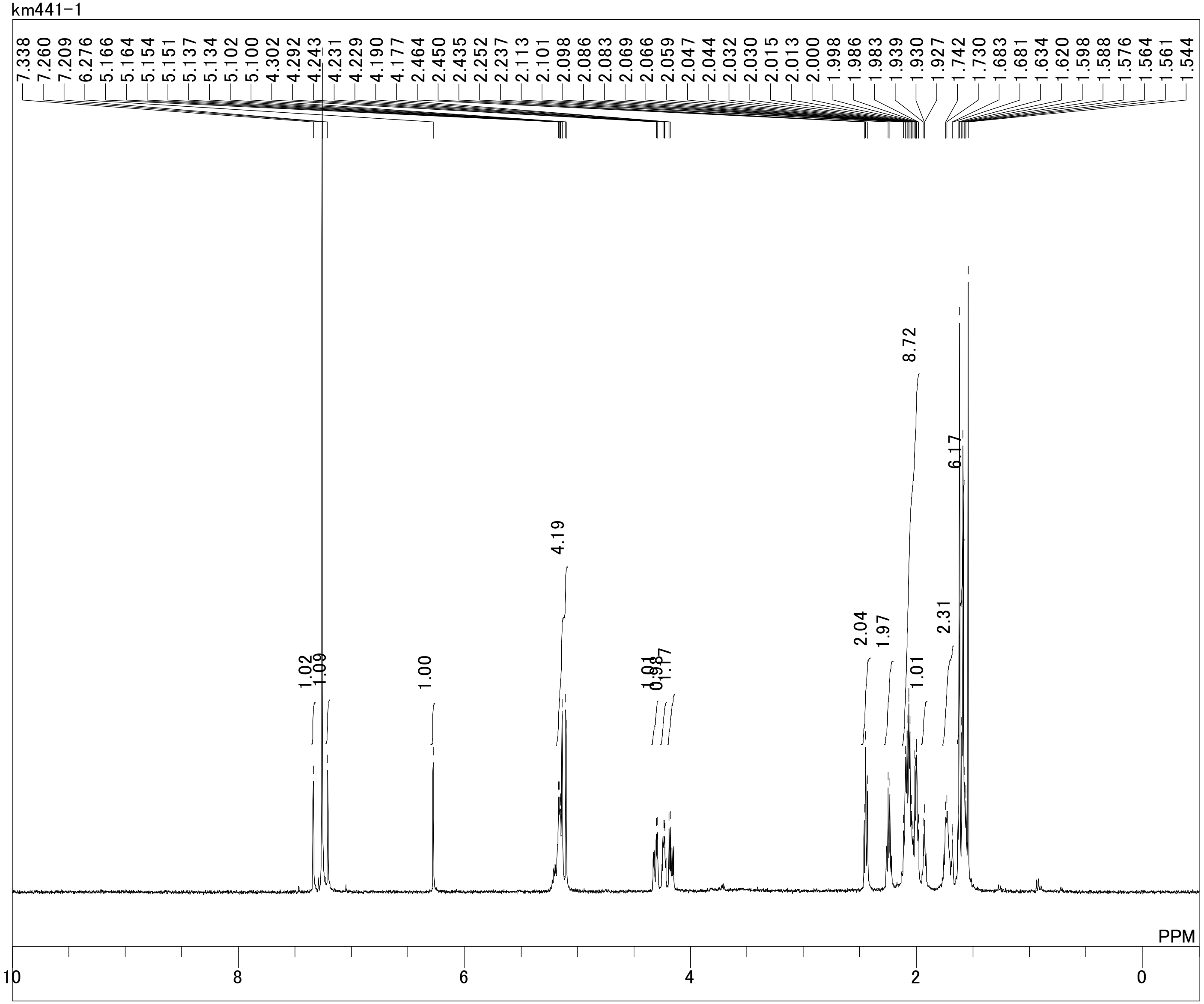

DFILE	F:¥mameda¥NMR¥km441-1.als
COMNT	km441-1
DATIM	Tue Dec 20 15:17:39 2011
OBNUC	1H
EXMOD	non
OBFRQ	500.00 MHz
OBSET	0.00 KHz
OBFIN	162160.00 Hz
POINT	8192
FREQU	10000.00 Hz
SCANS	8
ACQTM	0.8192 sec
PD	6.1808 sec
PW1	6.20 usec
IRNUC	1H
CTEMP	23.8 c
SLVNT	CDCL3
EXREF	7.26 ppm
BF	0.12 Hz
RGAIN	25

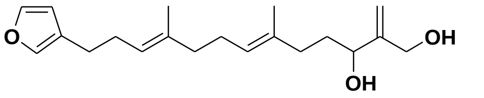

(6*E*,10*E*)-13-(Furan-3-yl)-6,10-dimethyl-2-methylenetrideca-6,10-diene-1,3-diol (**15**)

km441-1

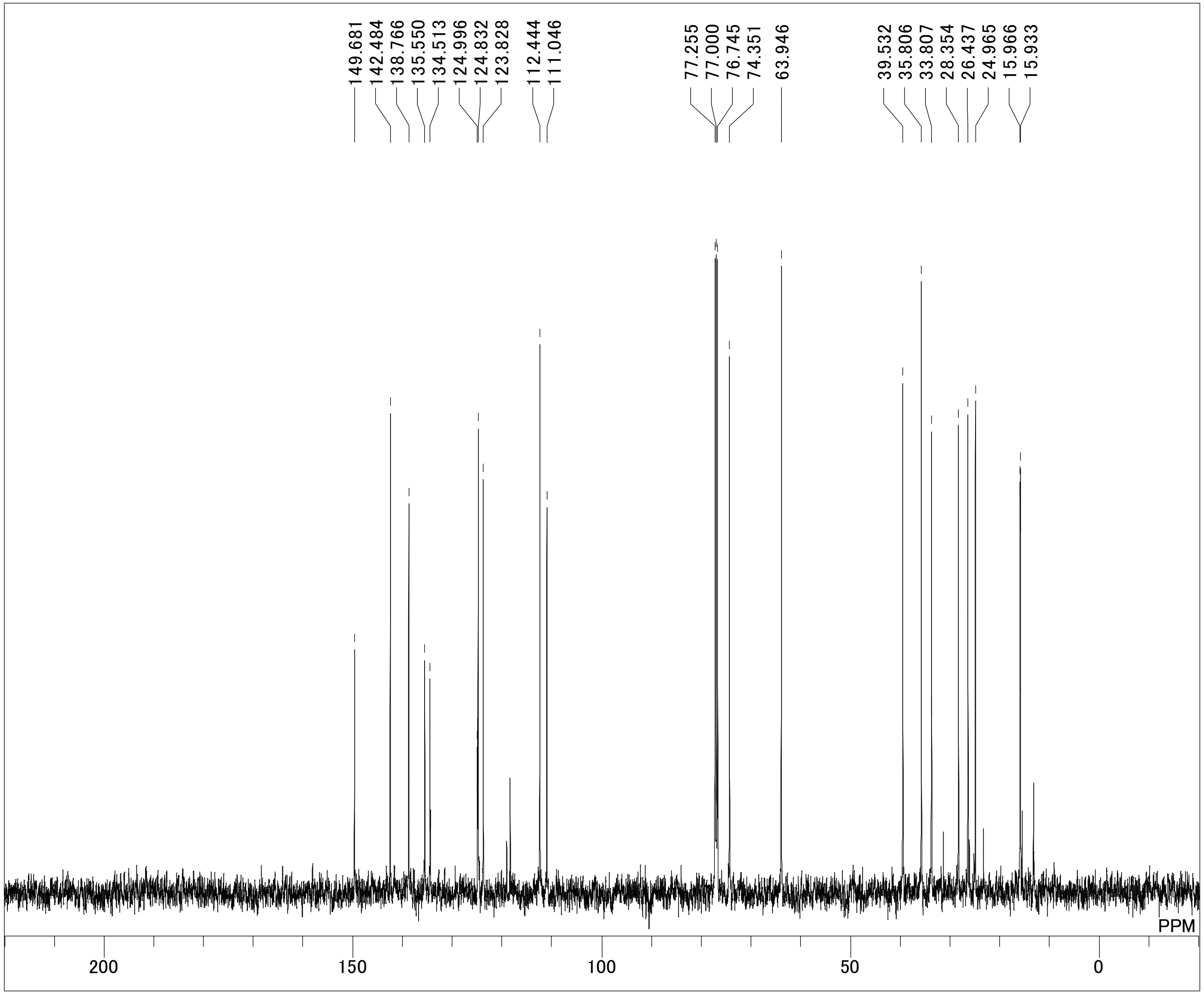

DFILE	F:¥mameda¥NMR¥km441-1(13C).als
COMNT	km441-1
DATIM	Tue Dec 20 15:46:12 2011
OBNUC	13C
EXMOD	bcm
OBFRQ	125.65 MHz
OBSET	0.00 KHz
OBFIN	127958.00 Hz
POINT	32768
FREQU	33898.30 Hz
SCANS	128
ACQTM	0.9667 sec
PD	2.0333 sec
PW1	4.90 usec
IRNUC	1H
CTEMP	25.2 c
SLVNT	CDCL3
EXREF	77.00 ppm
BF	4.20 Hz
RGAIN	30

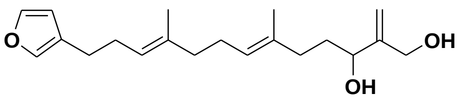

(6*E*,10*E*)-13-(Furan-3-yl)-6,10-dimethyl-2-methylenetrideca-6,10-diene-1,3-diol (**15**)

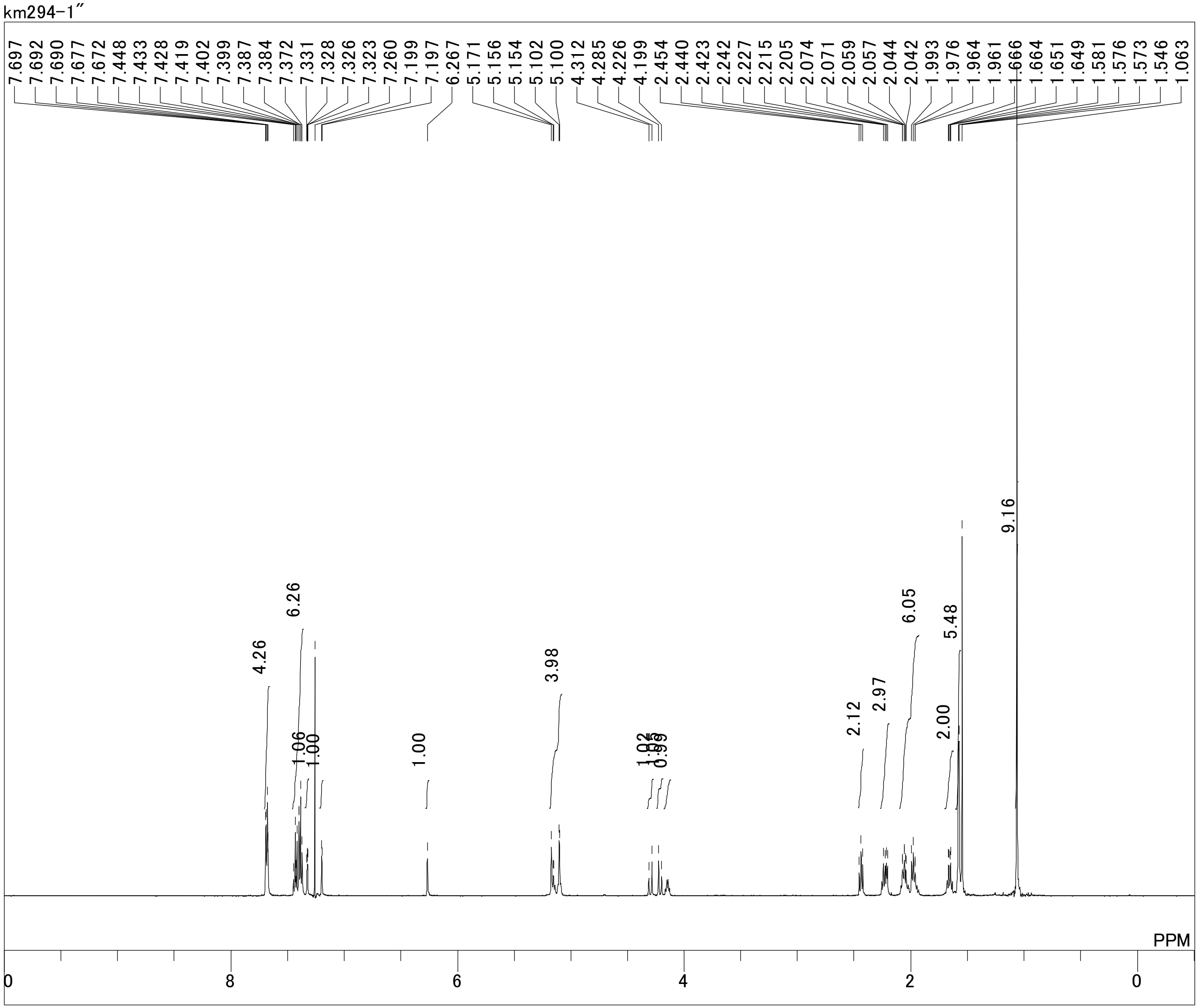

DFILE F:¥mameda¥NMR¥km294-1″.als
COMNT km294-1″
DATIM Mon Jul 25 10:20:51 2011
OBNUC 1H
EXMOD non
OBFRQ 500.00 MHz
OBSET 0.00 KHz
OBFIN 162160.00 Hz
POINT 8192
FREQU 10000.00 Hz
SCANS 8
ACQTM 0.8192 sec
PD 6.1808 sec
PW1 6.20 usec
IRNUC 1H
CTEMP 25.7 c
SLVNT CDCL3
EXREF 7.26 ppm
BF 0.12 Hz
RGAIN 22

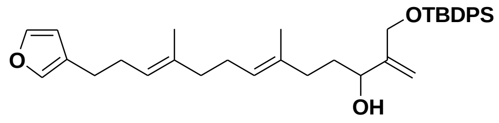

(6*E*,10*E*)-2-(*tert*-Butyldiphenylsiloxy)methyl-13-(furan-3-yl)-6,10-dimethyl-trideca-1,6,10-trien-3-ol (**16**)

km294-1"(13C)

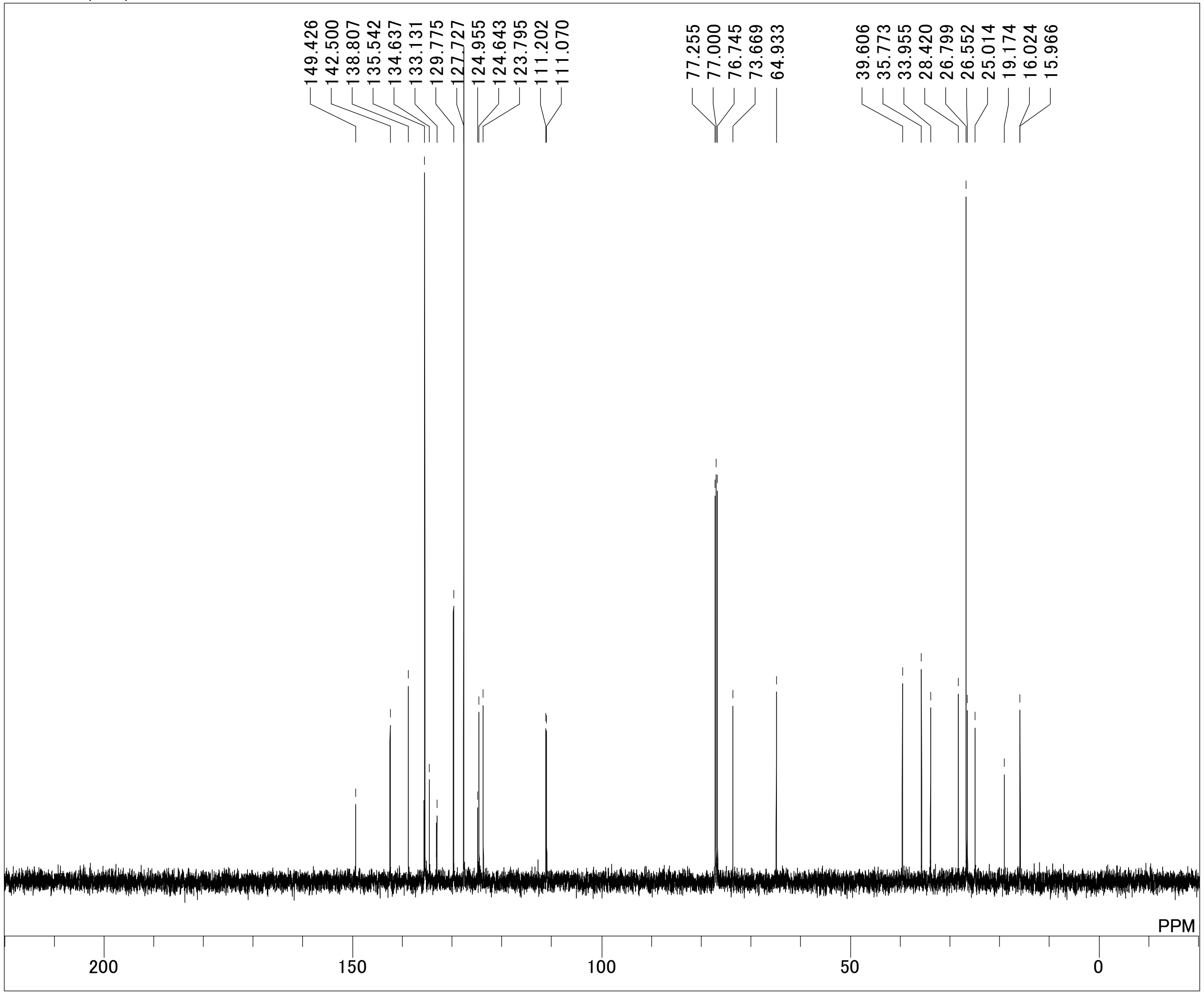

DFILE	F:¥mameda¥NMR¥km294-1"(13C).als
COMNT	km294-1"(13C)
DATIM	Mon Jul 25 10:41:06 2011
OBNUC	13C
EXMOD	bcm
OBFRQ	125.65 MHz
OBSET	0.00 KHz
OBFIN	127958.00 Hz
POINT	32768
FREQU	33898.30 Hz
SCANS	128
ACQTM	0.9667 sec
PD	2.0333 sec
PW1	4.90 usec
IRNUC	1H
CTEMP	27.6 c
SLVNT	CDCL3
EXREF	77.00 ppm
BF	1.20 Hz
RGAIN	31

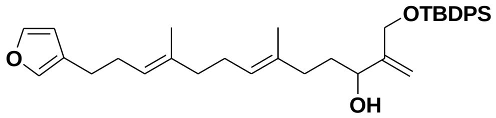

(6*E*,10*E*)-2-(*tert*-Butyldiphenylsiloxy)methyl-13-(furan-3-yl)-6,10-dimethyl-trideca-1,6,10-trien-3-ol (**16**)

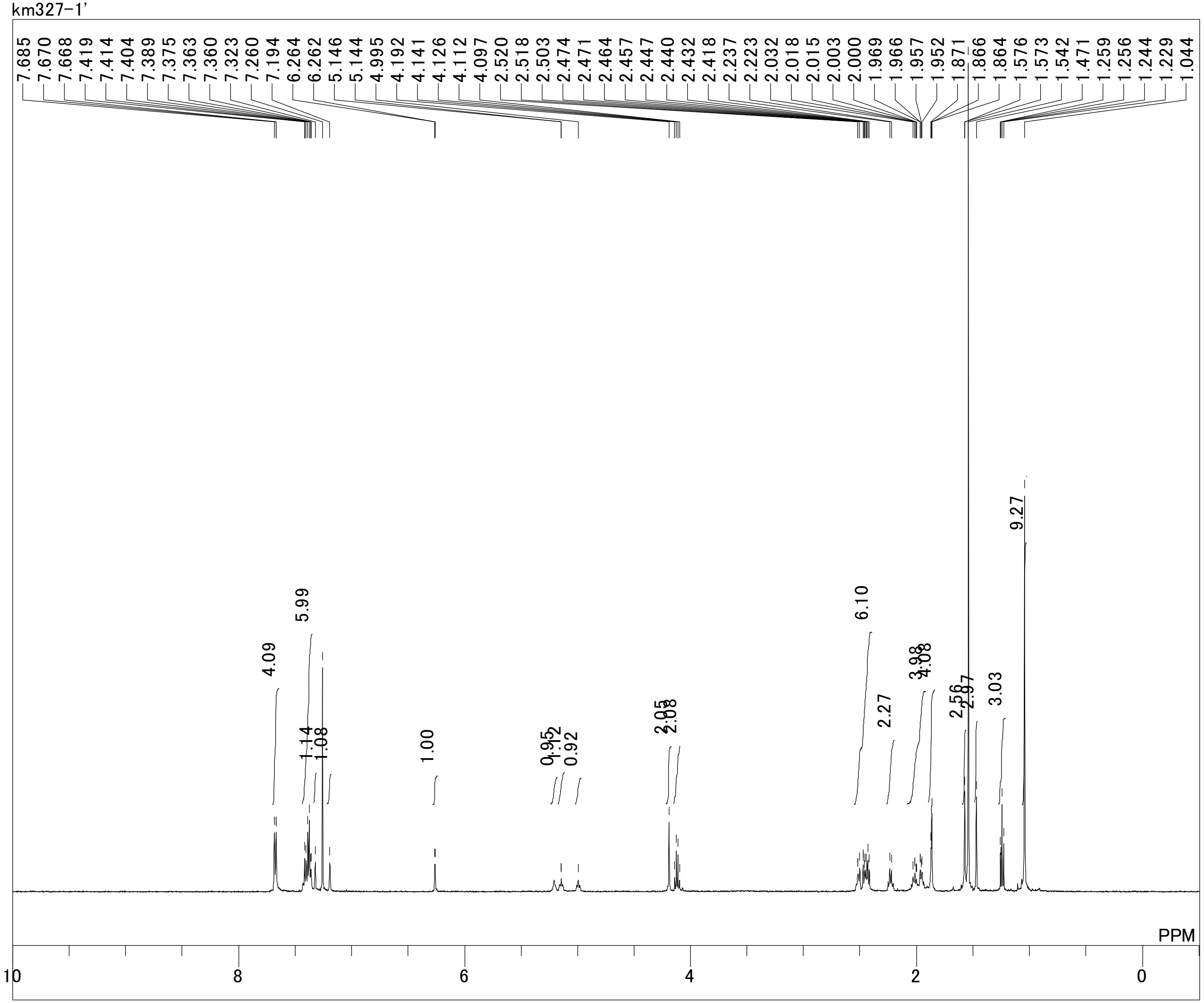

DFILE	F:¥mameda¥NMR¥km327-1'.als
COMNT	km327-1'
DATIM	Wed Jul 20 14:16:15 2011
OBNUC	1H
EXMOD	non
OBFRQ	500.00 MHz
OBSET	0.00 KHz
OBFIN	162160.00 Hz
POINT	8192
FREQU	10000.00 Hz
SCANS	8
ACQTM	0.8192 sec
PD	6.1808 sec
PW1	6.20 usec
IRNUC	1H
CTEMP	27.4 c
SLVNT	CDCL3
EXREF	7.26 ppm
BF	0.12 Hz
RGAIN	25

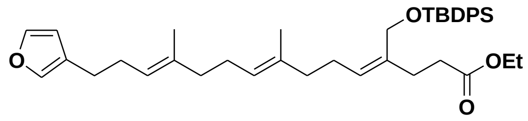

Ethyl (4*Z*,8*E*,12*E*)-4-(*tert*-butyldiphenylsiloxy)methyl-15-(furan-3-yl)-8,12-dimethylpentadeca-4,8,12-trienoate (**17**)

km327-1'(13C)

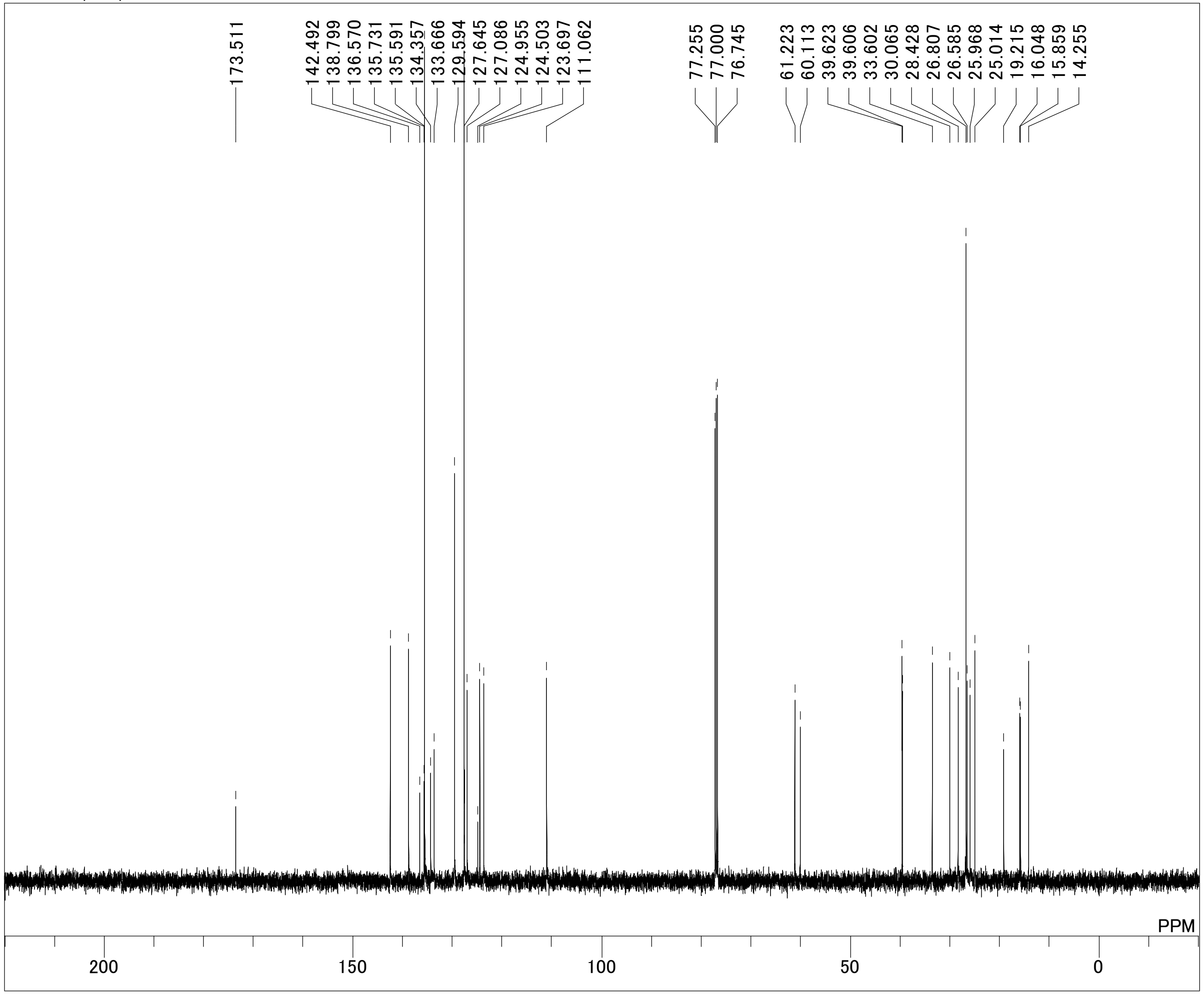

DFILE F:¥mameda¥NMR¥km327-1'(13C).als
COMNT km327-1'(13C)
DATIM Wed Jul 20 14:32:54 2011
OBNUC 13C
EXMOD bcm
OBFRQ 125.65 MHz
OBSET 0.00 KHz
OBFIN 127958.00 Hz
POINT 32768
FREQU 33898.30 Hz
SCANS 248
ACQTM 0.9667 sec
PD 2.0333 sec
PW1 4.90 usec
IRNUC 1H
CTEMP 29.4 c
SLVNT CDCL3
EXREF 77.00 ppm
BF 1.20 Hz
RGAIN 31

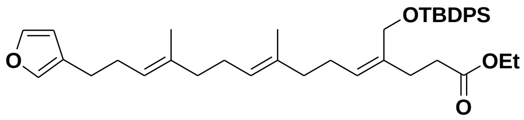

Ethyl (4*Z*,8*E*,12*E*)-4-(*tert*-butyldiphenylsiloxy)methyl-15-(furan-3-yl)-8,12-dimethylpentadeca-4,8,12-trienoate (**17**)

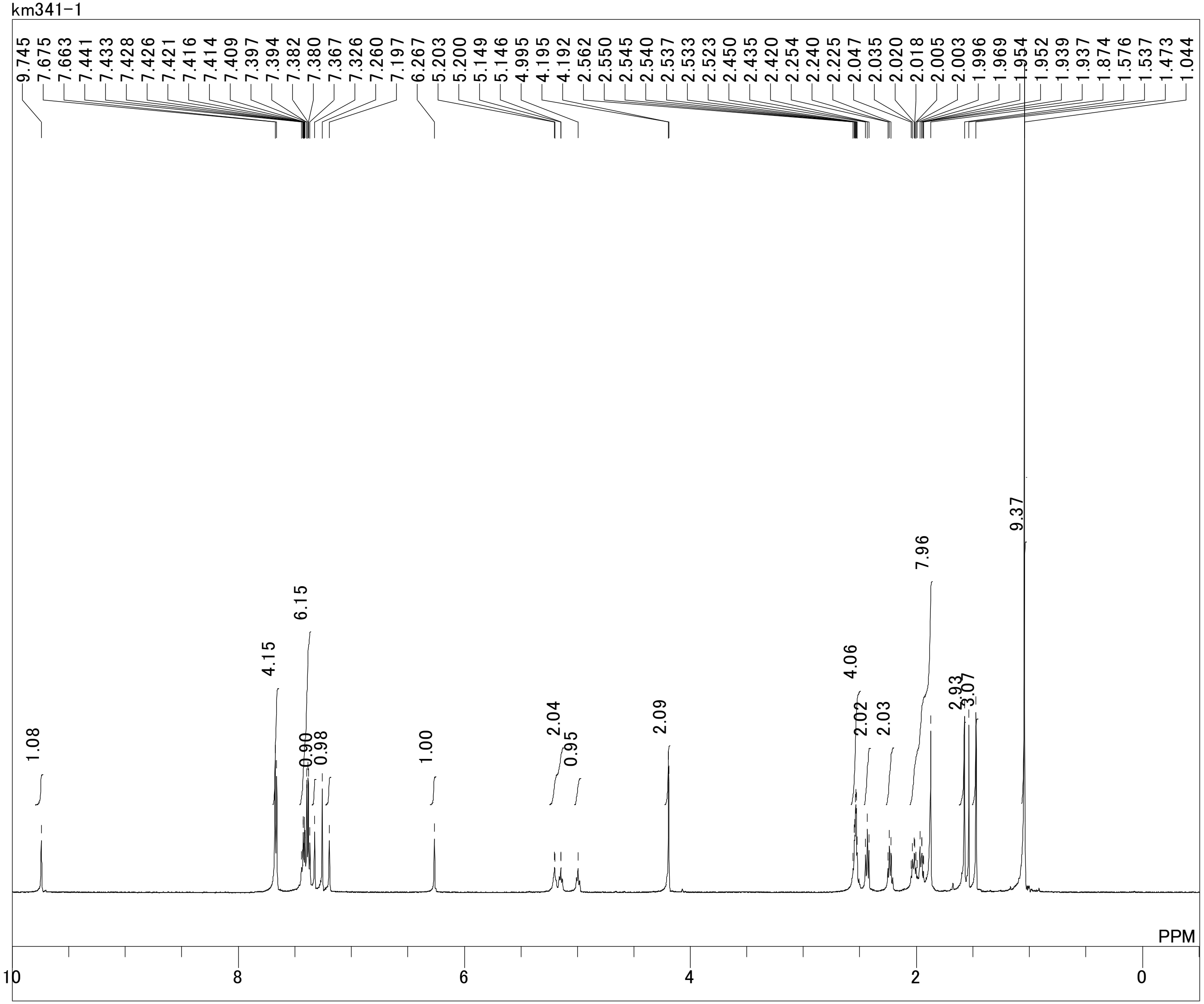

DFILE F:¥mameda¥NMR¥km341-1.als
COMNT km341-1
DATIM Thu Jul 21 17:50:45 2011
OBNUC 1H
EXMOD non
OBFRQ 500.00 MHz
OBSET 0.00 KHz
OBFIN 162160.00 Hz
POINT 8192
FREQU 10000.00 Hz
SCANS 8
ACQTM 0.8192 sec
PD 6.1808 sec
PW1 6.20 usec
IRNUC 1H
CTEMP 25.8 c
SLVNT CDCL3
EXREF 7.26 ppm
BF 0.12 Hz
RGAIN 22

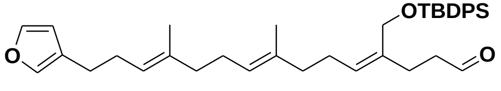

(4*Z*,8*E*,12*E*)-4-(*tert*-Butyldiphenylsiloxy)methyl-15-(furan-3-yl)-8,12-dimethylpentadeca-4,8,12-trienal (**18**)

km341-1(13C)

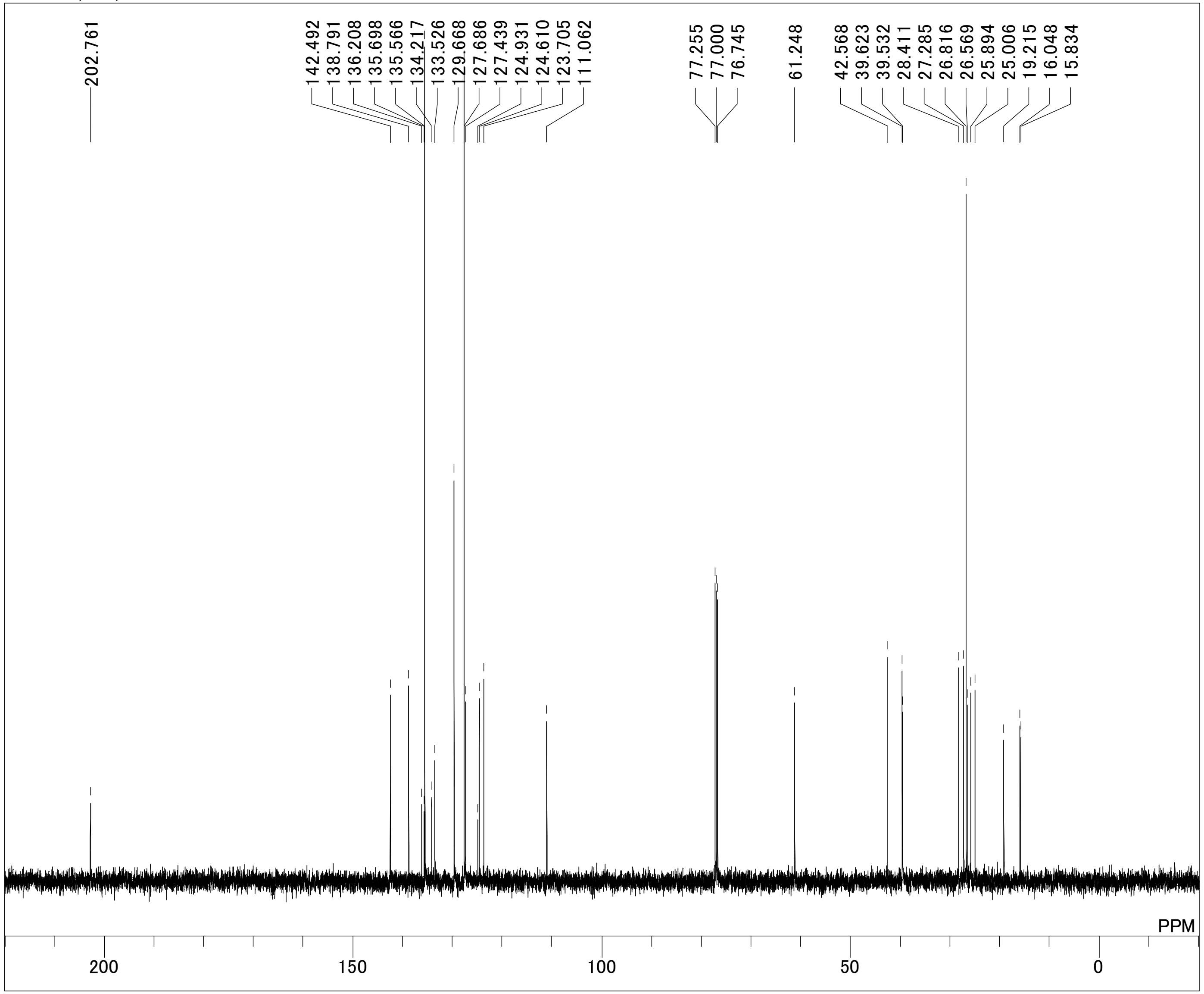

DFILE	F:¥mameda¥NMR¥km341-1(13C).als
COMNT	km341-1(13C)
DATIM	Thu Jul 21 18:00:04 2011
OBNUC	13C
EXMOD	bcm
OBFRQ	125.65 MHz
OBSET	0.00 KHz
OBFIN	127958.00 Hz
POINT	32768
FREQU	33898.30 Hz
SCANS	64
ACQTM	0.9667 sec
PD	2.0333 sec
PW1	4.90 usec
IRNUC	1H
CTEMP	27.3 c
SLVNT	CDCL3
EXREF	77.00 ppm
BF	1.20 Hz
RGAIN	30

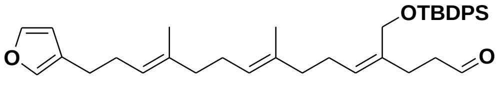

(4*Z*,8*E*,12*E*)-4-(*tert*-Butyldiphenylsiloxy)methyl-15-(furan-3-yl)-8,12-dimethylpentadeca-4,8,12-trienal (**18**)

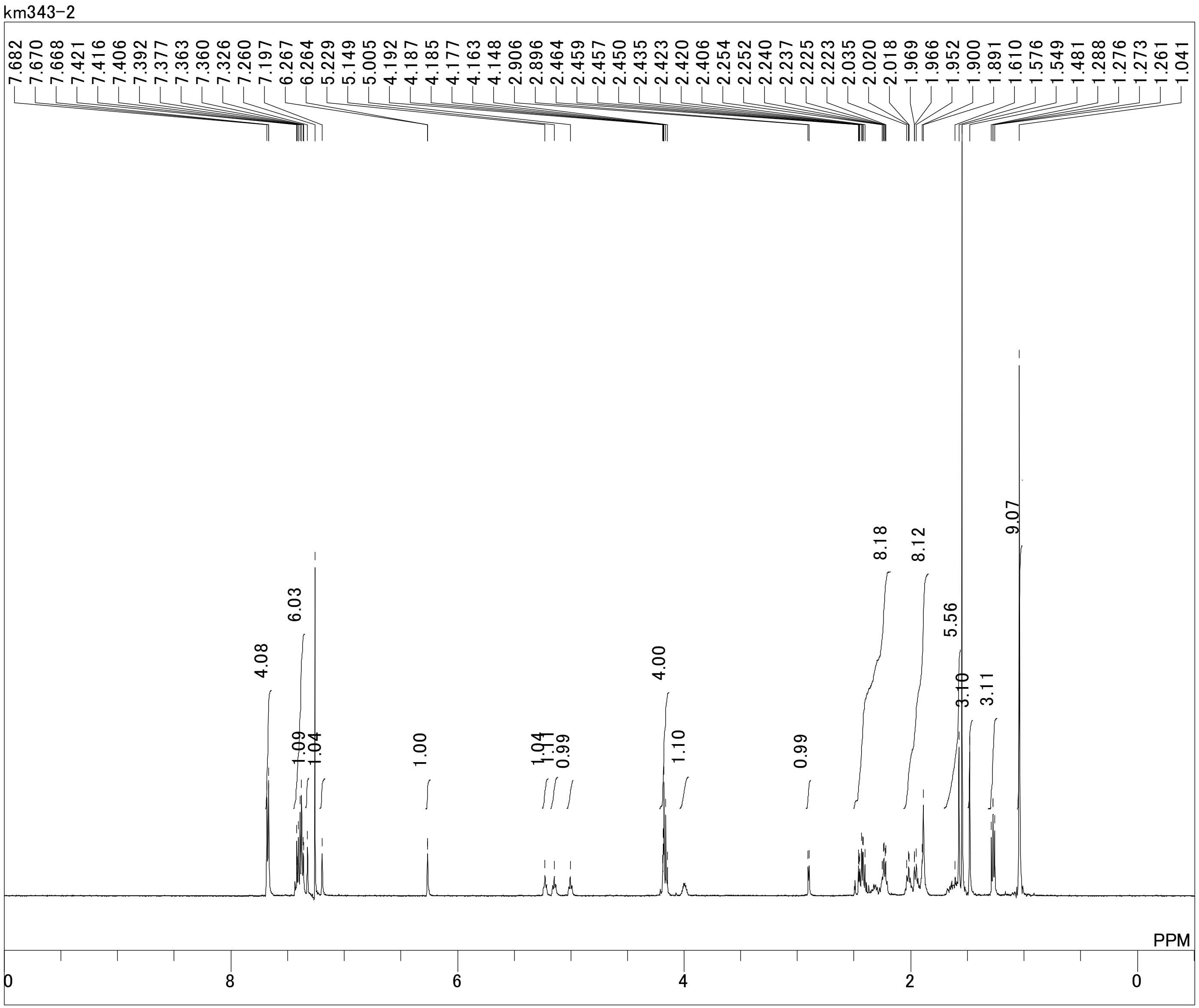

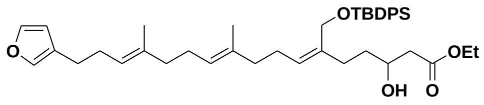

Ethyl (6*Z*,10*E*,14*E*)-6-(*tert*-butyldiphenylsiloxy)methyl-17-(furan-3-yl)-3-hydroxy-10,14-dimethylheptadeca-6,10,14-trienoate (**19**)

km343-2(13C)

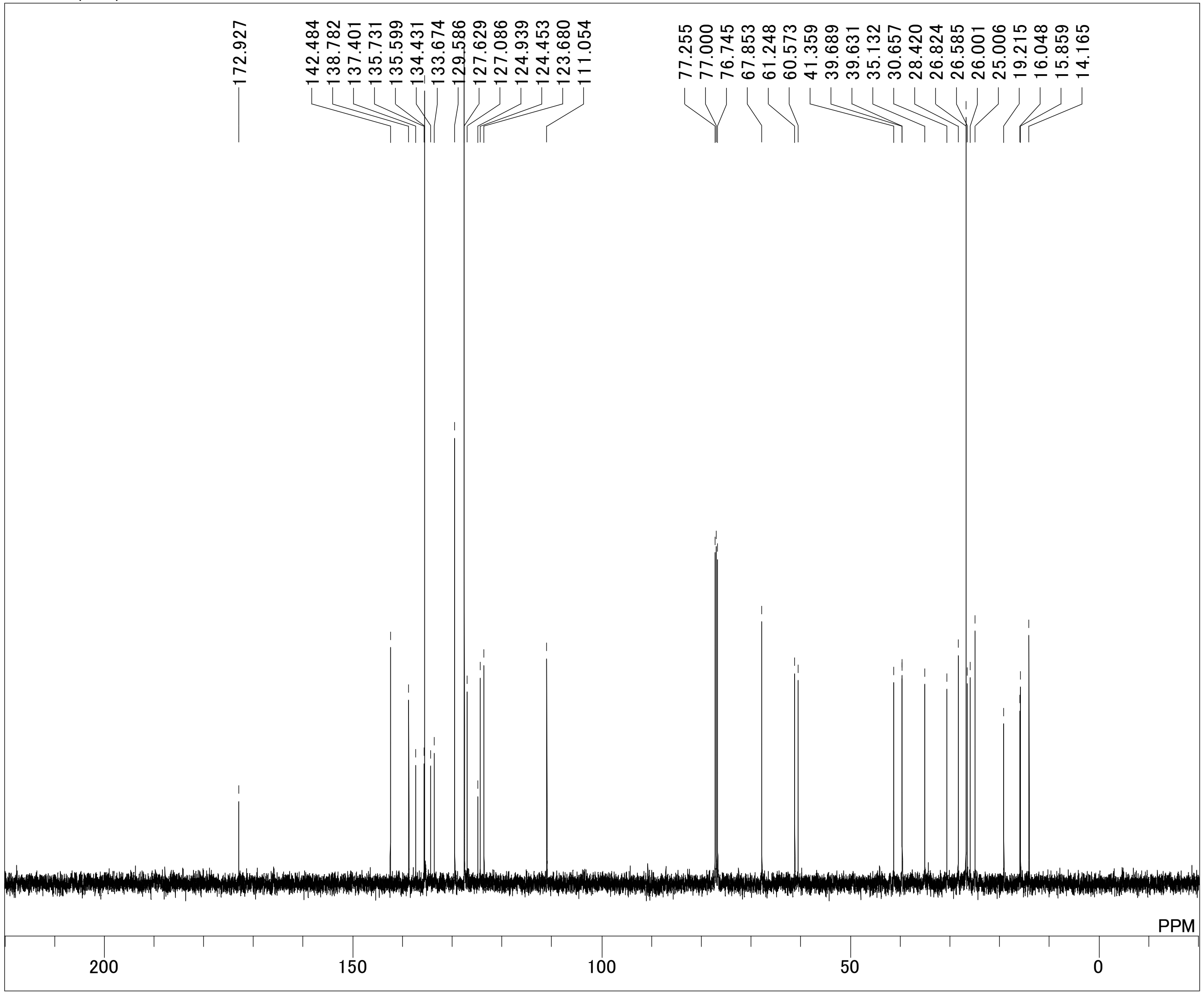

DFILE	F:¥mameda¥NMR¥km343-2(13C).als
COMNT	km343-2(13C)
DATIM	Mon Jul 25 10:30:54 2011
OBNUC	13C
EXMOD	bcm
OBFRQ	125.65 MHz
OBSET	0.00 KHz
OBFIN	127958.00 Hz
POINT	32768
FREQU	33898.30 Hz
SCANS	128
ACQTM	0.9667 sec
PD	2.0333 sec
PW1	4.90 usec
IRNUC	1H
CTEMP	27.4 c
SLVNT	CDCL3
EXREF	77.00 ppm
BF	1.20 Hz
RGAIN	30

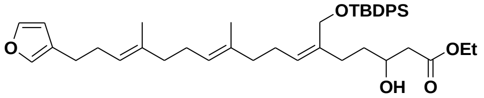

Ethyl (6*Z*,10*E*,14*E*)-6-(*tert*-butyldiphenylsiloxy)methyl-17-(furan-3-yl)-3-hydroxy-10,14-dimethylheptadeca-6,10,14-trienoate (**19**)

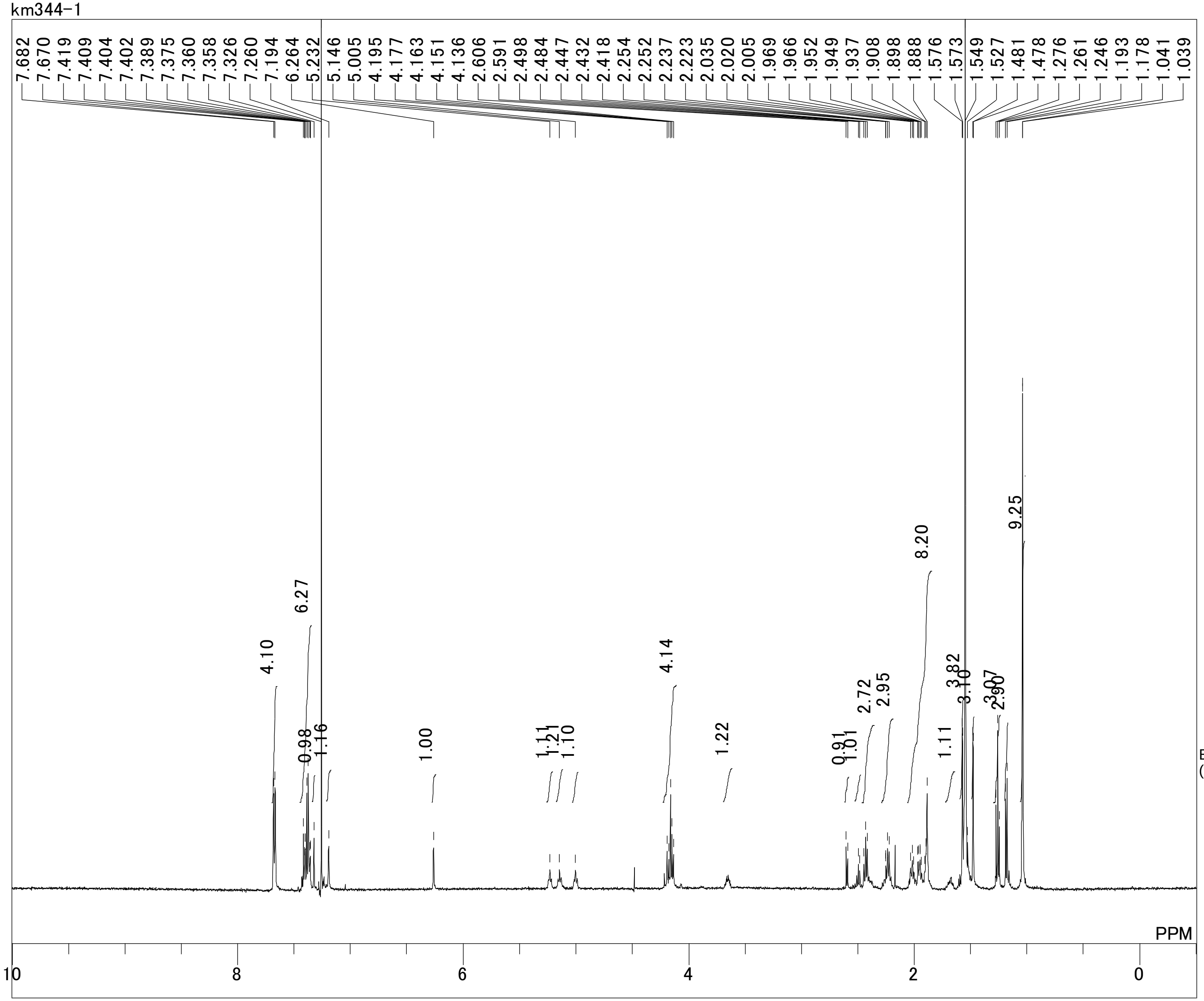

DFILE F:¥mameda¥NMR¥km344-1.als
COMNT km344-1
DATIM Sat Jul 30 10:03:39 2011
OBNUC 1H
EXMOD non
OBFRQ 500.00 MHz
OBSET 0.00 KHz
OBFIN 162160.00 Hz
POINT 8192
FREQU 10000.00 Hz
SCANS 8
ACQTM 0.8192 sec
PD 6.1808 sec
PW1 6.20 usec
IRNUC 1H
CTEMP 25.9 c
SLVNT CDCL3
EXREF 7.26 ppm
BF 0.12 Hz
RGAIN 26

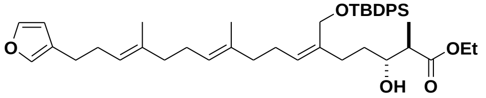

Ethyl (2*R**,3*R**,6*Z*,10*E*,14*E*)-6-(*tert*-butyldiphenylsiloxy)methyl-17-(furan-3-yl)-3-hydroxy-2,10,14-trimethylheptadeca-6,10,14-trienoate (**20**)

km344-1(13C)

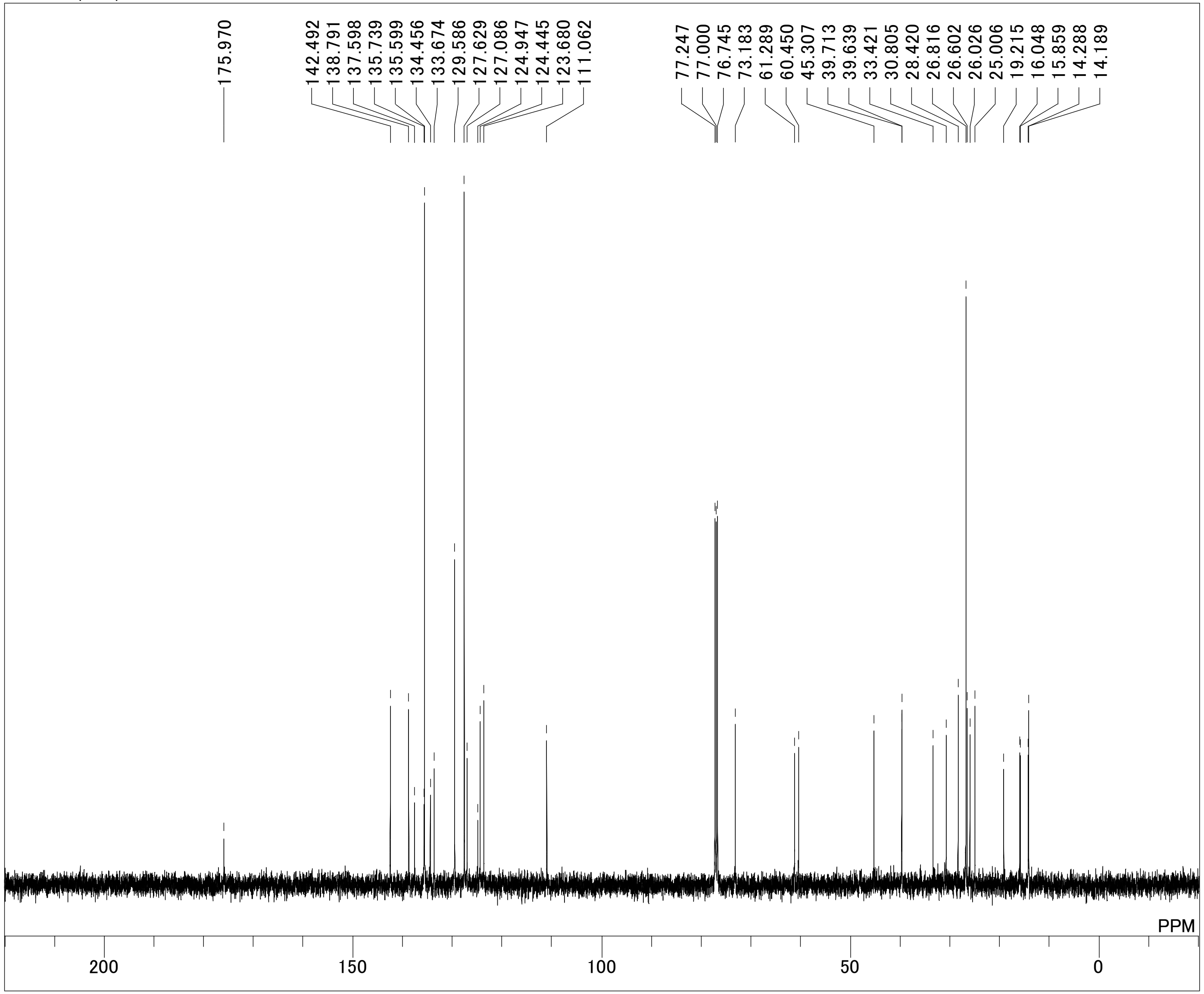

DFILE	F:¥mameda¥NMR¥km344-1(13C).als
COMNT	km344-1(13C)
DATIM	Sat Jul 30 10:26:44 2011
OBNUC	13C
EXMOD	bcm
OBFRQ	125.65 MHz
OBSET	0.00 KHz
OBFIN	127958.00 Hz
POINT	32768
FREQU	33898.30 Hz
SCANS	192
ACQTM	0.9667 sec
PD	2.0333 sec
PW1	4.90 usec
IRNUC	1H
CTEMP	27.7 c
SLVNT	CDCL3
EXREF	77.00 ppm
BF	1.20 Hz
RGAIN	30

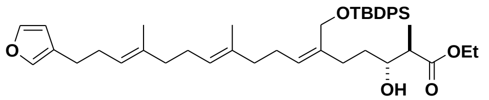

Ethyl (2*R**,3*R**,6*Z*,10*E*,14*E*)-6-(*tert*-butyldiphenylsiloxy)methyl-17-(furan-3-yl)-3-hydroxy-2,10,14-trimethylheptadeca-6,10,14-trienoate (**20**)

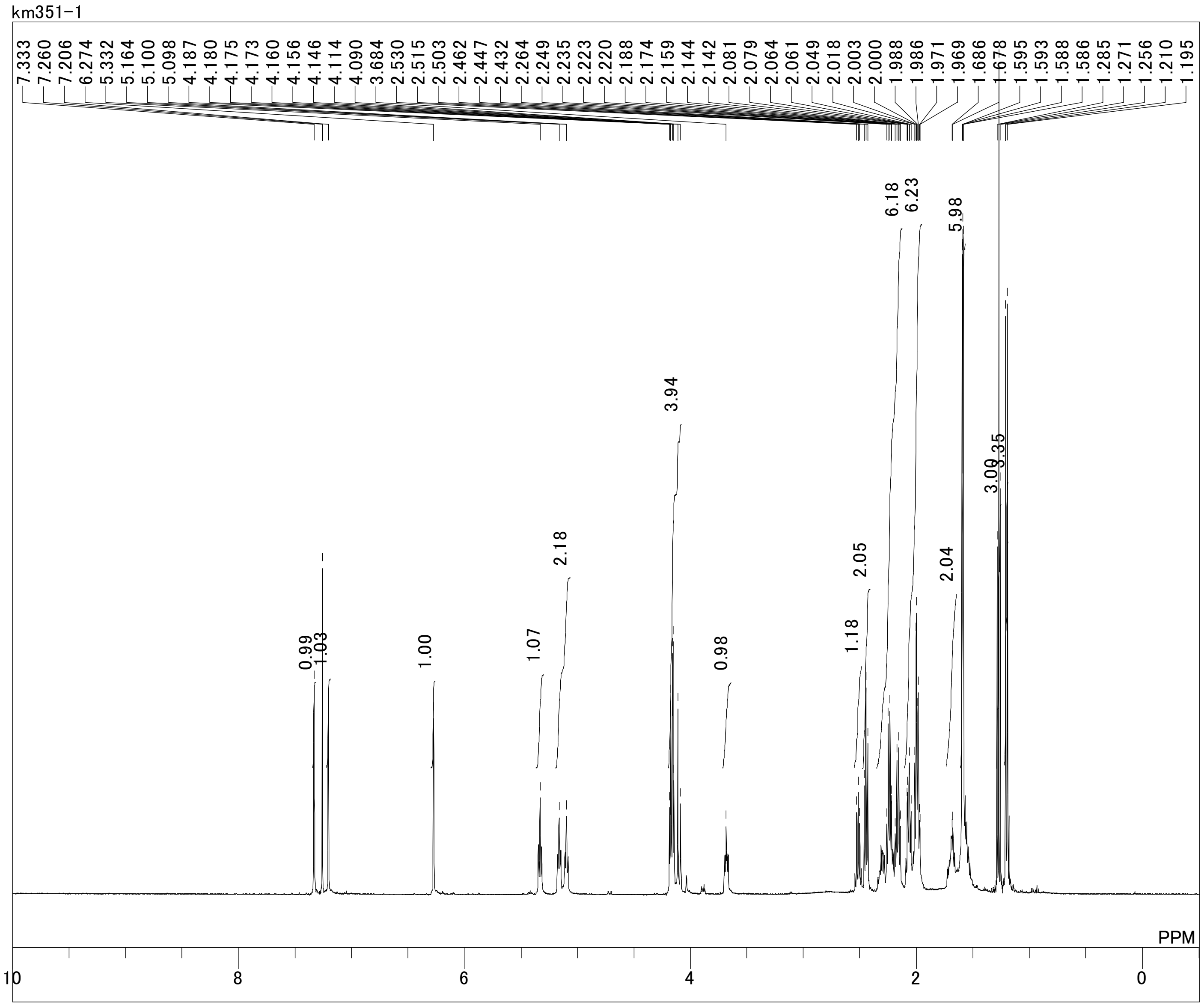

DFILE	F:¥mameda¥NMR¥km351-1.als
COMNT	km351-1
DATIM	Thu Aug 04 14:43:39 2011
OBNUC	1H
EXMOD	non
OBFRQ	500.00 MHz
OBSET	0.00 KHz
OBFIN	162160.00 Hz
POINT	8192
FREQU	10000.00 Hz
SCANS	8
ACQTM	0.8192 sec
PD	6.1808 sec
PW1	6.20 usec
IRNUC	1H
CTEMP	26.7 c
SLVNT	CDCL3
EXREF	7.26 ppm
BF	0.12 Hz
RGAIN	19

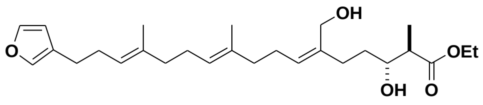

Ethyl (2*R**,3*R**,6*Z*,10*E*,14*E*)-17-(furan-3-yl)-3-hydroxy-6-(hydroxymethyl)-2,10,14-trimethylheptadeca-6,10,14-trienoate (**21**)

km351-1(13C)

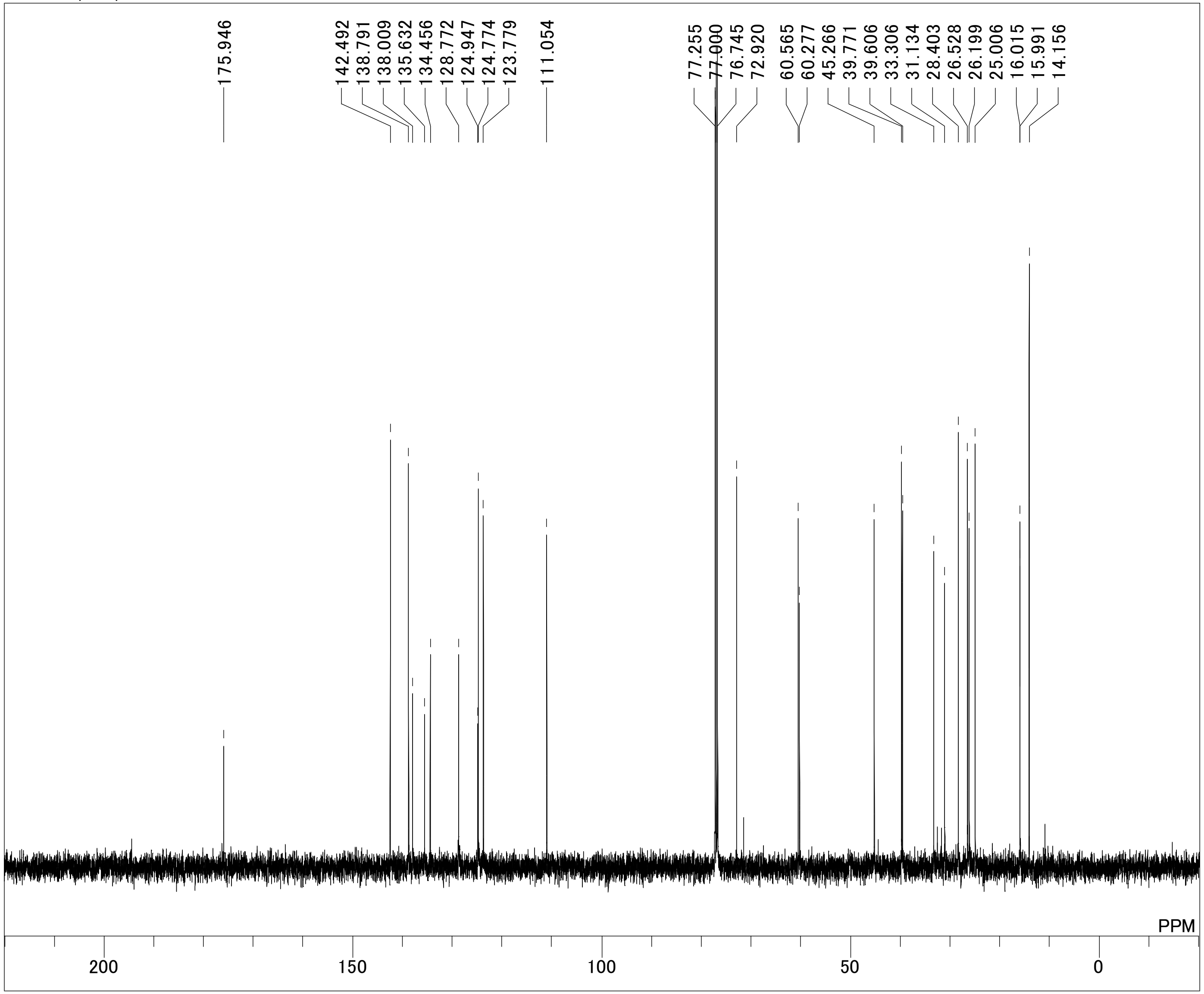

DFILE	F:¥mameda¥NMR¥km351-1(13C).als
COMNT	km351-1(13C)
DATIM	Thu Aug 04 15:37:55 2011
OBNUC	13C
EXMOD	bcm
OBFRQ	125.65 MHz
OBSET	0.00 KHz
OBFIN	127958.00 Hz
POINT	32768
FREQU	33898.30 Hz
SCANS	384
ACQTM	0.9667 sec
PD	2.0333 sec
PW1	4.90 usec
IRNUC	1H
CTEMP	28.9 c
SLVNT	CDCL3
EXREF	77.00 ppm
BF	1.20 Hz
RGAIN	31

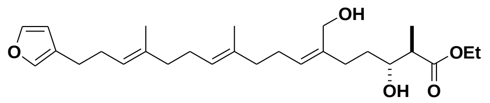

Ethyl (2*R**,3*R**,6*Z*,10*E*,14*E*)-17-(furan-3-yl)-3-hydroxy-6-(hydroxymethyl)-2,10,14-trimethylheptadeca-6,10,14-trienoate (**21**)

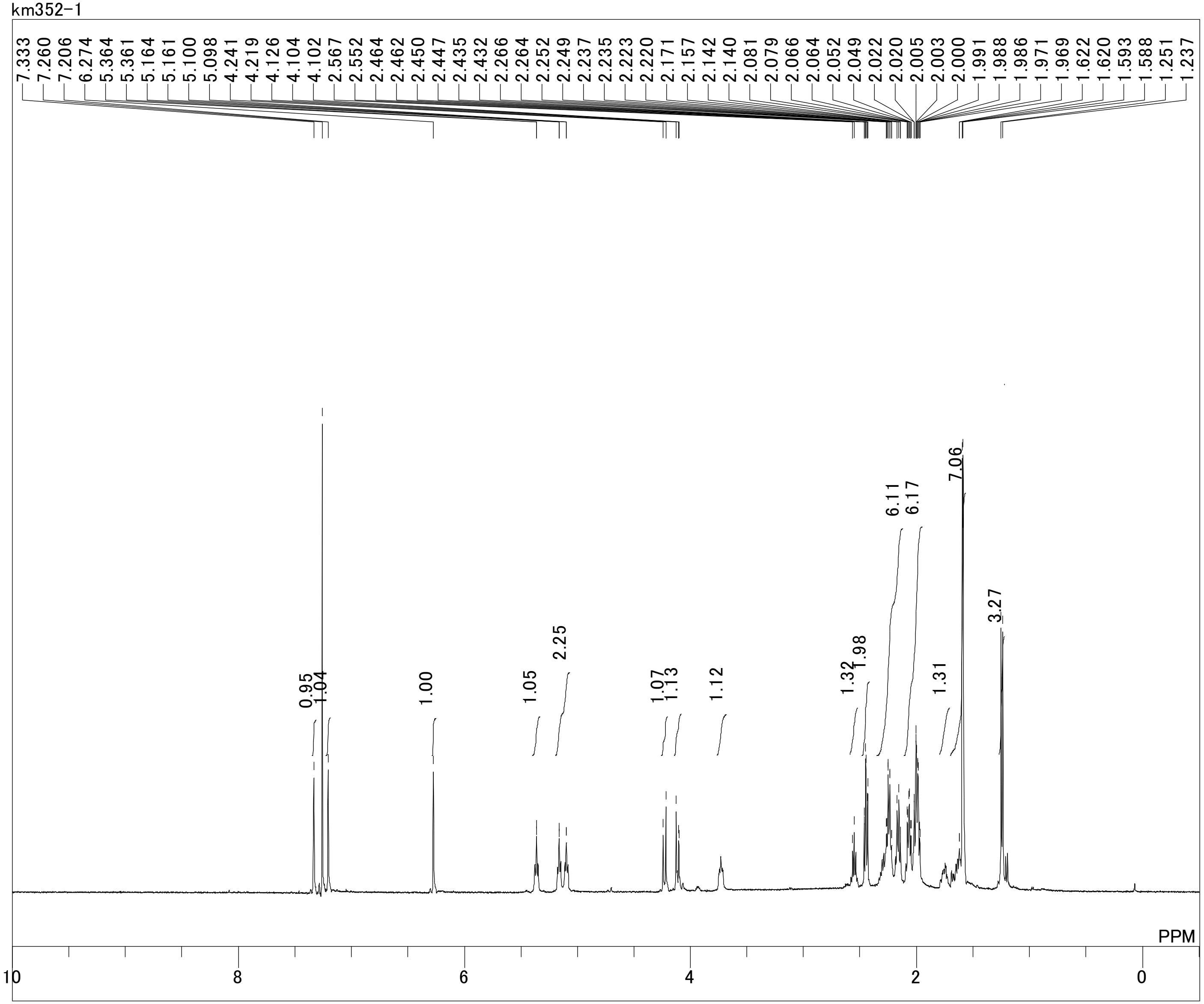

DFILE F:¥mameda¥NMR¥km352-1.als
COMNT km352-1
DATIM Fri Aug 05 10:39:23 2011
OBNUC 1H
EXMOD non
OBFRQ 500.00 MHz
OBSET 0.00 KHz
OBFIN 162160.00 Hz
POINT 8192
FREQU 10000.00 Hz
SCANS 8
ACQTM 0.8192 sec
PD 6.1808 sec
PW1 6.20 usec
IRNUC 1H
CTEMP 26.5 c
SLVNT CDCL3
EXREF 7.26 ppm
BF 0.12 Hz
RGAIN 22

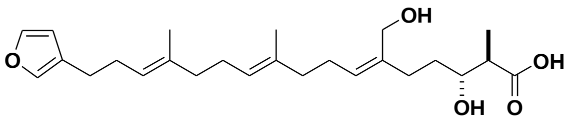

(2*R**,3*R**,6*Z*,10*E*,14*E*)-17-(Furan-3-yl)-3-hydroxy-6-(hydroxymethyl)-2,10,14-trimethylheptadeca-6,10,14-trienoic acid (**2**)

km352-1(13C)

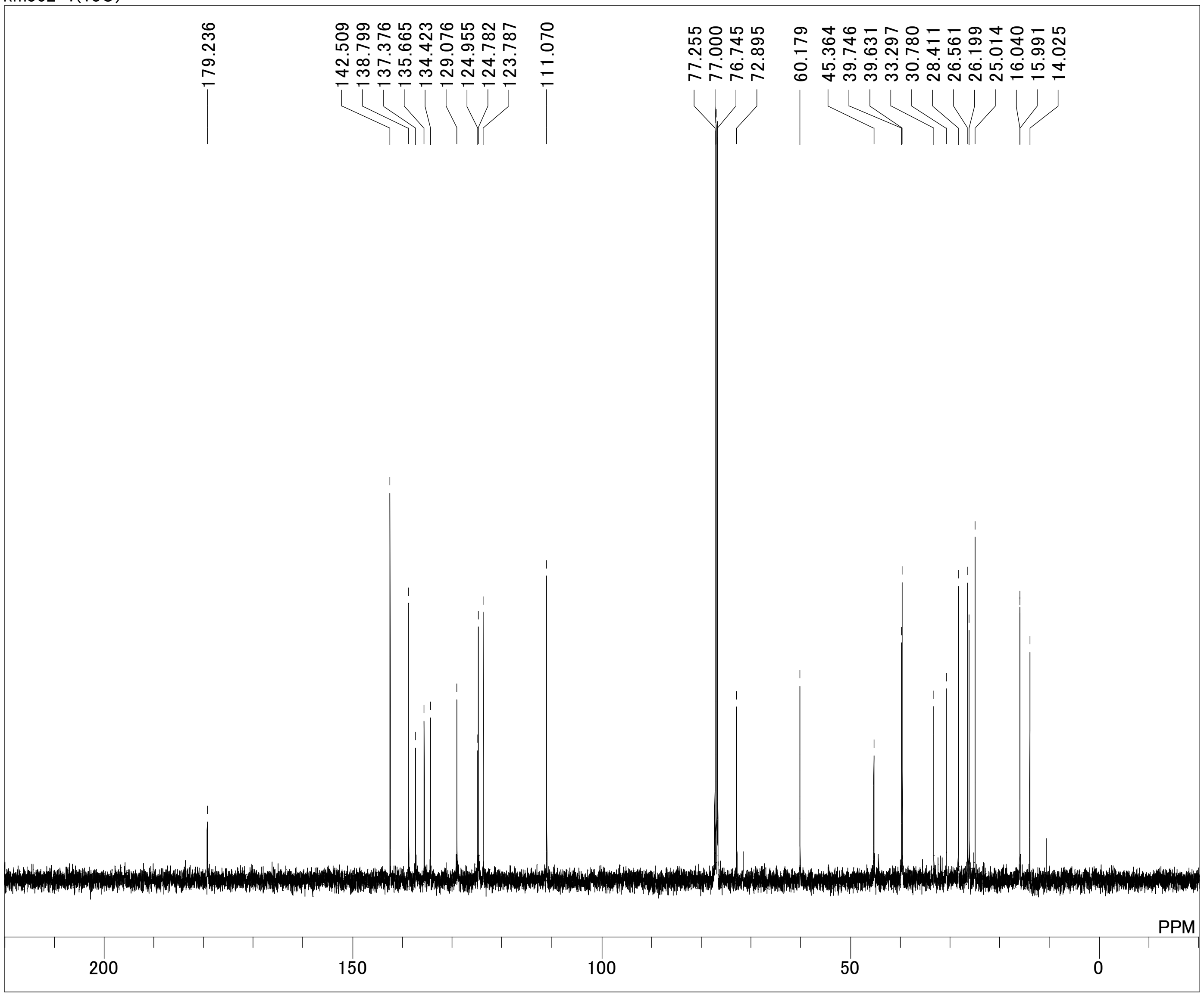

DFILE	F:¥mameda¥NMR¥km352-1(13C).als
COMNT	km352-1(13C)
DATIM	Fri Aug 05 11:19:43 2011
OBNUC	13C
EXMOD	bcm
OBFRQ	125.65 MHz
OBSET	0.00 KHz
OBFIN	127958.00 Hz
POINT	32768
FREQU	33898.30 Hz
SCANS	640
ACQTM	0.9667 sec
PD	2.0333 sec
PW1	4.90 usec
IRNUC	1H
CTEMP	28.9 c
SLVNT	CDCL3
EXREF	77.00 ppm
BF	1.20 Hz
RGAIN	31

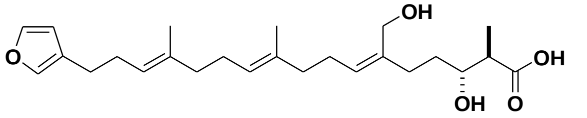

(2*R**,3*R**,6*Z*,10*E*,14*E*)-17-(Furan-3-yl)-3-hydroxy-6-(hydroxymethyl)-2,10,14-trimethylheptadeca-6,10,14-trienoic acid (**2**)

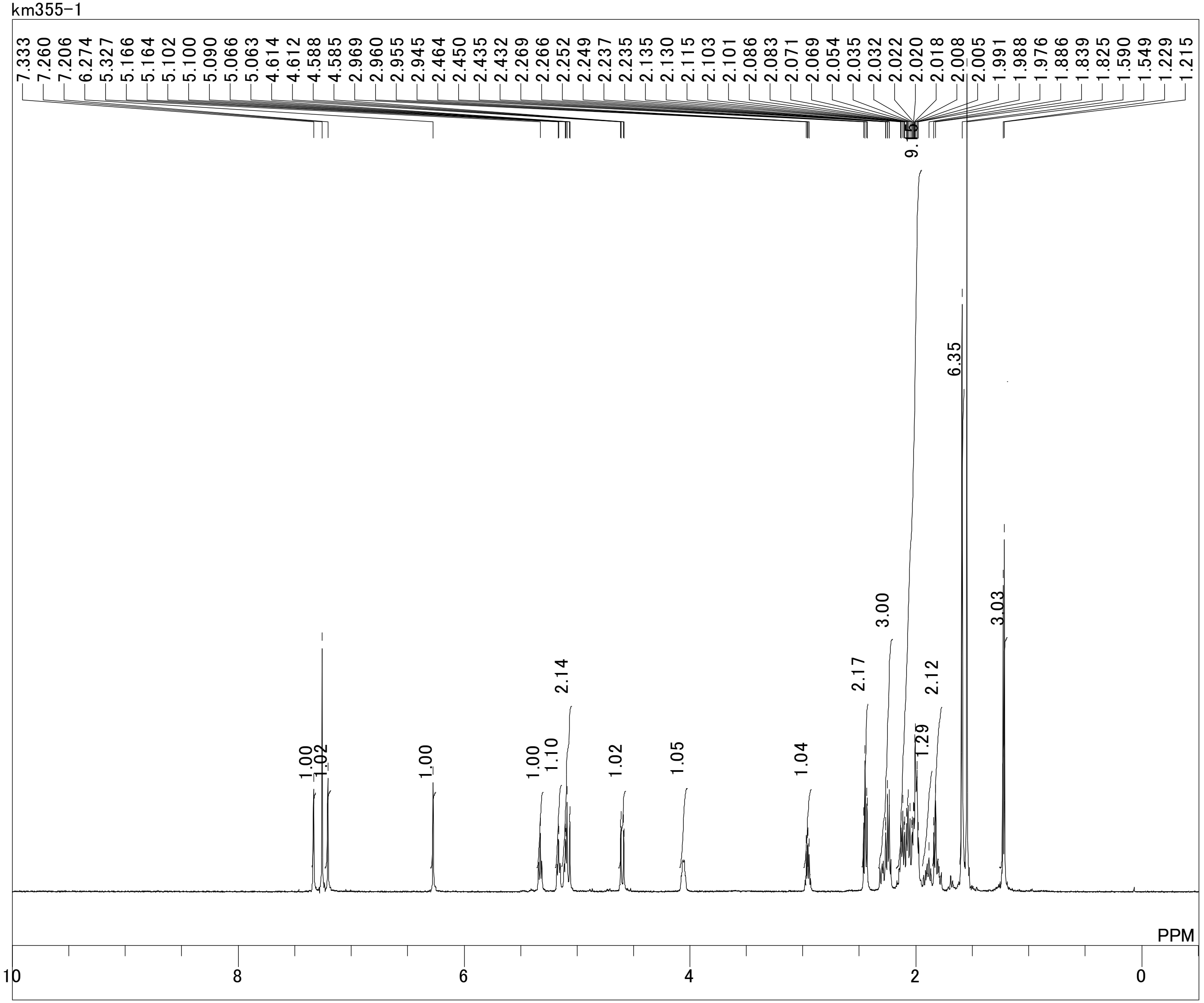

DFILE F:¥mameda¥NMR¥km355-1.als
COMNT km355-1
DATIM Sat Aug 06 16:42:52 2011
OBNUC 1H
EXMOD non
OBFRQ 500.00 MHz
OBSET 0.00 KHz
OBFIN 162160.00 Hz
POINT 8192
FREQU 10000.00 Hz
SCANS 8
ACQTM 0.8192 sec
PD 6.1808 sec
PW1 6.20 usec
IRNUC 1H
CTEMP 27.2 c
SLVNT CDCL3
EXREF 7.26 ppm
BF 0.12 Hz
RGAIN 22

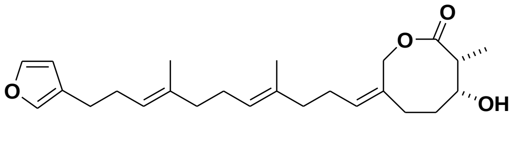

(2*R**,3*R**,6*Z*)-6-((4*E*,8*E*)-11-(Furan-3-yl)-4,8-dimethylundeca-4,8-dien-1-ylidene)-3-hydroxy-2-methylheptan-7-olide (**1**)

km355-1(13C)

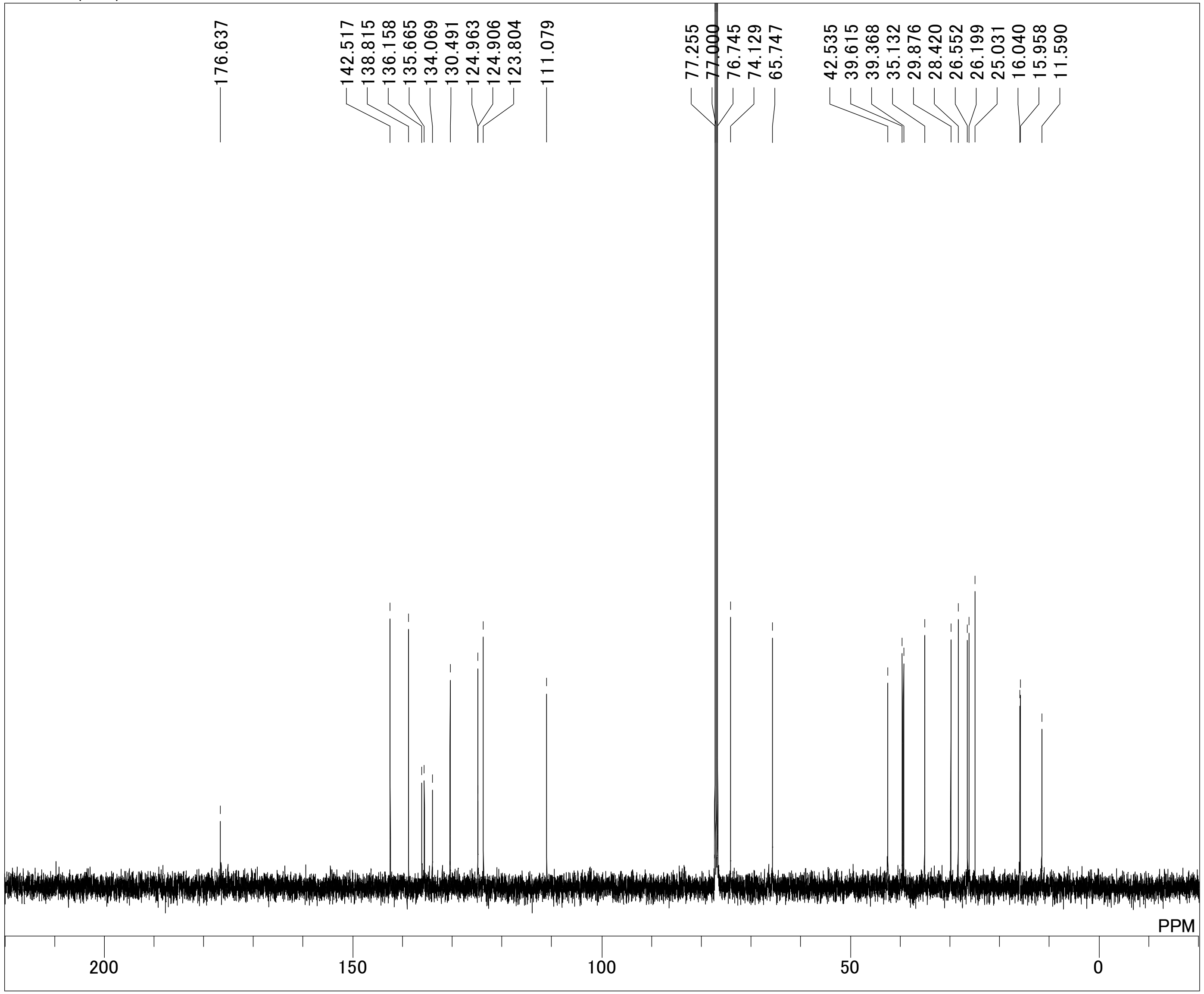

DFILE	F:¥mameda¥NMR¥km355-1(13C).als
COMNT	km355-1(13C)
DATIM	Sat Aug 06 17:19:18 2011
OBNUC	13C
EXMOD	bcm
OBFRQ	125.65 MHz
OBSET	0.00 KHz
OBFIN	127958.00 Hz
POINT	32768
FREQU	33898.30 Hz
SCANS	640
ACQTM	0.9667 sec
PD	2.0333 sec
PW1	4.90 usec
IRNUC	1H
CTEMP	29.3 c
SLVNT	CDCL3
EXREF	77.00 ppm
BF	1.20 Hz
RGAIN	31

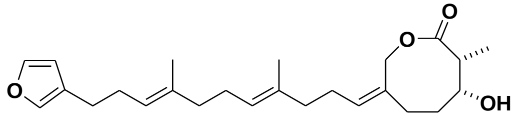

(2*R**,3*R**,6*Z*)-6-((4*E*,8*E*)-11-(Furan-3-yl)-4,8-dimethylundeca-4,8-dien-1-ylidene)-3-hydroxy-2-methylheptan-7-olide (**1**)

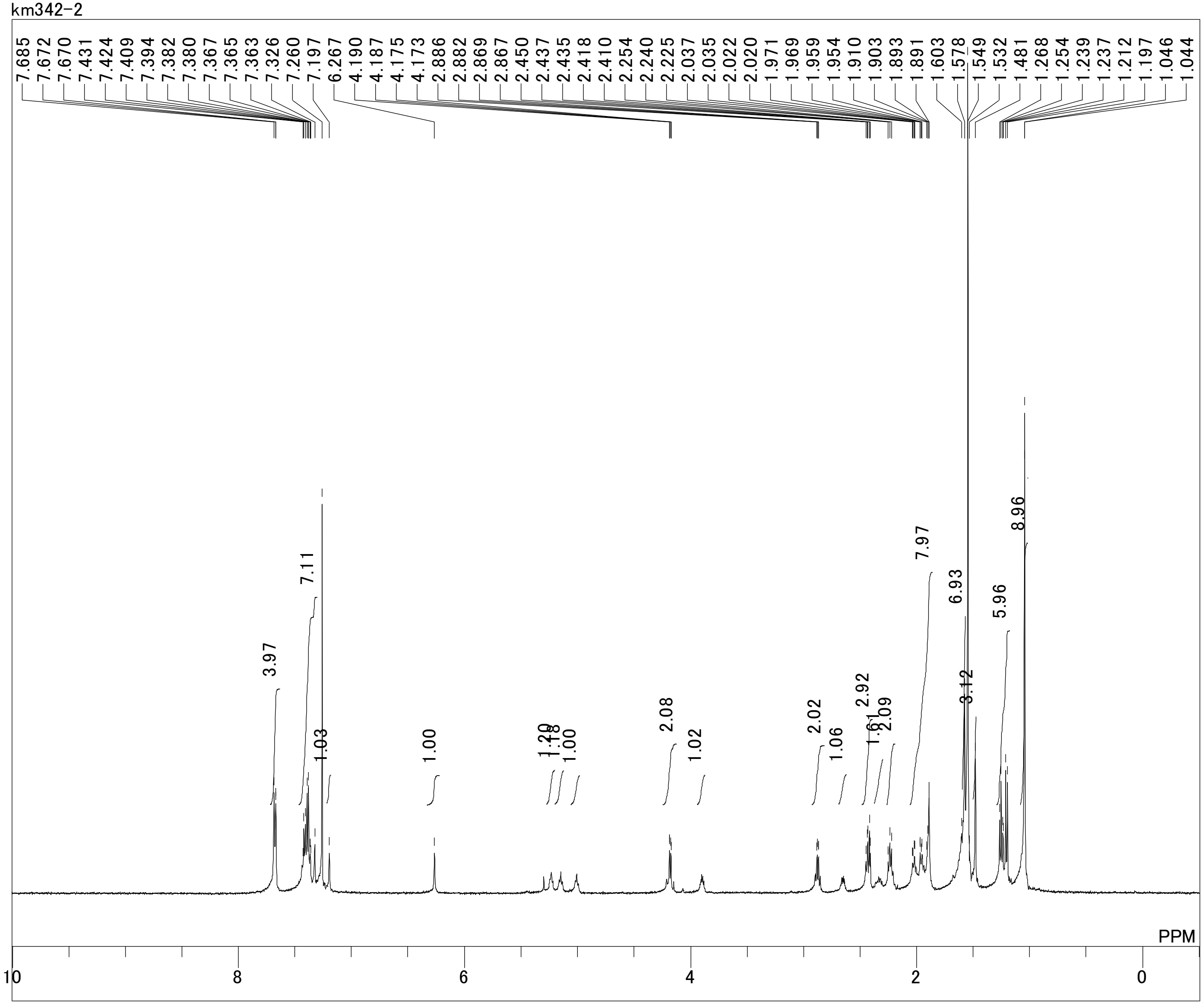

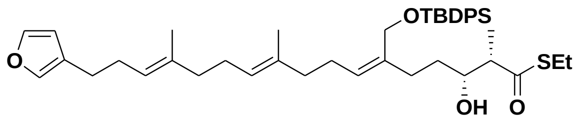

S-Ethyl (2*S*,3*R*,6*Z*,10*E*,14*E*)-6-((*tert*-butyldiphenylsiloxy)methyl)-17-(furan-3-yl)-3-hydroxy-2,10,14-trimethylheptadeca-6,10,14-trienethioate (**24**)

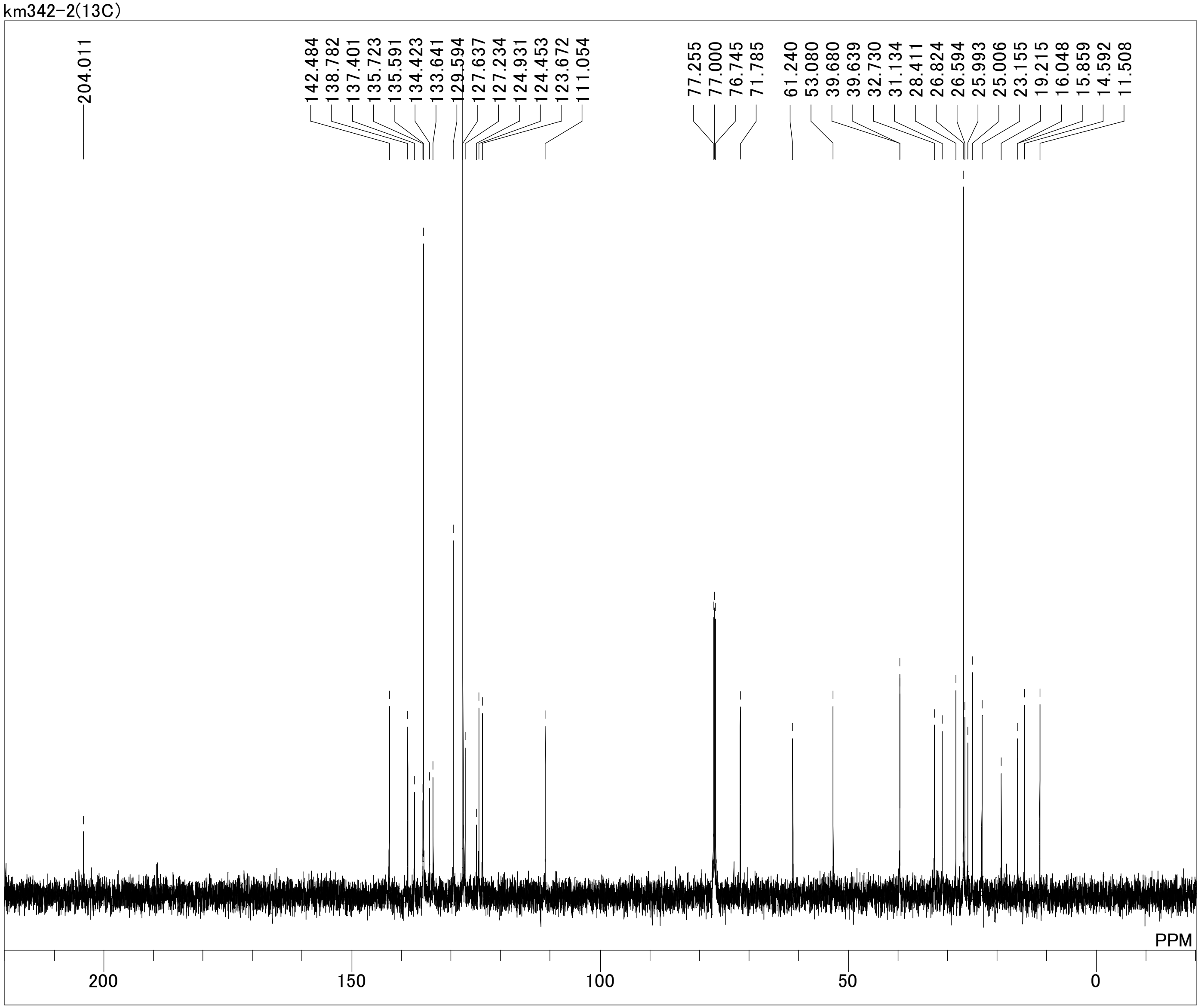

DFILE F:¥mameda¥NMR¥km342-2(13C).als
COMNT km342-2(13C)
DATIM Sat Jul 23 14:34:40 2011
OBNUC 13C
EXMOD bcm
OBFRQ 125.65 MHz
OBSET 0.00 KHz
OBFIN 127958.00 Hz
POINT 32768
FREQU 33898.30 Hz
SCANS 64
ACQTM 0.9667 sec
PD 2.0333 sec
PW1 4.90 usec
IRNUC 1H
CTEMP 27.2 c
SLVNT CDCL3
EXREF 77.00 ppm
BF 1.20 Hz
RGAIN 31

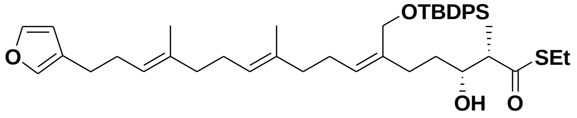

S-Ethyl (2*S*,3*R*,6*Z*,10*E*,14*E*)-6-((*tert*-butyldiphenylsiloxy)methyl)-17-(furan-3-yl)-3-hydroxy-2,10,14-trimethylheptadeca-6,10,14-trienethioate (**24**)

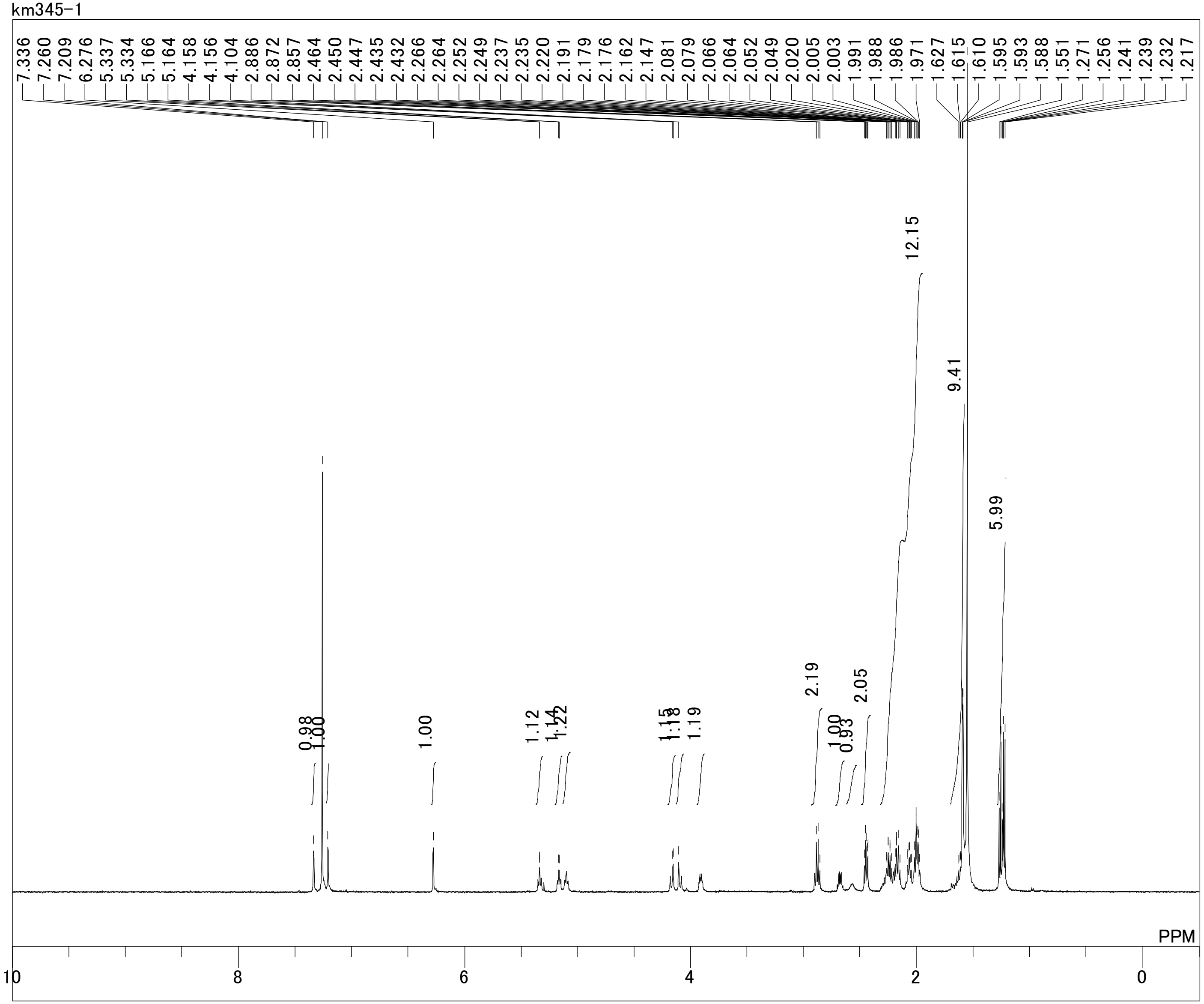

DFILE F:¥mameda¥NMR¥km345-1.als
COMNT km345-1
DATIM Thu Jul 28 10:38:44 2011
OBNUC 1H
EXMOD non
OBFRQ 500.00 MHz
OBSET 0.00 KHz
OBFIN 162160.00 Hz
POINT 8192
FREQU 10000.00 Hz
SCANS 8
ACQTM 0.8192 sec
PD 6.1808 sec
PW1 6.20 usec
IRNUC 1H
CTEMP 26.1 c
SLVNT CDCL3
EXREF 7.26 ppm
BF 0.12 Hz
RGAIN 24

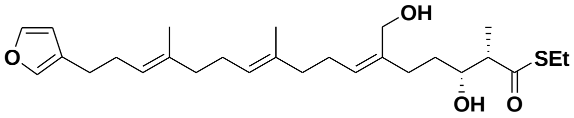

S-Ethyl (2*S*,3*R*,6*Z*,10*E*,14*E*)-17-(furan-3-yl)-3-hydroxy-6-(hydroxymethyl)-2,10,14-trimethylheptadeca-6,10,14-trienethioate (**25**)

km345-1(13C)

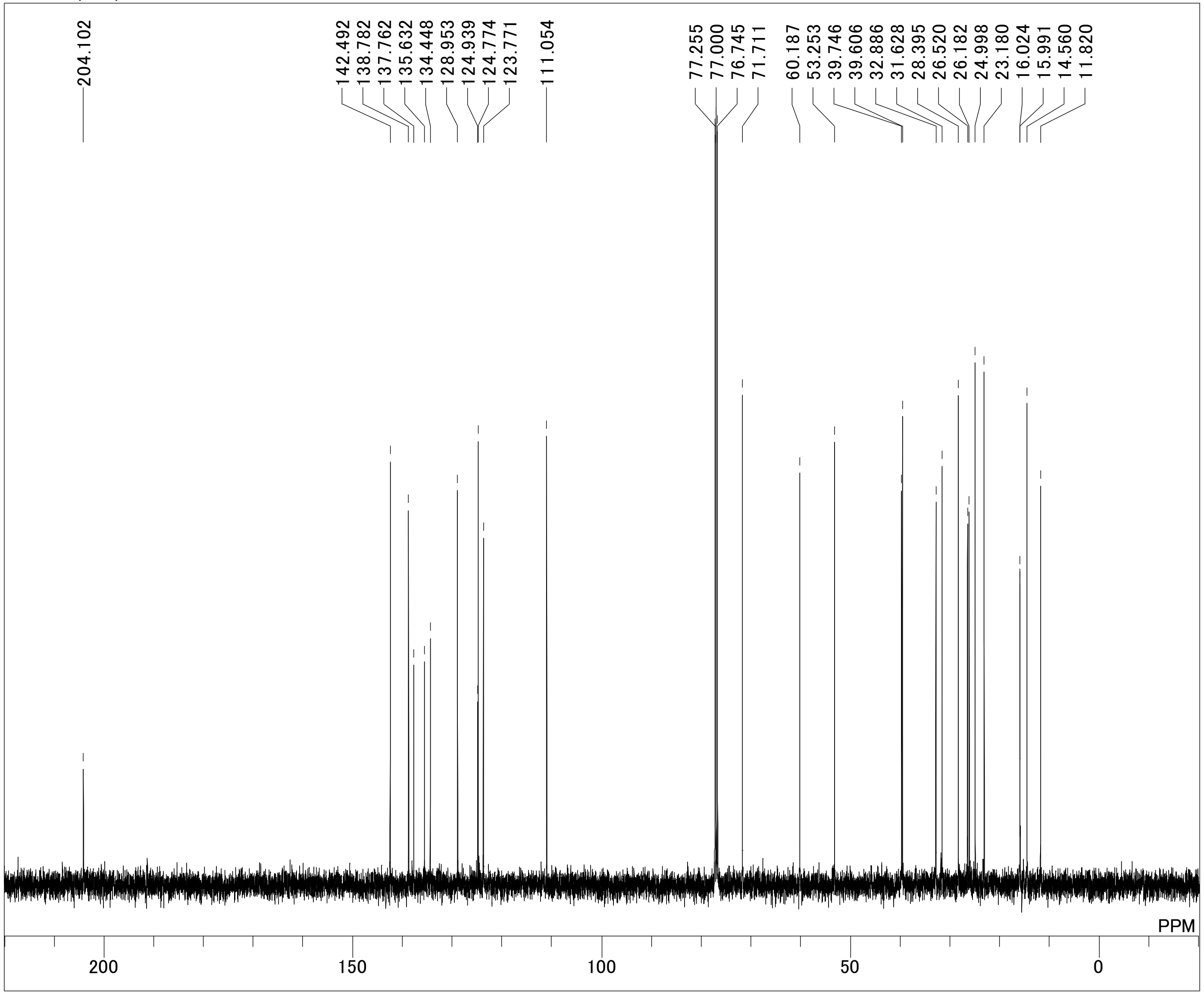

DFILE F:¥mameda¥NMR¥km345-1(13C).als
COMNT km345-1(13C)
DATIM Thu Jul 28 11:01:26 2011
OBNUC 13C
EXMOD bcm
OBFRQ 125.65 MHz
OBSET 0.00 KHz
OBFIN 127958.00 Hz
POINT 32768
FREQU 33898.30 Hz
SCANS 256
ACQTM 0.9667 sec
PD 2.0333 sec
PW1 4.90 usec
IRNUC 1H
CTEMP 28.0 c
SLVNT CDCL3
EXREF 77.00 ppm
BF 1.20 Hz
RGAIN 30

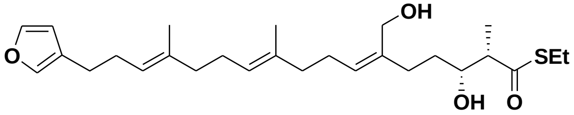

S-Ethyl (2*S*,3*R*,6*Z*,10*E*,14*E*)-17-(furan-3-yl)-3-hydroxy-6-(hydroxymethyl)-2,10,14-trimethylheptadeca-6,10,14-trienethioate (**25**)

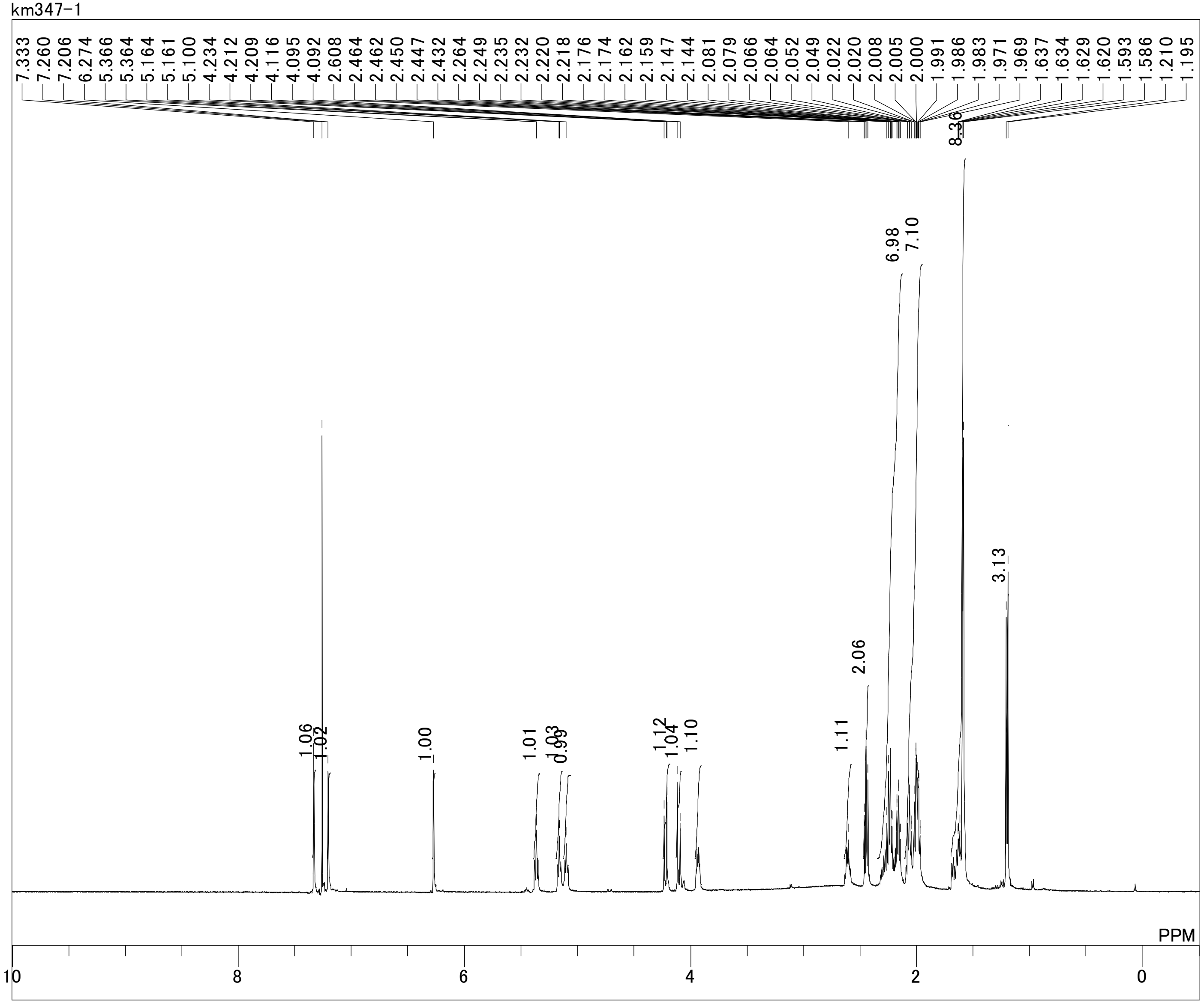

DFILE	F:¥mameda¥NMR¥km347-1.als
COMNT	km347-1
DATIM	Thu Jul 28 21:24:03 2011
OBNUC	1H
EXMOD	non
OBFRQ	500.00 MHz
OBSET	0.00 KHz
OBFIN	162160.00 Hz
POINT	8192
FREQU	10000.00 Hz
SCANS	8
ACQTM	0.8192 sec
PD	6.1808 sec
PW1	6.20 usec
IRNUC	1H
CTEMP	26.9 c
SLVNT	CDCL3
EXREF	7.26 ppm
BF	0.12 Hz
RGAIN	21

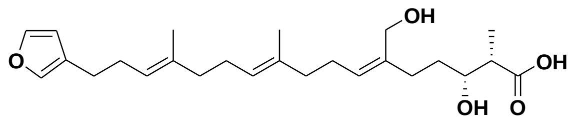

(2*S*,3*R*,6*Z*,10*E*,14*E*)-17-(Furan-3-yl)-3-hydroxy-6-(hydroxymethyl)-2,10,14-trimethylheptadeca-6,10,14-trienoic acid (**26**)

km347-1(13C)

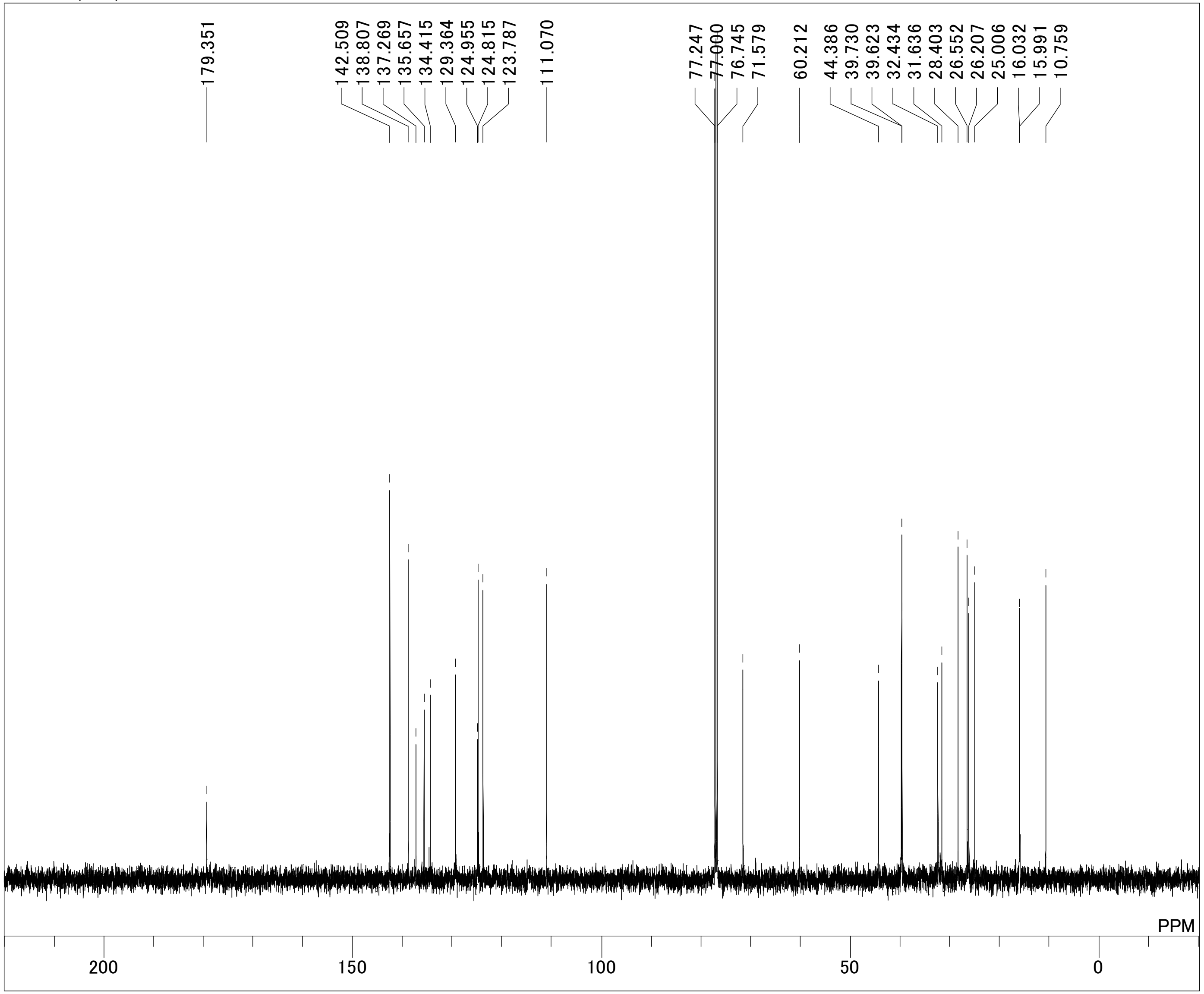

DFILE	F:¥mameda¥NMR¥km347-1(13C).als
COMNT	km347-1(13C)
DATIM	Thu Jul 28 21:55:14 2011
OBNUC	13C
EXMOD	bcm
OBFRQ	125.65 MHz
OBSET	0.00 KHz
OBFIN	127958.00 Hz
POINT	32768
FREQU	33898.30 Hz
SCANS	512
ACQTM	0.9667 sec
PD	2.0333 sec
PW1	4.90 usec
IRNUC	1H
CTEMP	28.6 c
SLVNT	CDCL3
EXREF	77.00 ppm
BF	1.20 Hz
RGAIN	31

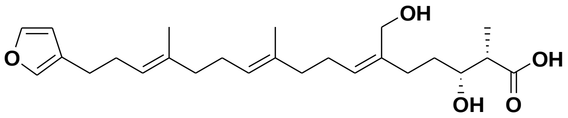

(2*S*,3*R*,6*Z*,10*E*,14*E*)-17-(Furan-3-yl)-3-hydroxy-6-(hydroxymethyl)-2,10,14-trimethylheptadeca-6,10,14-trienoic acid (**26**)

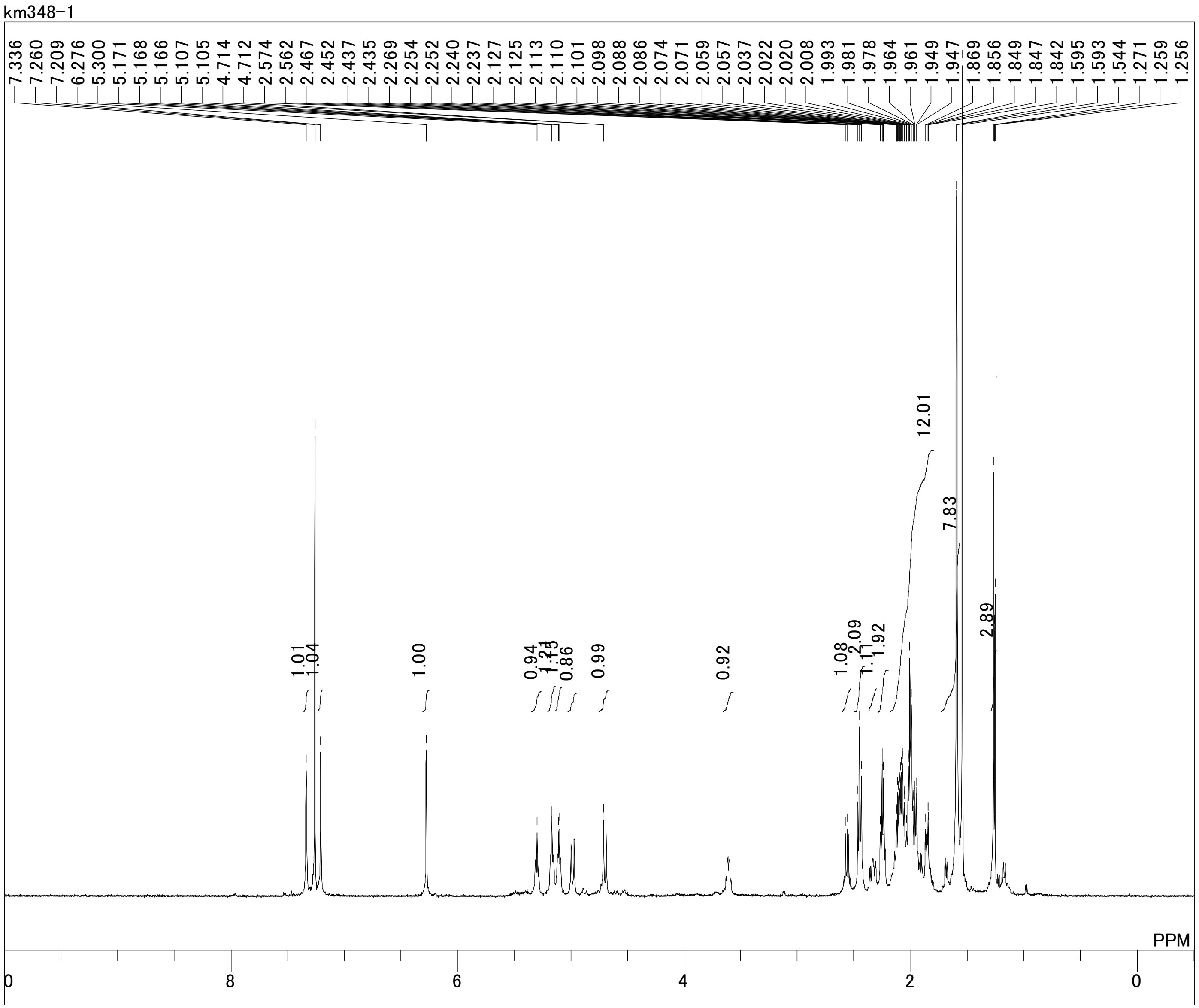

DFILE F:¥mameda¥NMR¥km348-1.als
COMNT km348-1
DATIM Sat Jul 30 16:41:26 2011
OBNUC 1H
EXMOD non
OBFRQ 500.00 MHz
OBSET 0.00 KHz
OBFIN 162160.00 Hz
POINT 8192
FREQU 10000.00 Hz
SCANS 8
ACQTM 0.8192 sec
PD 6.1808 sec
PW1 6.20 usec
IRNUC 1H
CTEMP 27.2 c
SLVNT CDCL3
EXREF 7.26 ppm
BF 0.12 Hz
RGAIN 23

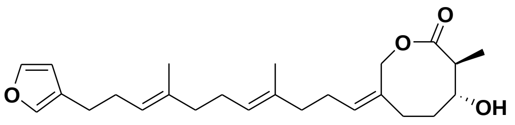

(2*S*,3*R*,6*Z*)-6-((4*E*,8*E*)-11-(furan-3-yl)-4,8-dimethylundeca-4,8-dien-1-ylidene)-3-hydroxy-2-methylheptan-7-olide (**1'**)

km348-1(13C)

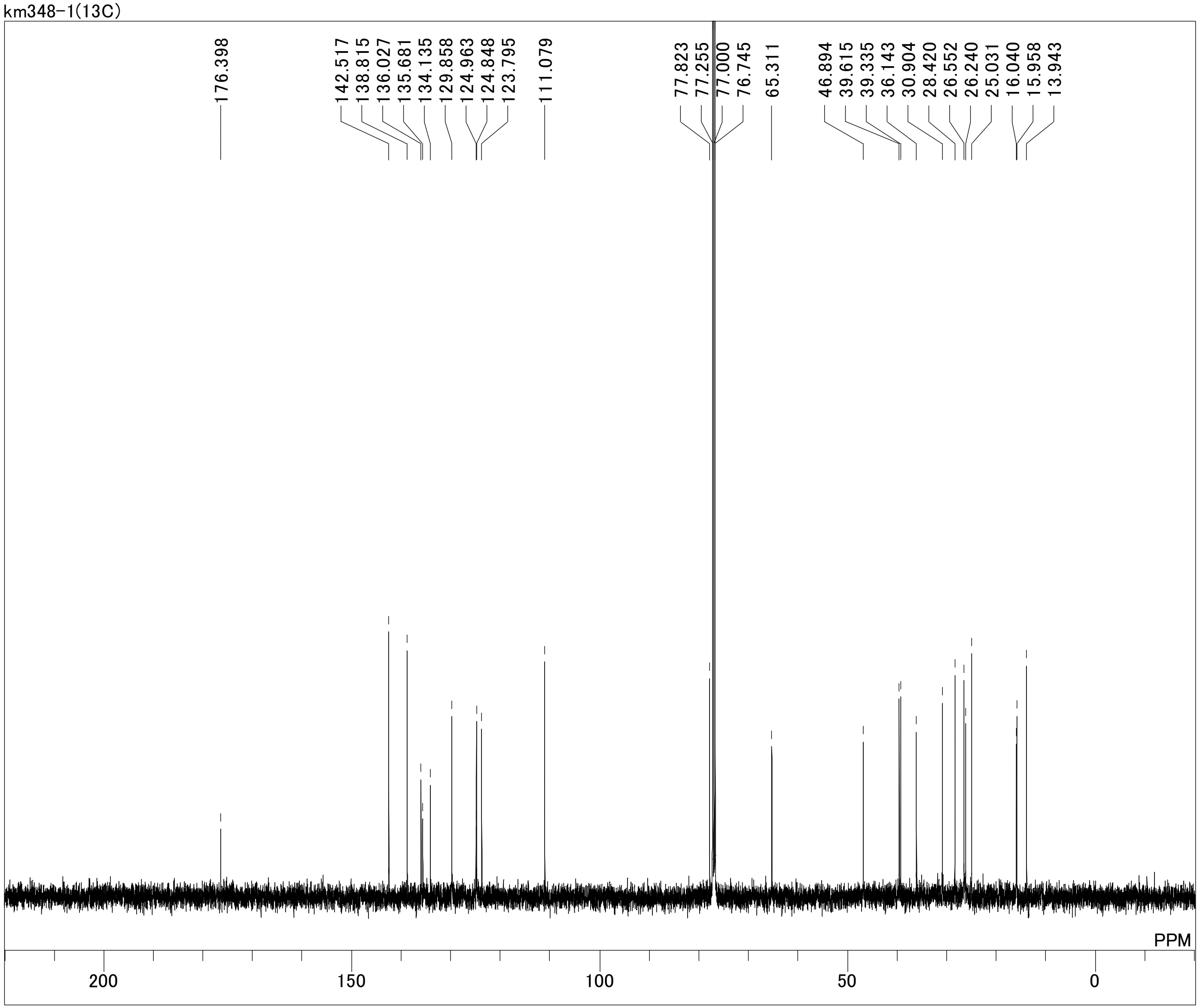

DFILE F:¥mameda¥NMR¥km348-1(13C).als
COMNT km348-1(13C)
DATIM Sat Jul 30 17:28:55 2011
OBNUC 13C
EXMOD bcm
OBFRQ 125.65 MHz
OBSET 0.00 KHz
OBFIN 127958.00 Hz
POINT 32768
FREQU 33898.30 Hz
SCANS 832
ACQTM 0.9667 sec
PD 2.0333 sec
PW1 4.90 usec
IRNUC 1H
CTEMP 28.7 c
SLVNT CDCL3
EXREF 77.00 ppm
BF 1.20 Hz
RGAIN 31

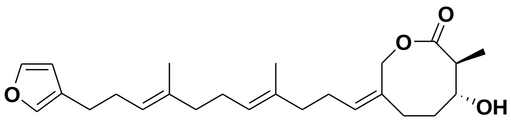

(2*S*,3*R*,6*Z*)-6-((4*E*,8*E*)-11-(furan-3-yl)-4,8-dimethylundeca-4,8-dien-1-ylidene)-3-hydroxy-2-methylheptan-7-olide (**1'**)

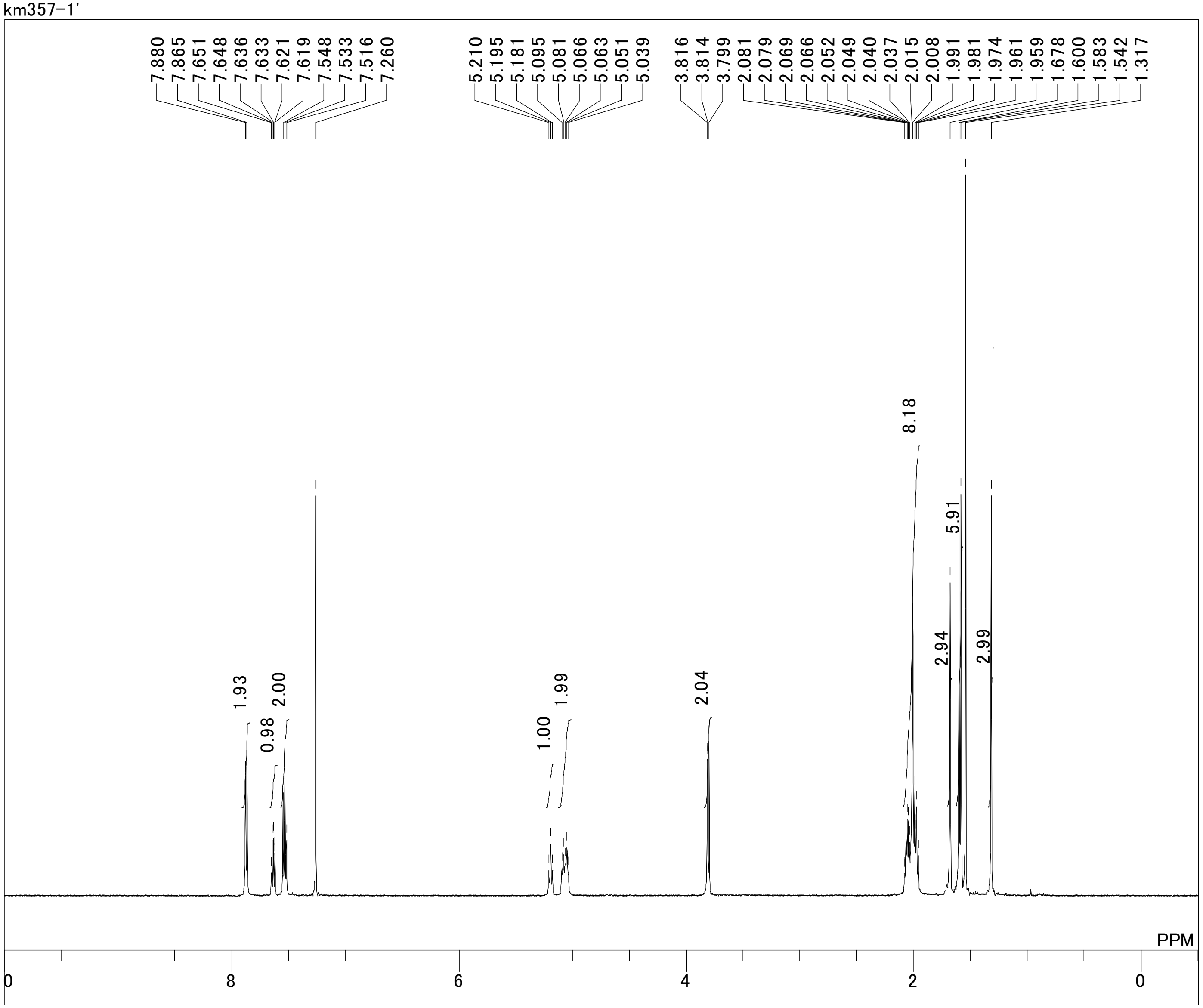

DFILE	F:¥mameda¥NMR¥km357-1'.als
COMNT	km357-1'
DATIM	Fri Sep 02 19:08:28 2011
OBNUC	1H
EXMOD	non
OBFRQ	500.00 MHz
OBSET	0.00 KHz
OBFIN	162160.00 Hz
POINT	8192
FREQU	10000.00 Hz
SCANS	8
ACQTM	0.8192 sec
PD	6.1808 sec
PW1	6.20 usec
IRNUC	1H
CTEMP	27.2 c
SLVNT	CDCL3
EXREF	7.26 ppm
BF	0.12 Hz
RGAIN	24

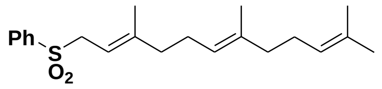

((2*E*,6*E*)-3,7,11-Trimethyldodeca-2,6,10-trien-1-yl)benzenesulfonate

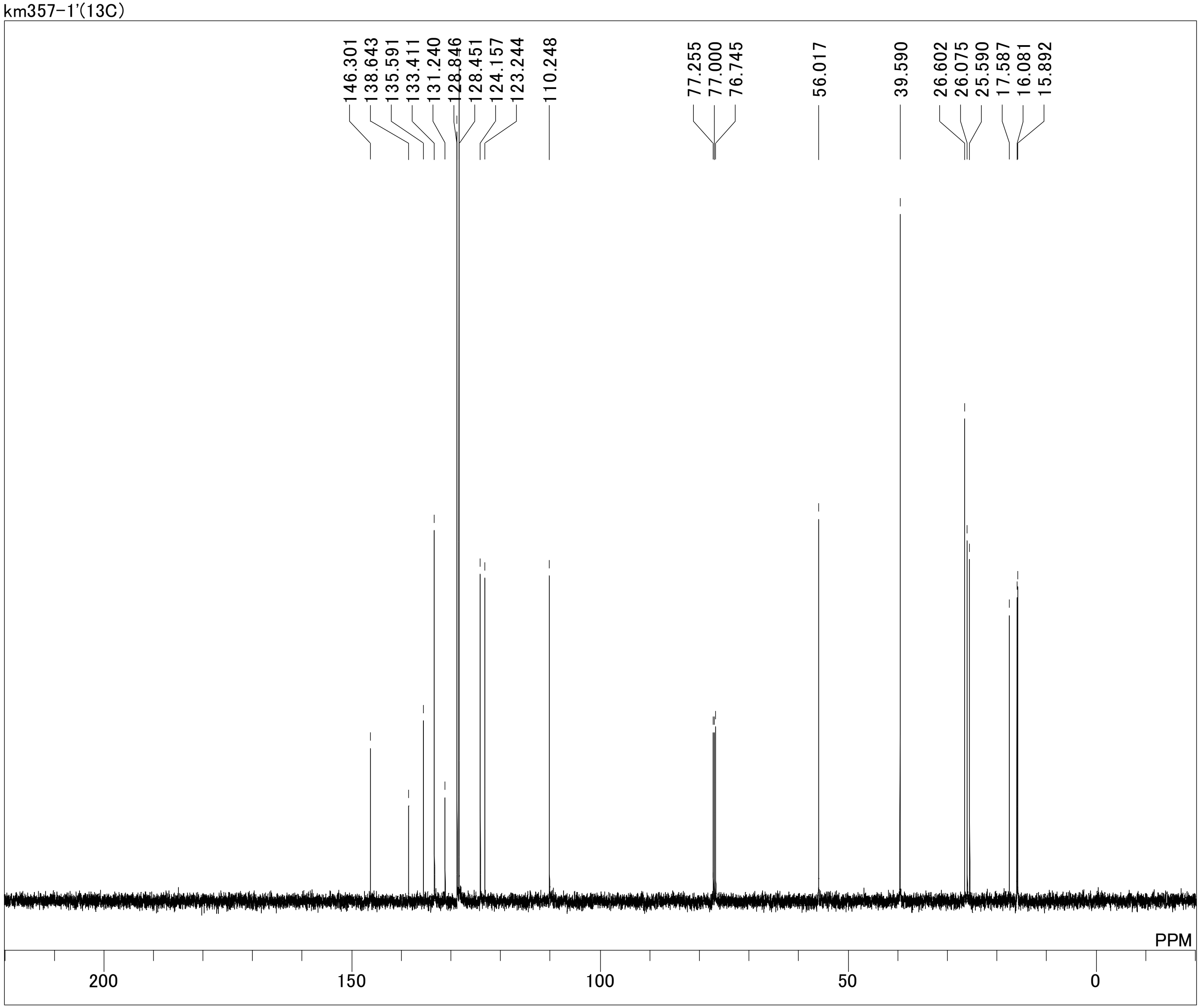

DFILE	F:¥mameda¥NMR¥km357-1'(13C).als
COMNT	km357-1'(13C)
DATIM	Fri Sep 02 19:24:39 2011
OBNUC	13C
EXMOD	bcm
OBFRQ	125.65 MHz
OBSET	0.00 KHz
OBFIN	127958.00 Hz
POINT	32768
FREQU	33898.30 Hz
SCANS	64
ACQTM	0.9667 sec
PD	2.0333 sec
PW1	4.90 usec
IRNUC	1H
CTEMP	28.7 c
SLVNT	CDCL3
EXREF	77.00 ppm
BF	1.20 Hz
RGAIN	30

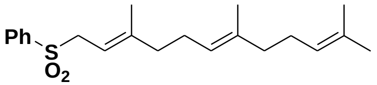

((2*E*,6*E*)-3,7,11-Trimethyldodeca-2,6,10-trien-1-yl)benzenesulfonate

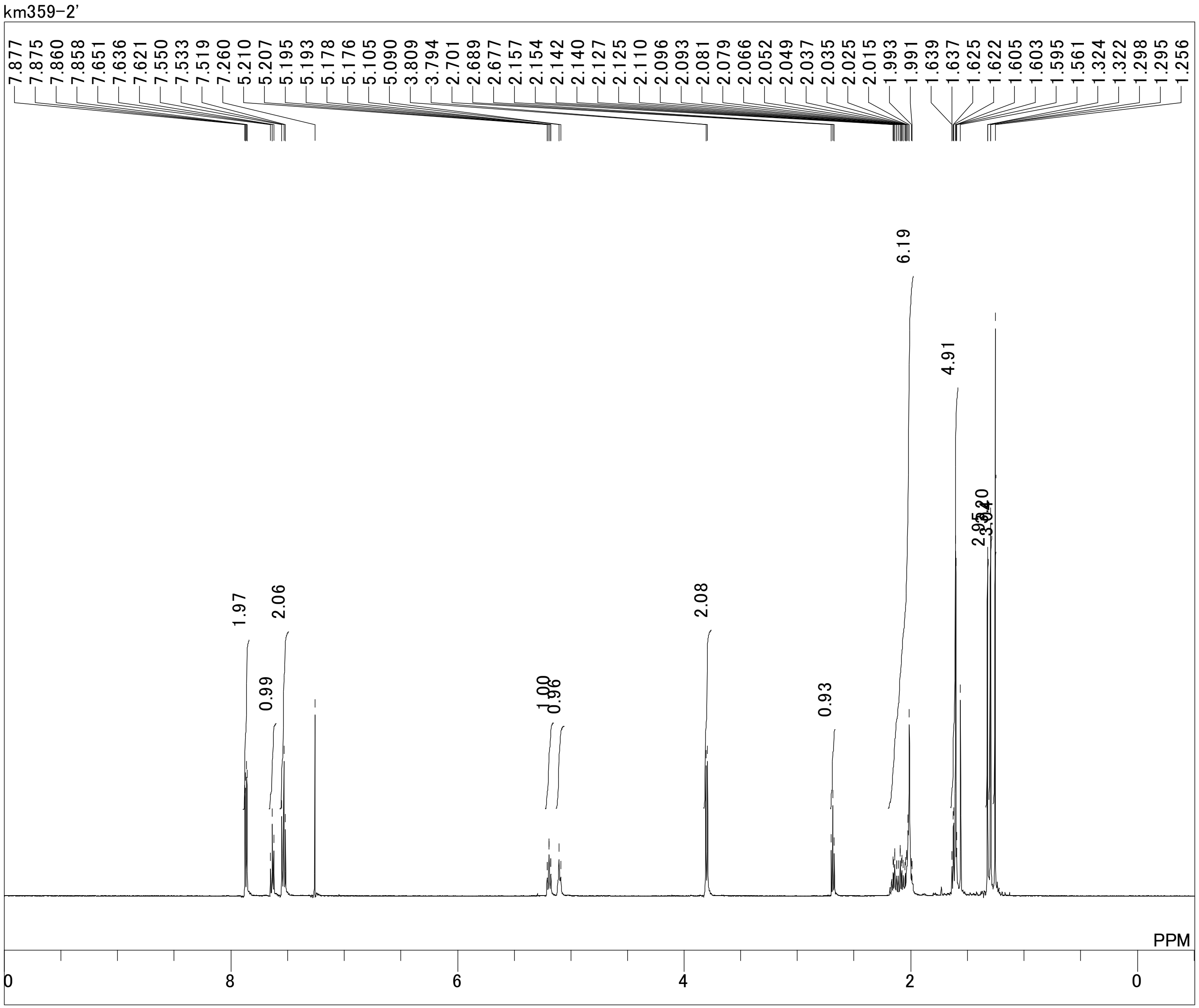

DFILE F:¥mameda¥NMR¥km358-1'.als
COMNT km359-2'
DATIM Tue Sep 13 11:50:21 2011
OBNUC 1H
EXMOD non
OBFRQ 500.00 MHz
OBSET 0.00 KHz
OBFIN 162160.00 Hz
POINT 8192
FREQU 10000.00 Hz
SCANS 8
ACQTM 0.8192 sec
PD 6.1808 sec
PW1 6.20 usec
IRNUC 1H
CTEMP 27.1 c
SLVNT CDCL3
EXREF 7.26 ppm
BF 0.12 Hz
RGAIN 20

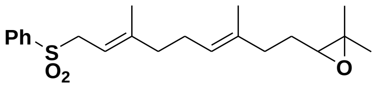

((2*E*,6*E*)-10,11-Epoxy-3,7,11-trimethyldodeca-2,6-dien-1-yl)benzenesulfonate

km359-2'(13C)

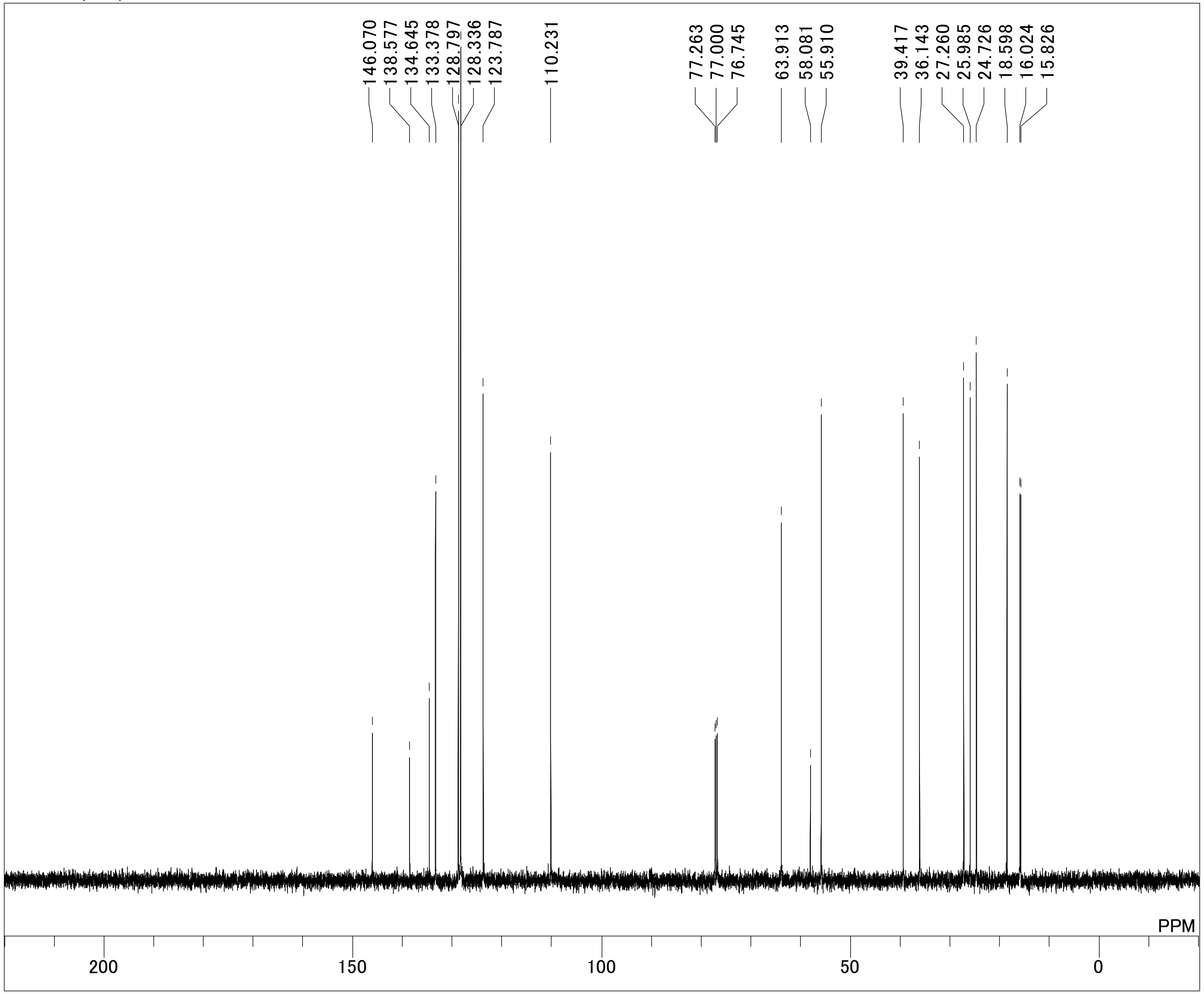

DFILE	F:¥mameda¥NMR¥km358-1'(13C).als
COMNT	km359-2'(13C)
DATIM	Tue Sep 13 11:56:07 2011
OBNUC	13C
EXMOD	bcm
OBFRQ	125.65 MHz
OBSET	0.00 KHz
OBFIN	127958.00 Hz
POINT	32768
FREQU	33898.30 Hz
SCANS	64
ACQTM	0.9667 sec
PD	2.0333 sec
PW1	4.90 usec
IRNUC	1H
CTEMP	28.2 c
SLVNT	CDCL3
EXREF	77.00 ppm
BF	1.20 Hz
RGAIN	31

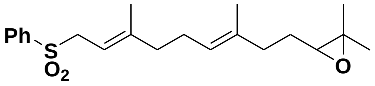

((2*E*,6*E*)-10,11-Epoxy-3,7,11-trimethyldodeca-2,6-dien-1-yl)benzenesulfonate

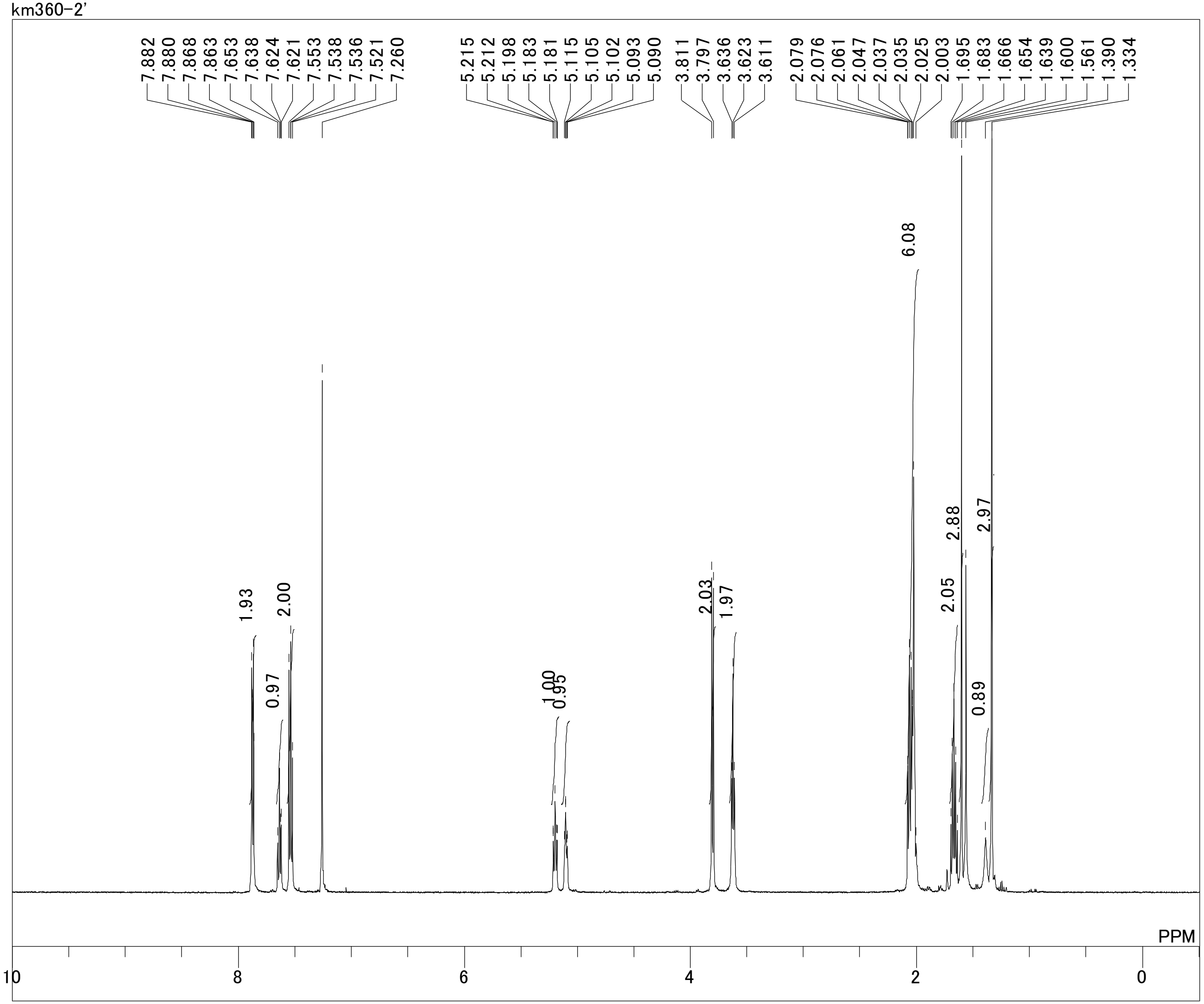

DFILE	F:¥mameda¥NMR¥km360-2'.als
COMNT	km360-2'
DATIM	Wed Sep 14 20:44:34 2011
OBNUC	1H
EXMOD	non
OBFRQ	500.00 MHz
OBSET	0.00 KHz
OBFIN	162160.00 Hz
POINT	8192
FREQU	10000.00 Hz
SCANS	8
ACQTM	0.8192 sec
PD	6.1808 sec
PW1	6.20 usec
IRNUC	1H
CTEMP	27.2 c
SLVNT	CDCL3
EXREF	7.26 ppm
BF	0.12 Hz
RGAIN	22

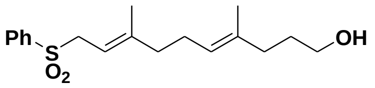

((2*E*,6*E*)-10-Hydroxy-3,7-dimethyldeca-2,6-dien-1-yl)benzenesulfonate

km360-2'(13C)

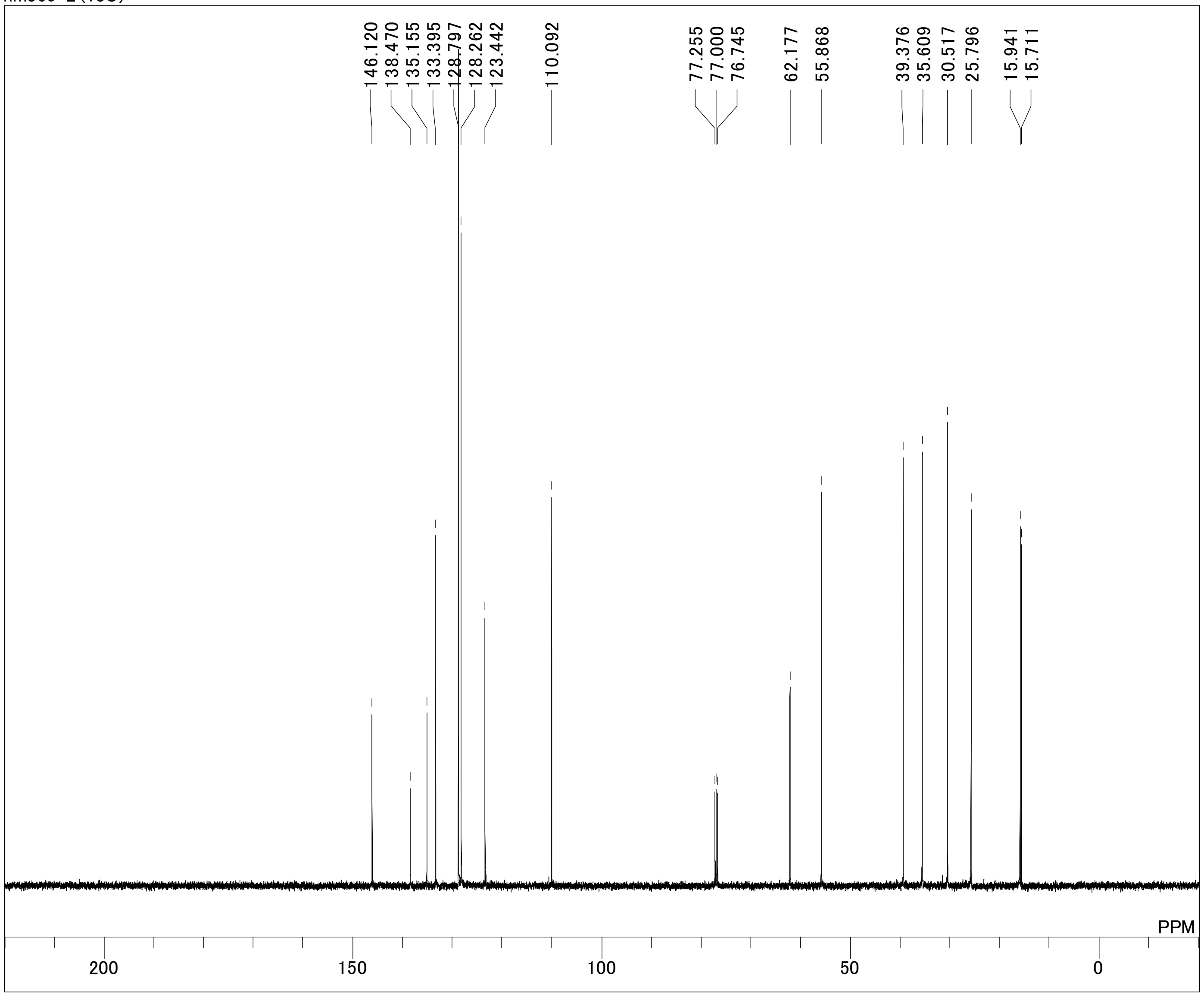

DFILE	F:¥mameda¥NMR¥km360-2'(13C).als
COMNT	km360-2'(13C)
DATIM	Wed Sep 14 20:56:54 2011
OBNUC	13C
EXMOD	bcm
OBFRQ	125.65 MHz
OBSET	0.00 KHz
OBFIN	127958.00 Hz
POINT	32768
FREQU	33898.30 Hz
SCANS	128
ACQTM	0.9667 sec
PD	2.0333 sec
PW1	4.90 usec
IRNUC	1H
CTEMP	28.9 c
SLVNT	CDCL3
EXREF	77.00 ppm
BF	1.20 Hz
RGAIN	30

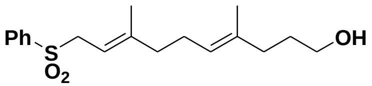

((2*E*,6*E*)-10-Hydroxy-3,7-dimethyldeca-2,6-dien-1-yl)benzenesulfonate

km163-1'

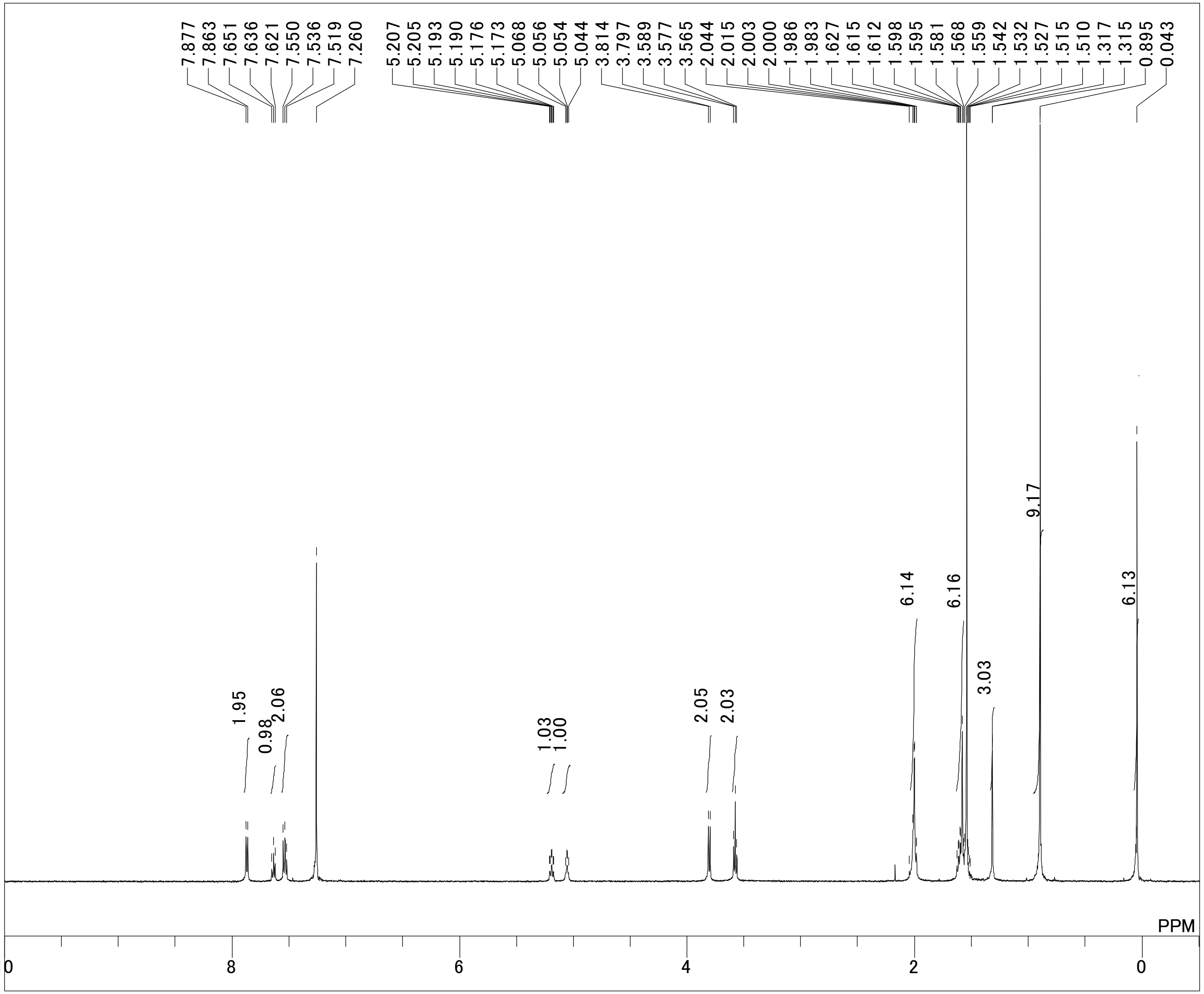

DFILE	F:¥mameda¥NMR¥km163-1'.als
COMNT	km163-1'
DATIM	Mon Aug 08 15:20:37 2011
OBNUC	1H
EXMOD	non
OBFRQ	500.00 MHz
OBSET	0.00 KHz
OBFIN	162160.00 Hz
POINT	8192
FREQU	10000.00 Hz
SCANS	8
ACQTM	0.8192 sec
PD	6.1808 sec
PW1	6.20 usec
IRNUC	1H
CTEMP	26.7 c
SLVNT	CDCL3
EXREF	7.26 ppm
BF	0.12 Hz
RGAIN	25

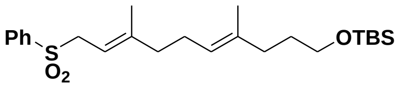

((2*E*,6*E*)-10-(*tert*-Butyldimethylsiloxy)-3,7-dimethyldeca-2,6-dien-1-yl)benzenesulfonate

km163-1'(13C)

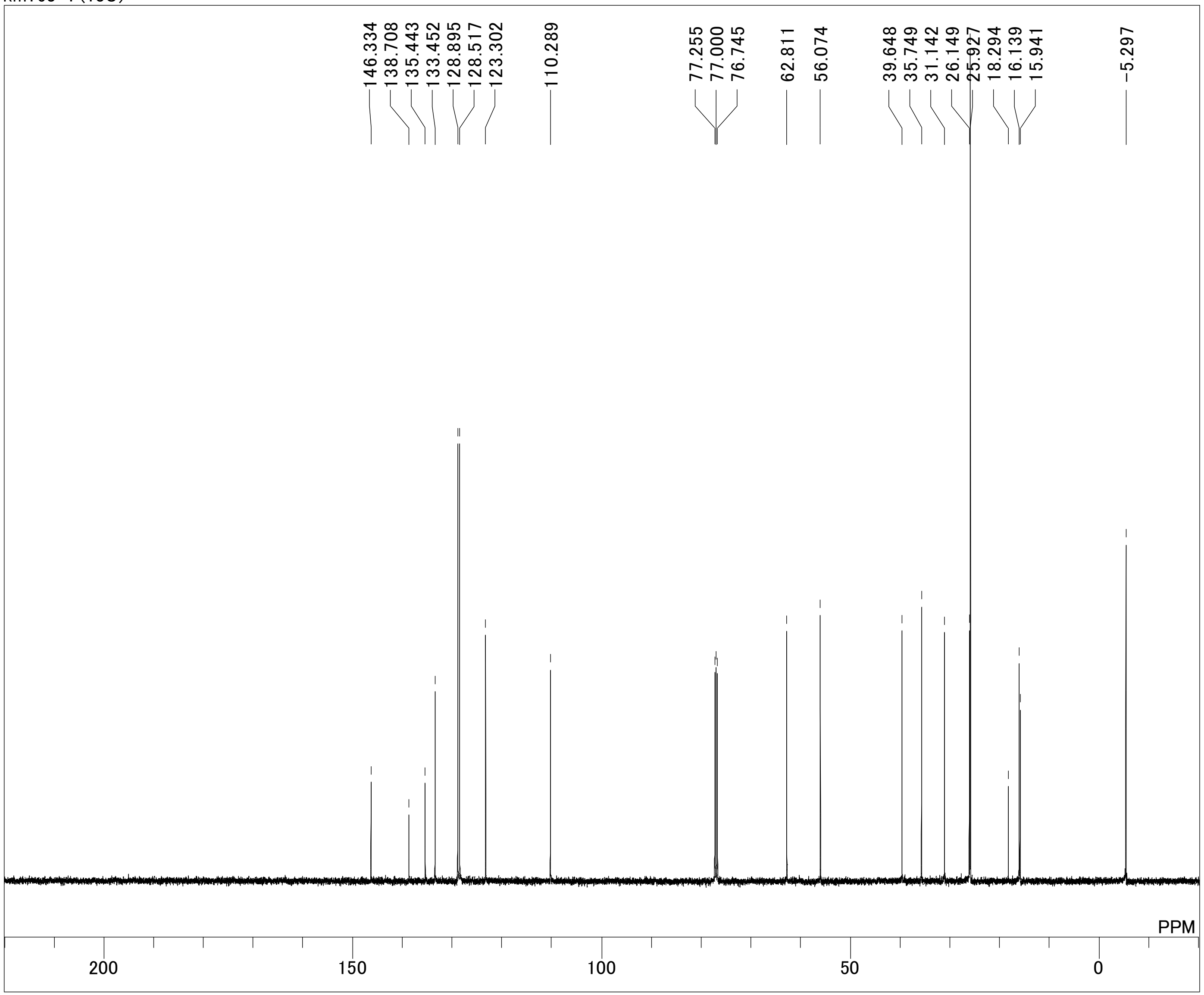

DFILE	F:¥mameda¥NMR¥km163-1'(13C).als
COMNT	km163-1'(13C)
DATIM	Mon Aug 08 15:57:34 2011
OBNUC	13C
EXMOD	bcm
OBFRQ	125.65 MHz
OBSET	0.00 KHz
OBFIN	127958.00 Hz
POINT	32768
FREQU	33898.30 Hz
SCANS	640
ACQTM	0.9667 sec
PD	2.0333 sec
PW1	4.90 usec
IRNUC	1H
CTEMP	29.2 c
SLVNT	CDCL3
EXREF	77.00 ppm
BF	1.20 Hz
RGAIN	31

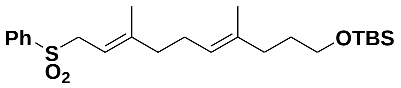

((2*E*,6*E*)-10-(*tert*-Butyldimethylsiloxy)-3,7-dimethyldeca-2,6-dien-1-yl)benzenesulfonate

km367-1'

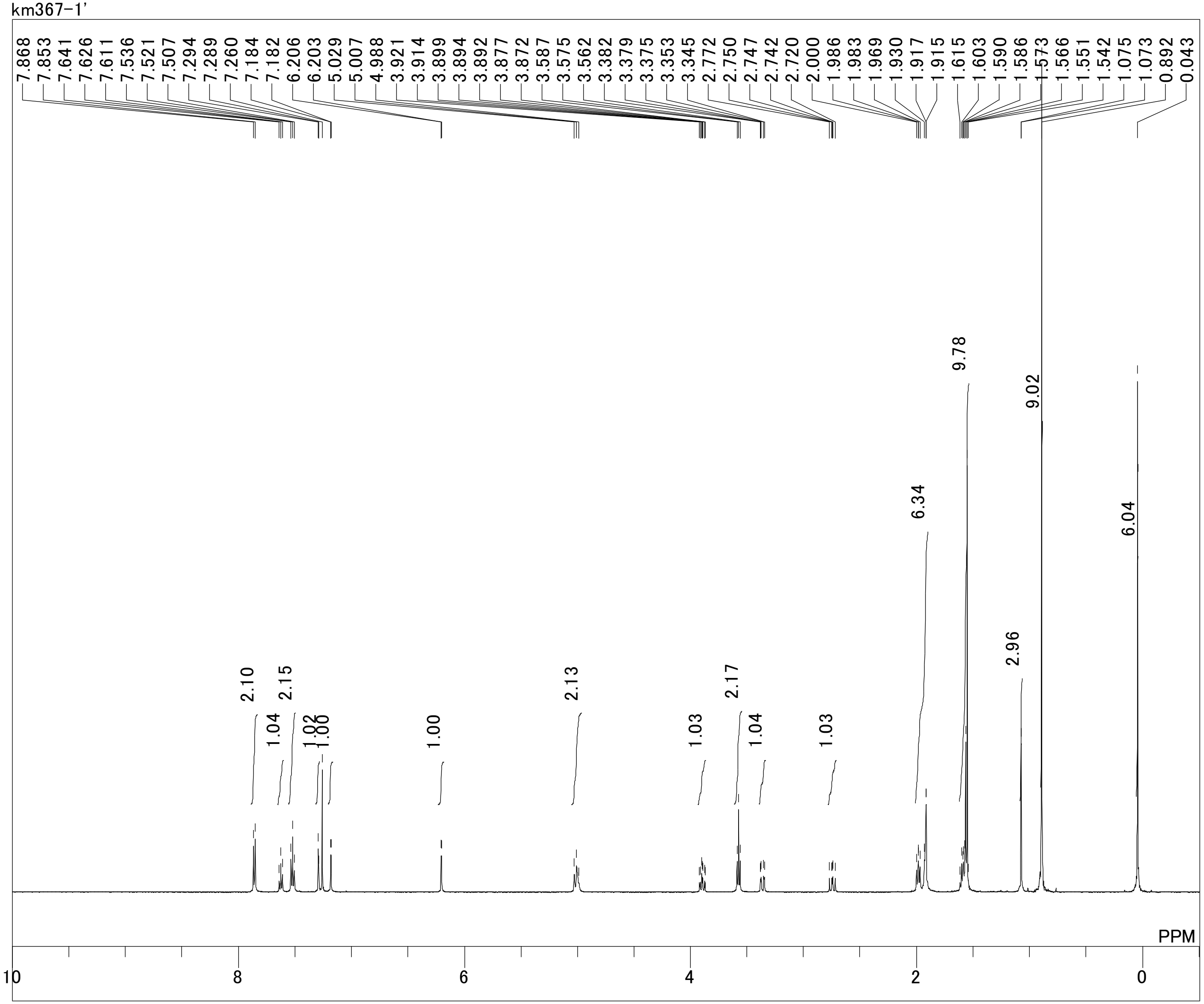

DFILE	F:¥mameda¥NMR¥km367-1'.als
COMNT	km367-1'
DATIM	Sun Sep 18 17:39:17 2011
OBNUC	1H
EXMOD	non
OBFRQ	500.00 MHz
OBSET	0.00 KHz
OBFIN	162160.00 Hz
POINT	8192
FREQU	10000.00 Hz
SCANS	8
ACQTM	0.8192 sec
PD	6.1808 sec
PW1	6.20 usec
IRNUC	1H
CTEMP	27.1 c
SLVNT	CDCL3
EXREF	7.26 ppm
BF	0.12 Hz
RGAIN	22

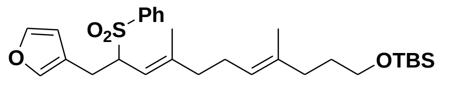

((2*E*,6*E*)-10-(*tert*-Butyldimethylsiloxy)-1-(furan-3-yl)methyl-3,7-dimethyldeca-2,6-dien-1-yl)benzenesulfonate

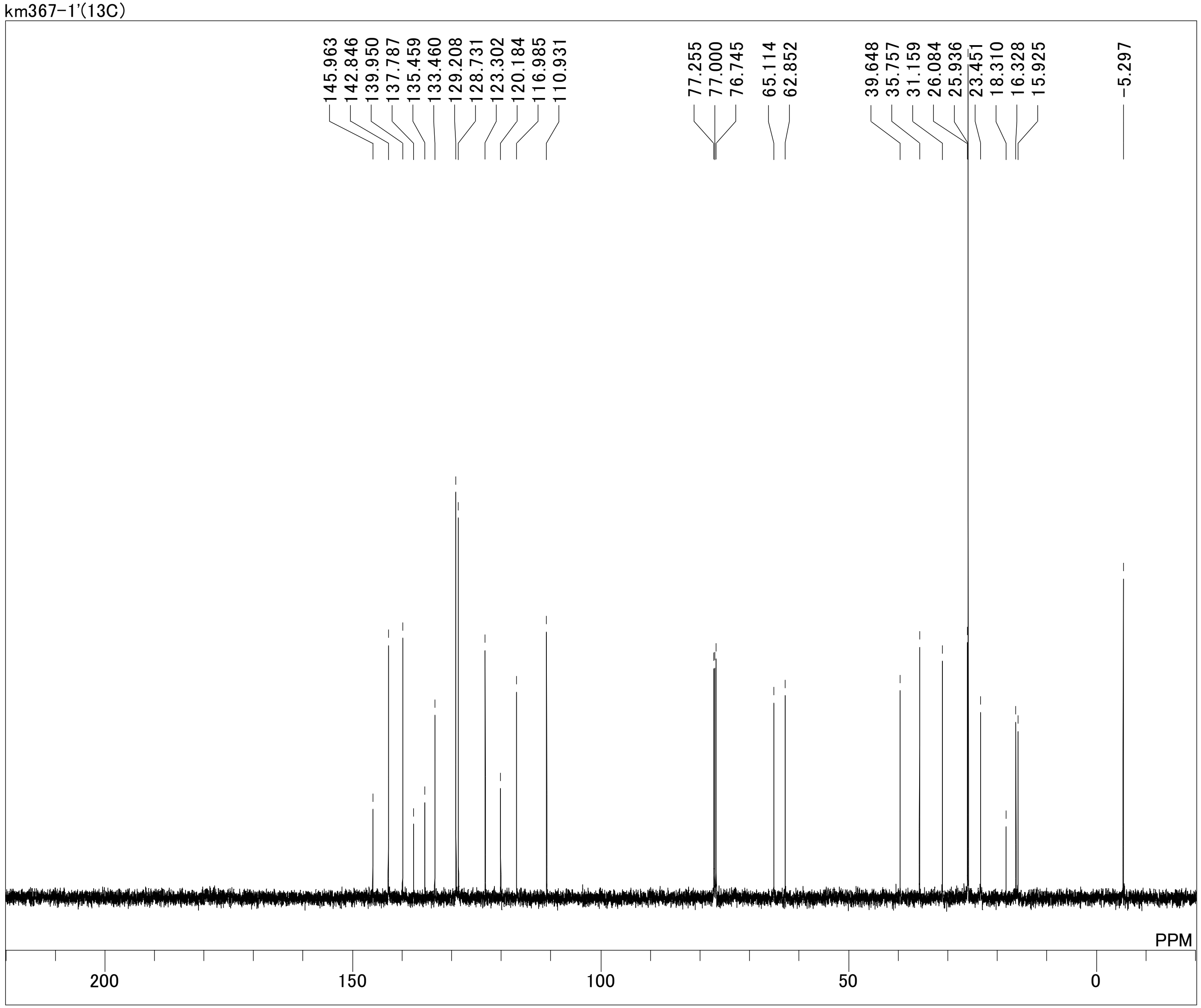

DFILE	F:¥mameda¥NMR¥km367-1'(13C).als
COMNT	km367-1'(13C)
DATIM	Sun Sep 18 17:48:47 2011
OBNUC	13C
EXMOD	bcm
OBFRQ	125.65 MHz
OBSET	0.00 KHz
OBFIN	127958.00 Hz
POINT	32768
FREQU	33898.30 Hz
SCANS	128
ACQTM	0.9667 sec
PD	2.0333 sec
PW1	4.90 usec
IRNUC	1H
CTEMP	28.4 c
SLVNT	CDCL3
EXREF	77.00 ppm
BF	1.20 Hz
RGAIN	31

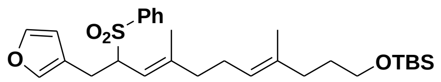

((2*E*,6*E*)-10-(*tert*-Butyldimethylsiloxy)-1-(furan-3-yl)methyl-3,7-dimethyldeca-2,6-dien-1-yl)benzenesulfonate

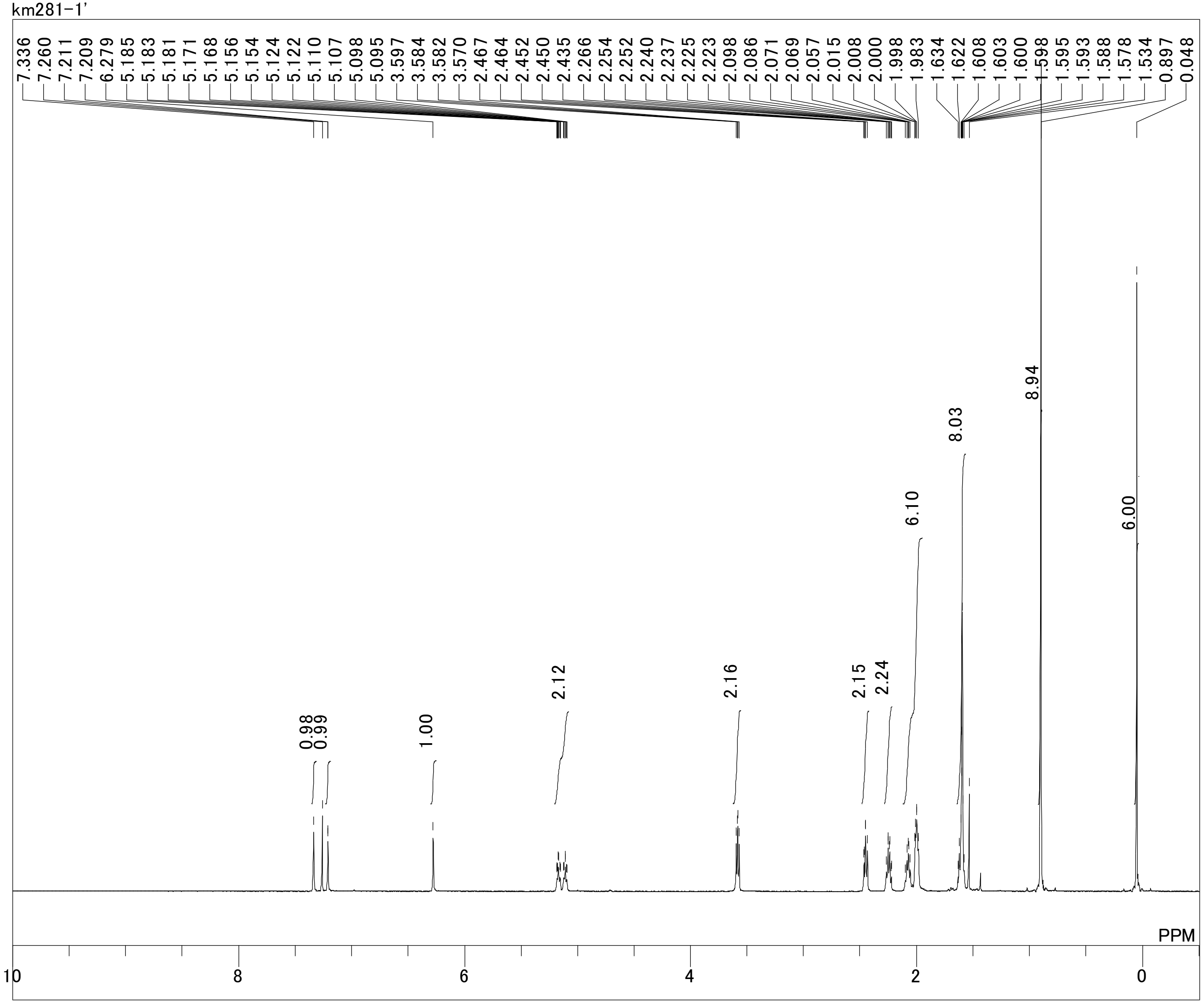

DFILE	F:¥mameda¥NMR¥km281-1'.als
COMNT	km281-1'
DATIM	Fri Aug 05 21:16:35 2011
OBNUC	1H
EXMOD	non
OBFRQ	500.00 MHz
OBSET	0.00 KHz
OBFIN	162160.00 Hz
POINT	8192
FREQU	10000.00 Hz
SCANS	8
ACQTM	0.8192 sec
PD	6.1808 sec
PW1	6.20 usec
IRNUC	1H
CTEMP	28.2 c
SLVNT	CDCL3
EXREF	7.26 ppm
BF	0.12 Hz
RGAIN	19

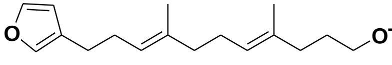

((4*E*,8*E*)-1-(*tert*-Butyldimethylsiloxy)-4,8-dimethylundeca-4,8-dien-11-yl)furan

km281-1'(13C)

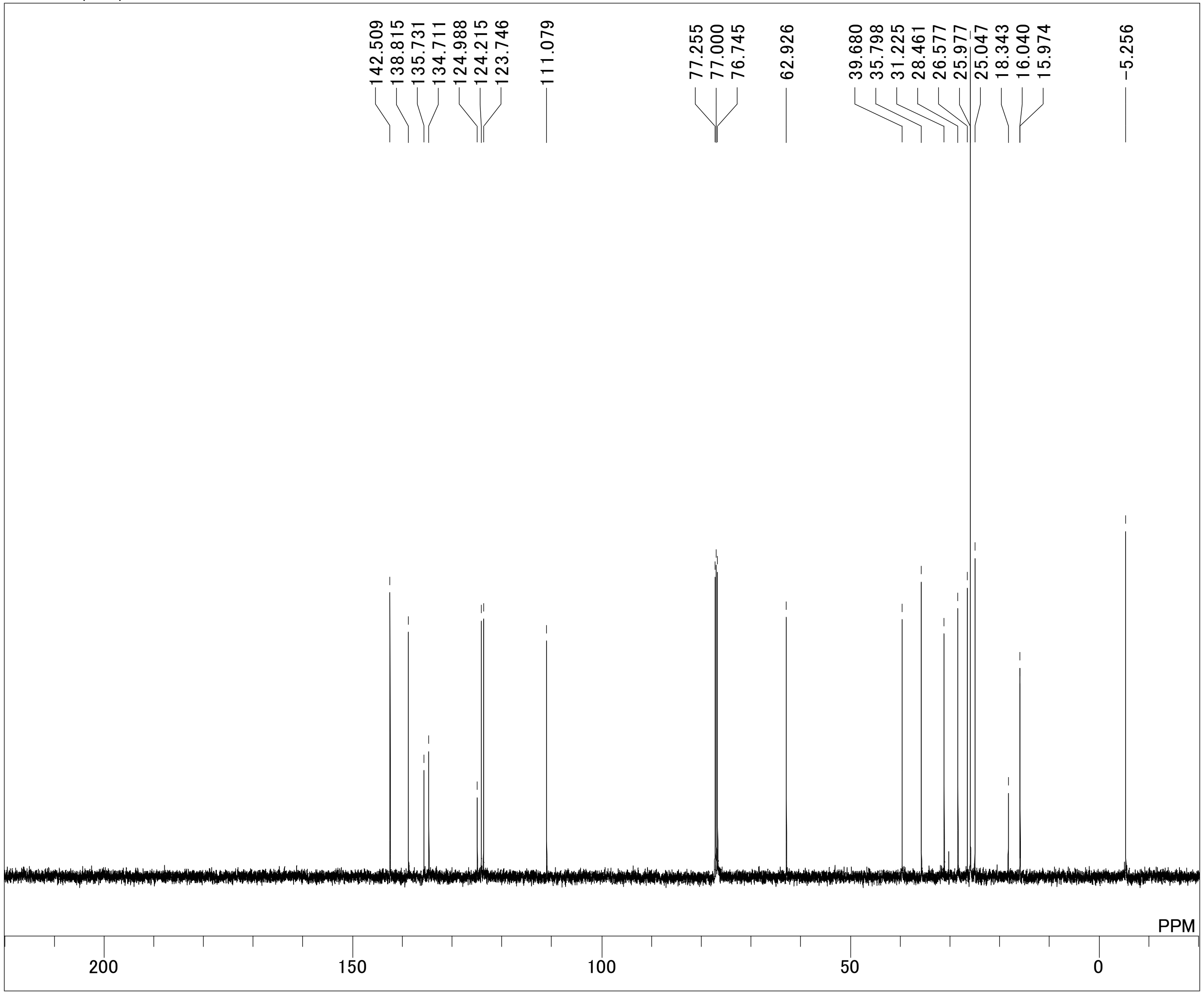

DFILE	F:¥mameda¥NMR¥km281-1'(13C).als
COMNT	km281-1'(13C)
DATIM	Fri Aug 05 21:58:44 2011
OBNUC	13C
EXMOD	bcm
OBFRQ	125.65 MHz
OBSET	0.00 KHz
OBFIN	127958.00 Hz
POINT	32768
FREQU	33898.30 Hz
SCANS	384
ACQTM	0.9667 sec
PD	2.0333 sec
PW1	4.90 usec
IRNUC	1H
CTEMP	29.3 c
SLVNT	CDCL3
EXREF	77.00 ppm
BF	1.20 Hz
RGAIN	31

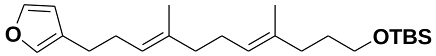

((4*E*,8*E*)-1-(*tert*-Butyldimethylsiloxy)-4,8-dimethylundeca-4,8-dien-11-yl)furan

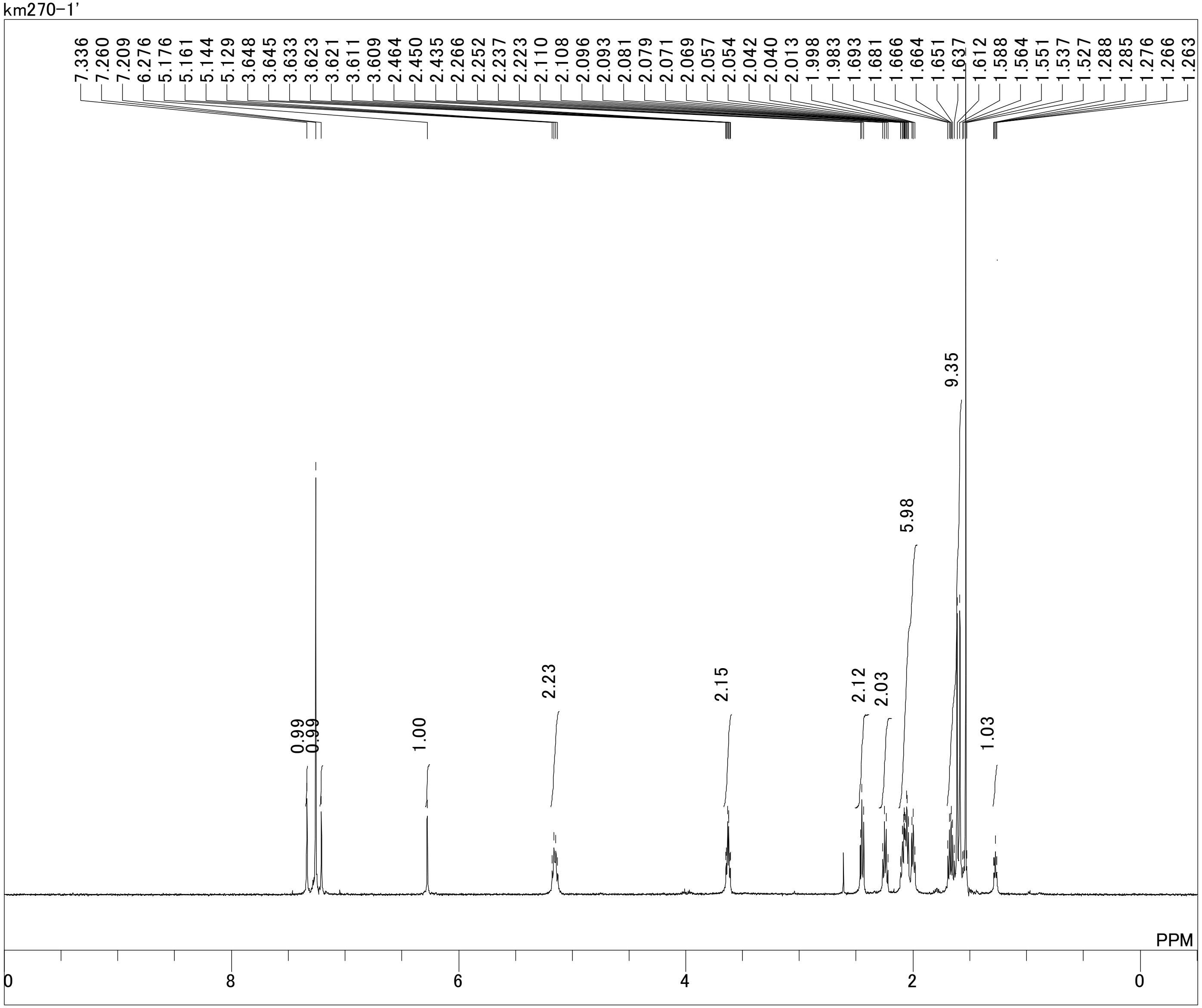

DFILE F:¥mameda¥NMR¥km270-1'.als
COMNT km270-1'
DATIM Mon Aug 08 11:23:33 2011
OBNUC 1H
EXMOD non
OBFRQ 500.00 MHz
OBSET 0.00 KHz
OBFIN 162160.00 Hz
POINT 8192
FREQU 10000.00 Hz
SCANS 8
ACQTM 0.8192 sec
PD 6.1808 sec
PW1 6.20 usec
IRNUC 1H
CTEMP 26.7 c
SLVNT CDCL3
EXREF 7.26 ppm
BF 0.12 Hz
RGAIN 25

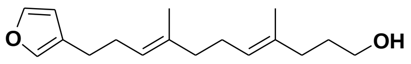

(*4E*,*8E*)-11-(Furan-3-yl)-4,8-dimethyl-4,8-undecadien-1-ol (**13**)

km270-1'(13C)

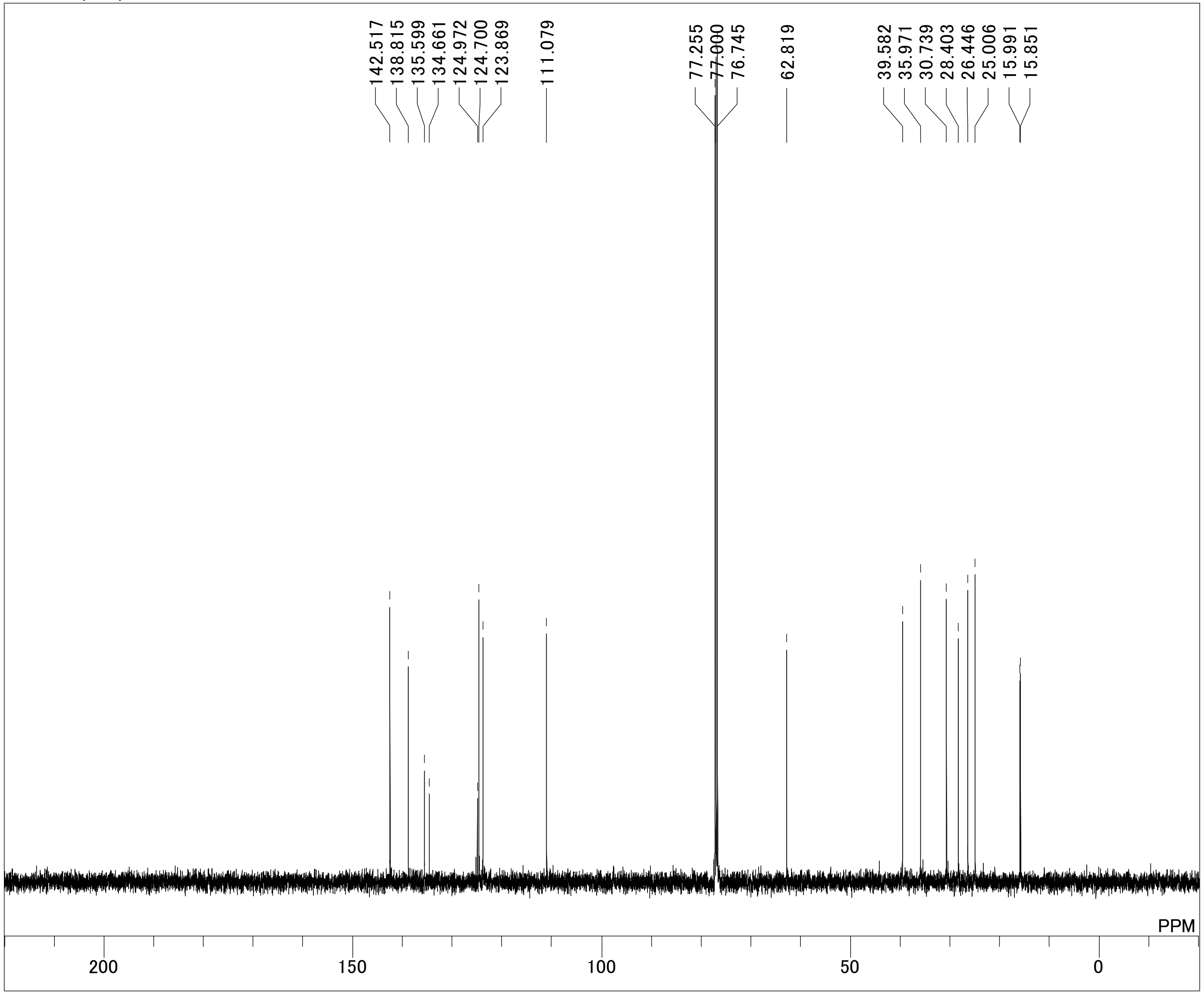

DFILE	F:¥mameda¥NMR¥km270-1'(13C).als
COMNT	km270-1'(13C)
DATIM	Mon Aug 08 12:07:53 2011
OBNUC	13C
EXMOD	bcm
OBFRQ	125.65 MHz
OBSET	0.00 KHz
OBFIN	127958.00 Hz
POINT	32768
FREQU	33898.30 Hz
SCANS	640
ACQTM	0.9667 sec
PD	2.0333 sec
PW1	4.90 usec
IRNUC	1H
CTEMP	29.0 c
SLVNT	CDCL3
EXREF	77.00 ppm
BF	1.20 Hz
RGAIN	31

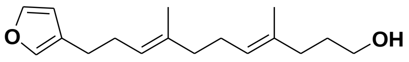

(4*E*,8*E*)-11-(Furan-3-yl)-4,8-dimethyl-4,8-undecadien-1-ol (**13**)
